# Supplementary material for: Scaffold-hopping for molecular glues targeting the 14-3-3/ERα complex
Source: Nat Commun. 2025 Jul 14;16:6467. doi: 10.1038/s41467-025-61176-4 (PMC12260087; doi:10.1038/s41467-025-61176-4)

Scaffold-hopping for molecular glues targeting the 14-3-3/ERα complex

Markella Konstantinidou*[1], Marios Zingiridis[2], Marloes A.M. Pennings[3], Michael Fragkiadakis[2], Johanna M. Virta[1], Jezrael L. Revalde[1], Emira J. Visser[3], Christian Ottmann[3], Luc Brunsveld[3], Constantinos G. Neochoritis*[2], Michelle R. Arkin*[1]

[1] M. Konstantinidou, J.M. Virta, J.L. Revalde, M.R. Arkin
Department of Pharmaceutical Chemistry and Small Molecule Discovery Centre (SMDC)
University of California San Francisco (UCSF)
CA 94143 (USA)
E-mail: markella.konstantinidou@ucsf.edu, michelle.arkin@ucsf.edu

[2] M. Zingiridis, M. Fragkiadakis, C.G. Neochoritis
Department of Chemistry
University of Crete
Voutes, Heraklion, 70013, Greece
E-mail: kneochor@uoc.gr

[3] M.A.M. Pennings, E.J. Visser, C. Ottmann, L. Brunsveld
Laboratory of Chemical Biology, Department of Biomedical Engineering and Institute for Complex Molecular Systems (ICMS)
Eindhoven University of Technology, 5600 MB Eindhoven, The Netherlands

Table of Contents

**1. Supplementary Figures**	
Supplementary Figures 1 – 14	p2– 11
**2. Supplementary Tables**	
Supplementary Table 1: Overview of molecular structures of compounds and assays	p12 – 14
Supplementary Table 2: MS data	p15
Supplementary Table 3: TR-FRET data	p16
Supplementary Table 4: SPR data	p17
Supplementary Table 5: NanoBRET data	p18
Supplementary Table 6: Crystallography data collection parameters	p19 – 23
**3. Supplementary Methods**	p24 – 65

1. SUPPLEMENTARY FIGURES

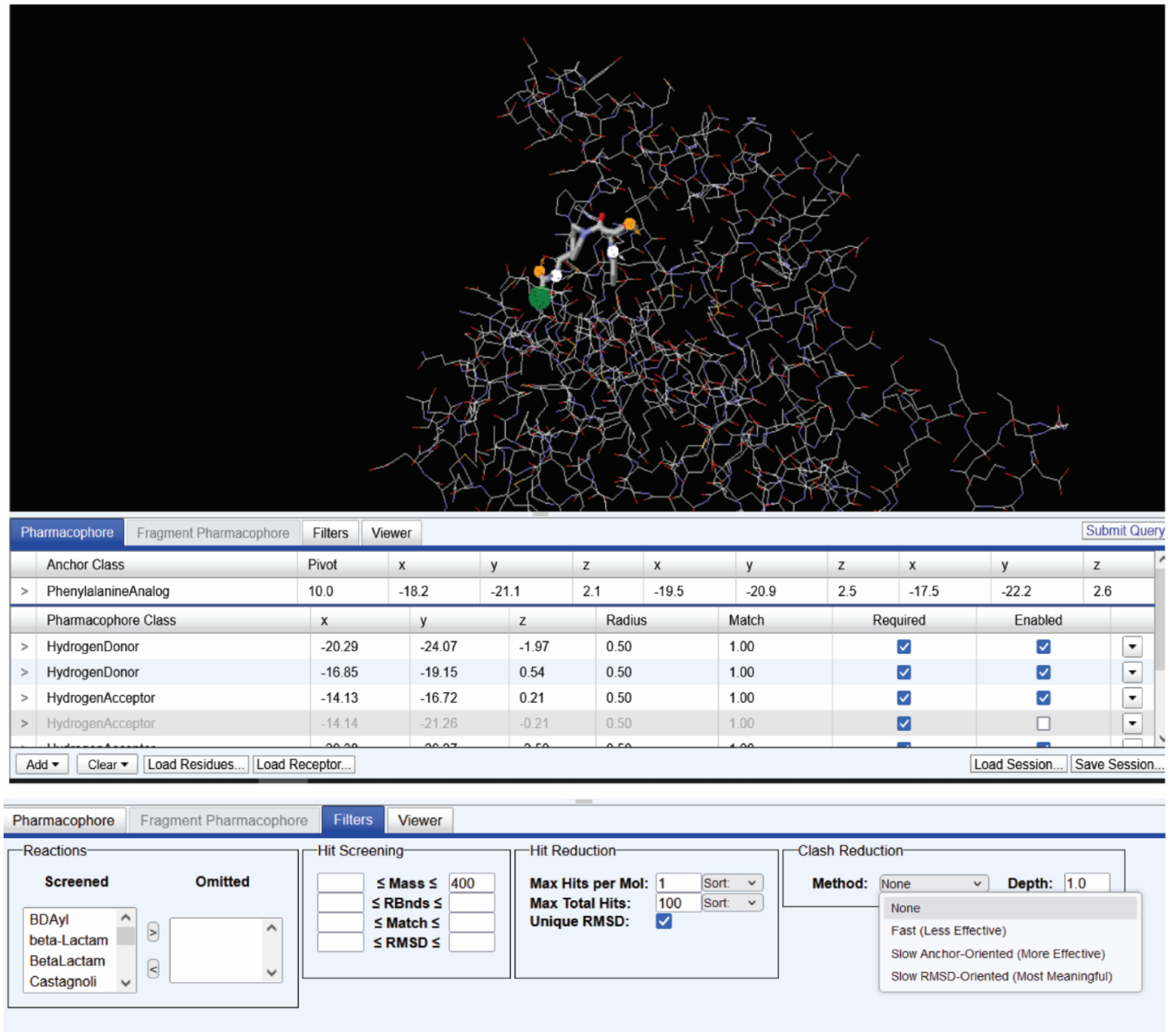

Supplementary Figure 1. Overview of AnchorQuery^{TM}. The crystal structure of compound **127** bound to the 14-3-3σ/ERα complex (PDB 8ALW) was used as a starting point for the design of an MCR scaffold.

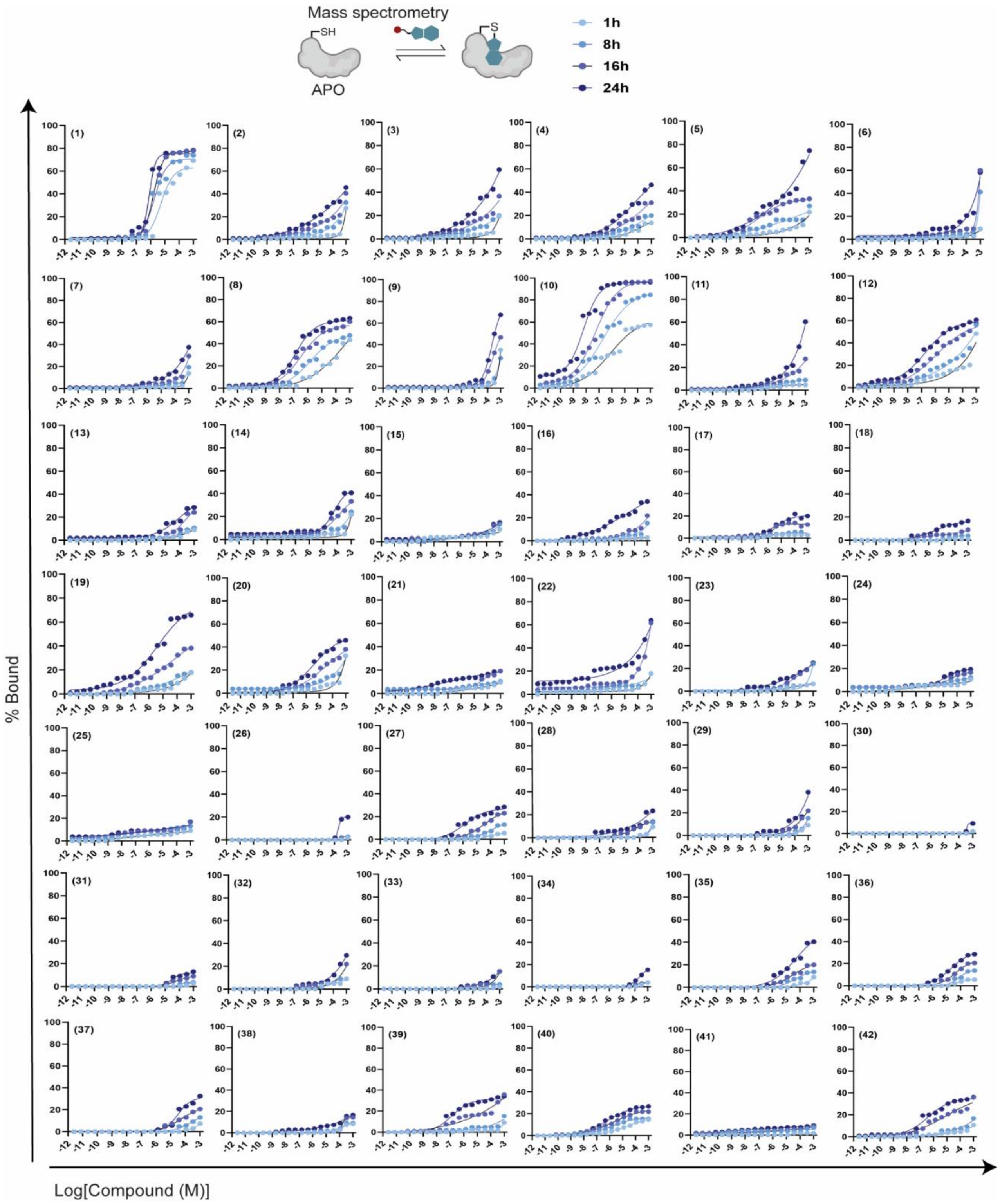

Supplementary Figure 2. MS dose-response curves (apo).

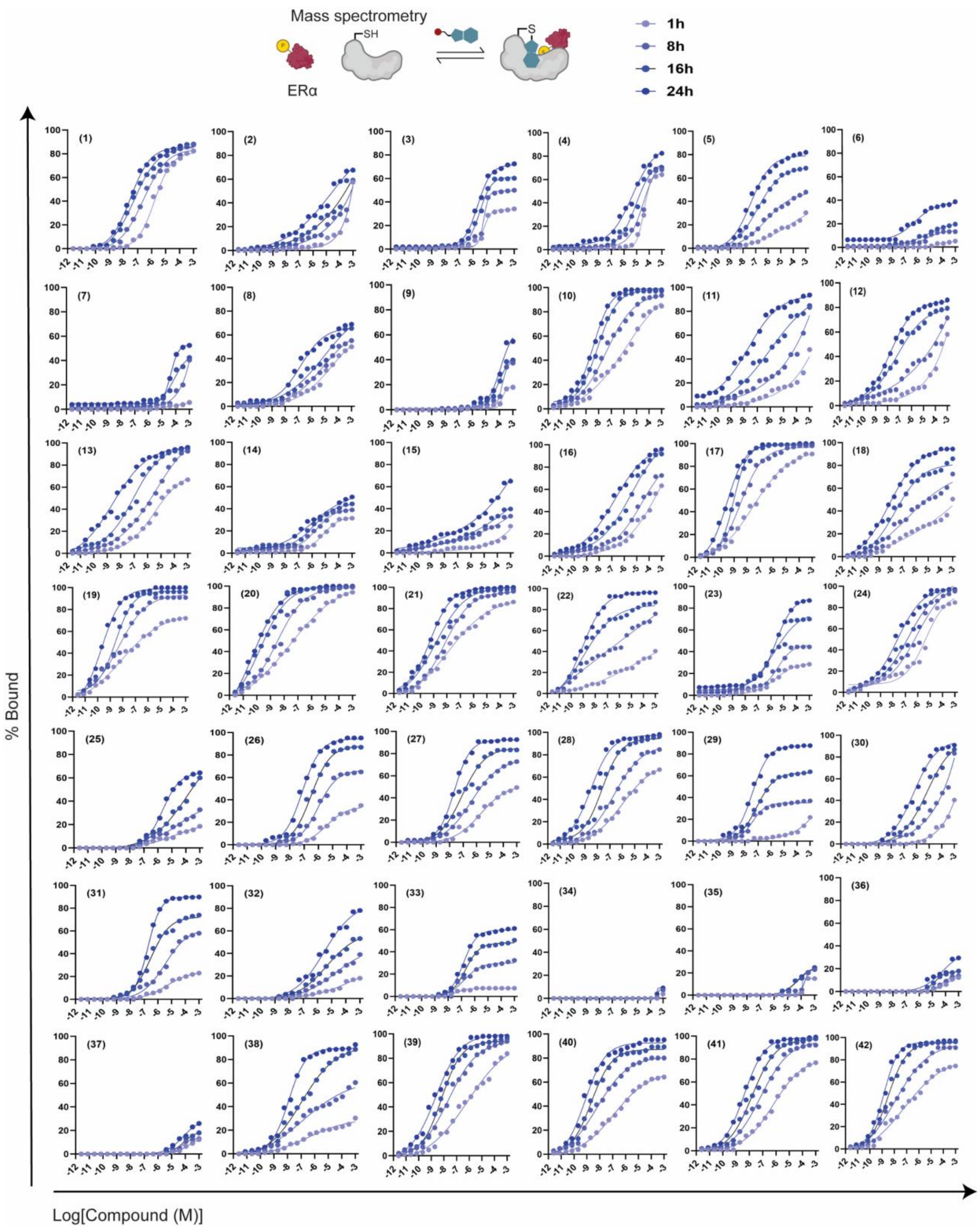

Supplementary Figure 3. MS dose-response curves (ERα).

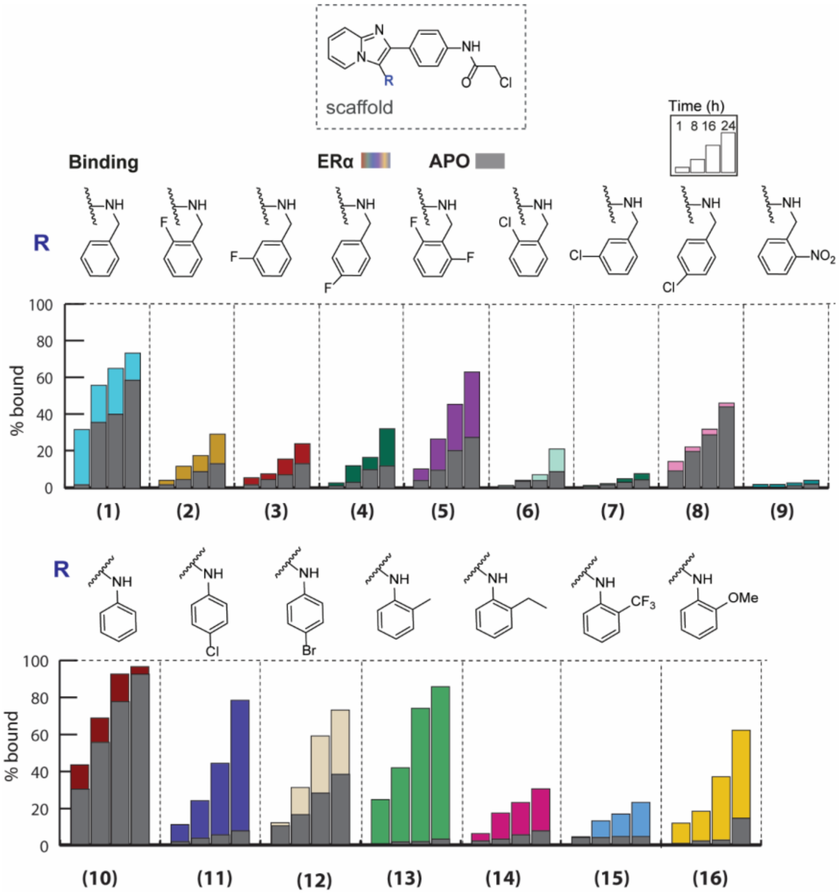

Supplementary Figure 4. SAR of benzyl and phenyl analogs. MS bar graphs at 1 μM compound. For each compound, time course experiments were performed with measurements at 1h, 8h, 16h and 24h. ERα data are shown with different colors for each compound, and apo data in gray.

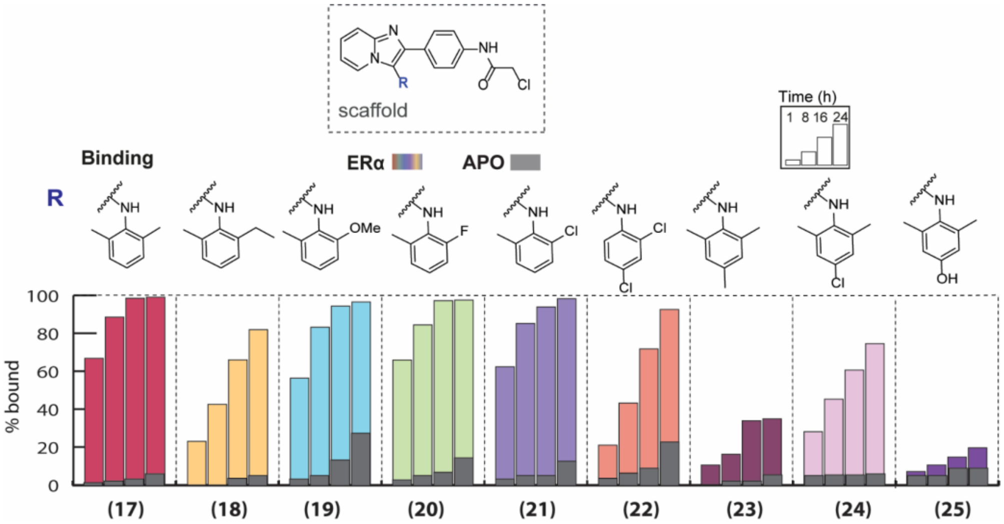

Supplementary Figure 5. SAR of analogs with double *o*-substitutions. MS bar graphs at 1 μM compound. For each compound, time course experiments were performed with measurements at 1h, 8h, 16h and 24h. ERα data are shown with different colors for each compound, and apo data in gray.

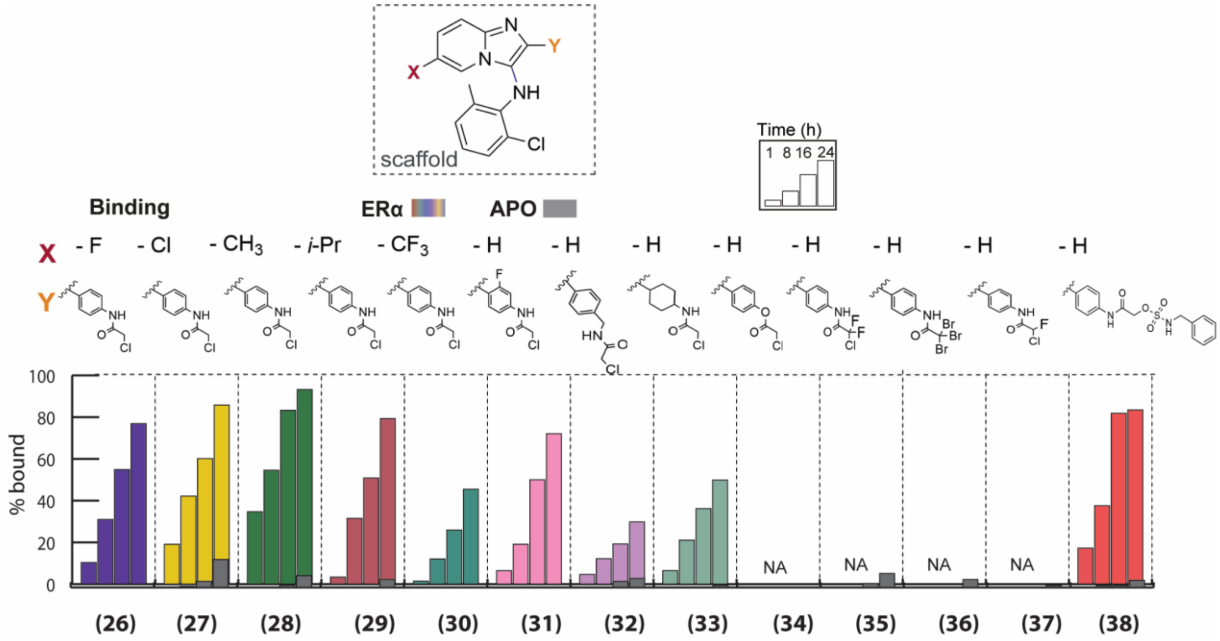

Supplementary Figure 6. SAR of analogs varying positions X and Y on the scaffold. MS bar graphs at 1 µM compound. For each compound, time course experiments were performed with measurements at 1h, 8h, 16h and 24h. ERα data are shown with different colors for each compound, and apo data in gray.

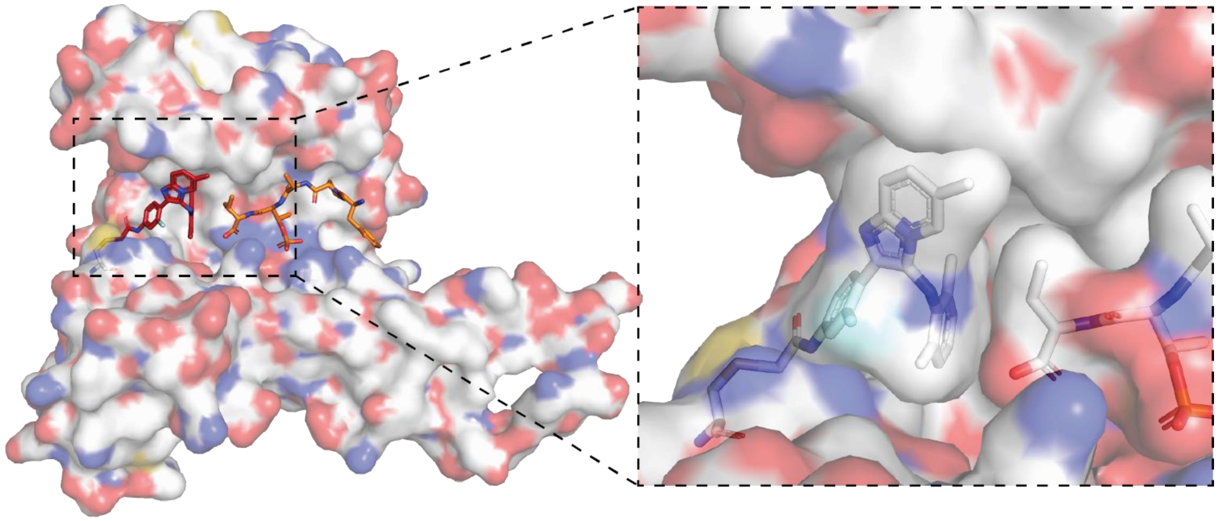

Supplementary Figure 7. Surface representation of 14-3-3σ bound to the ERα peptide and compound **41**, colored by atom (carbon = white, oxygen = red, nitrogen = blue, sulfur = yellow, fluoro = cyan). The groove occupied by the compound was primarily hydrophobic. Polar amino acids were located at the rim of the interface. The close-up surface representation of the compound indicated favorable shape complementarity with the 14-3-3σ/ERα complex.

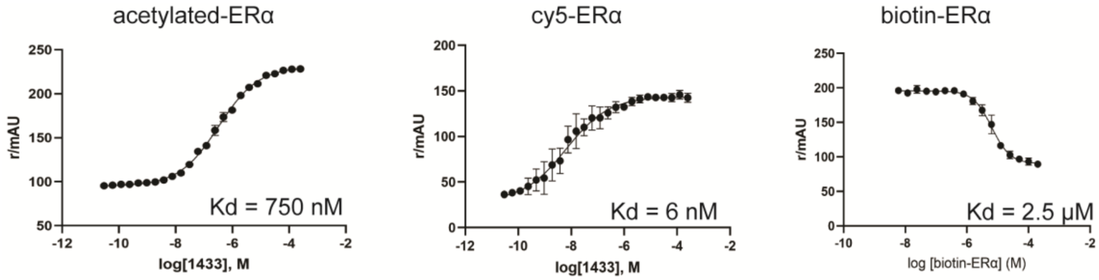

Supplementary Figure 8. K_d determination for FAM-, cy5- and biotin-labeled ERα peptides.

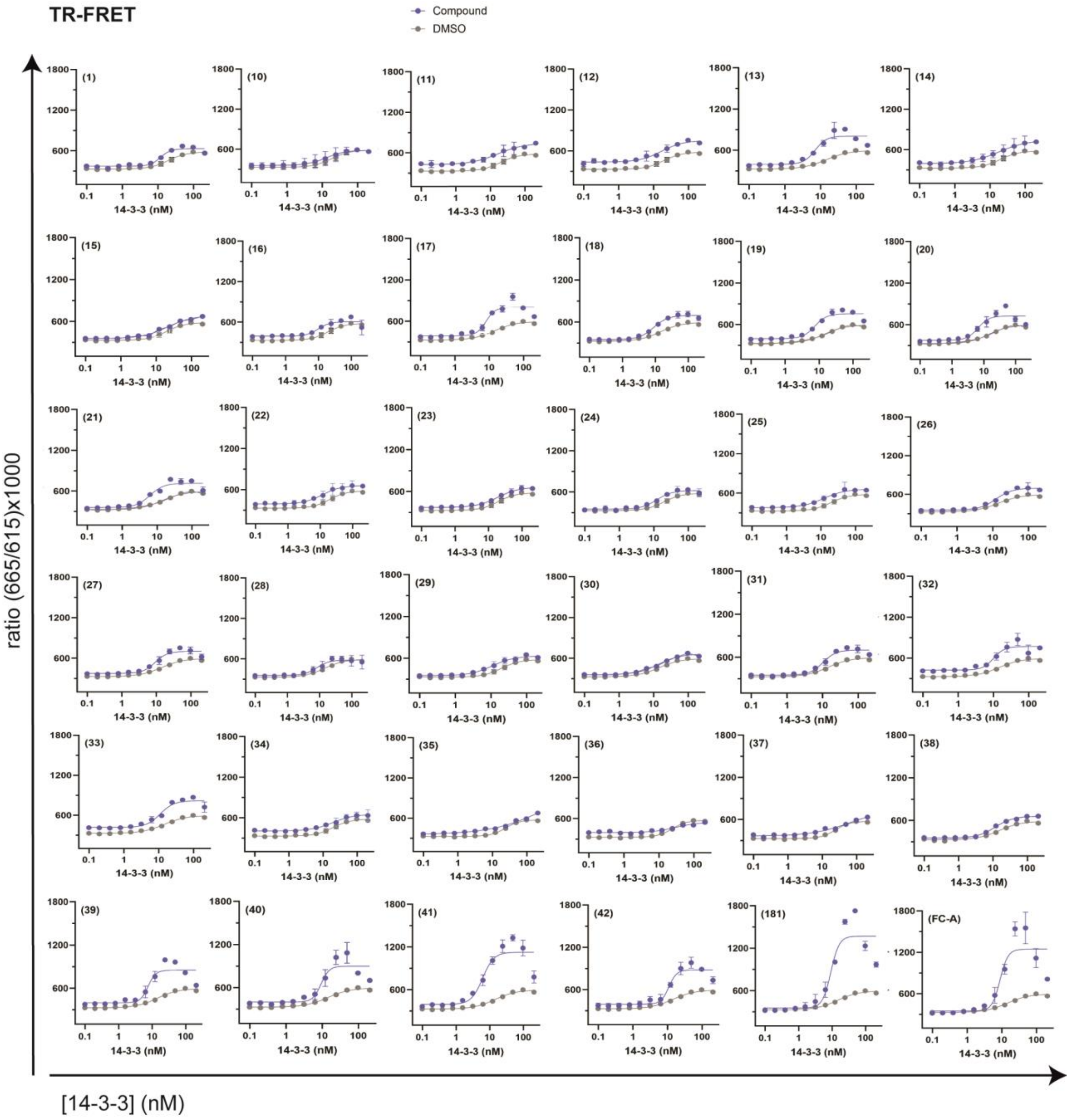

Supplementary Figure 9. TR-FRET protein titrations. For each compound three independent experiments were performed (n=3).

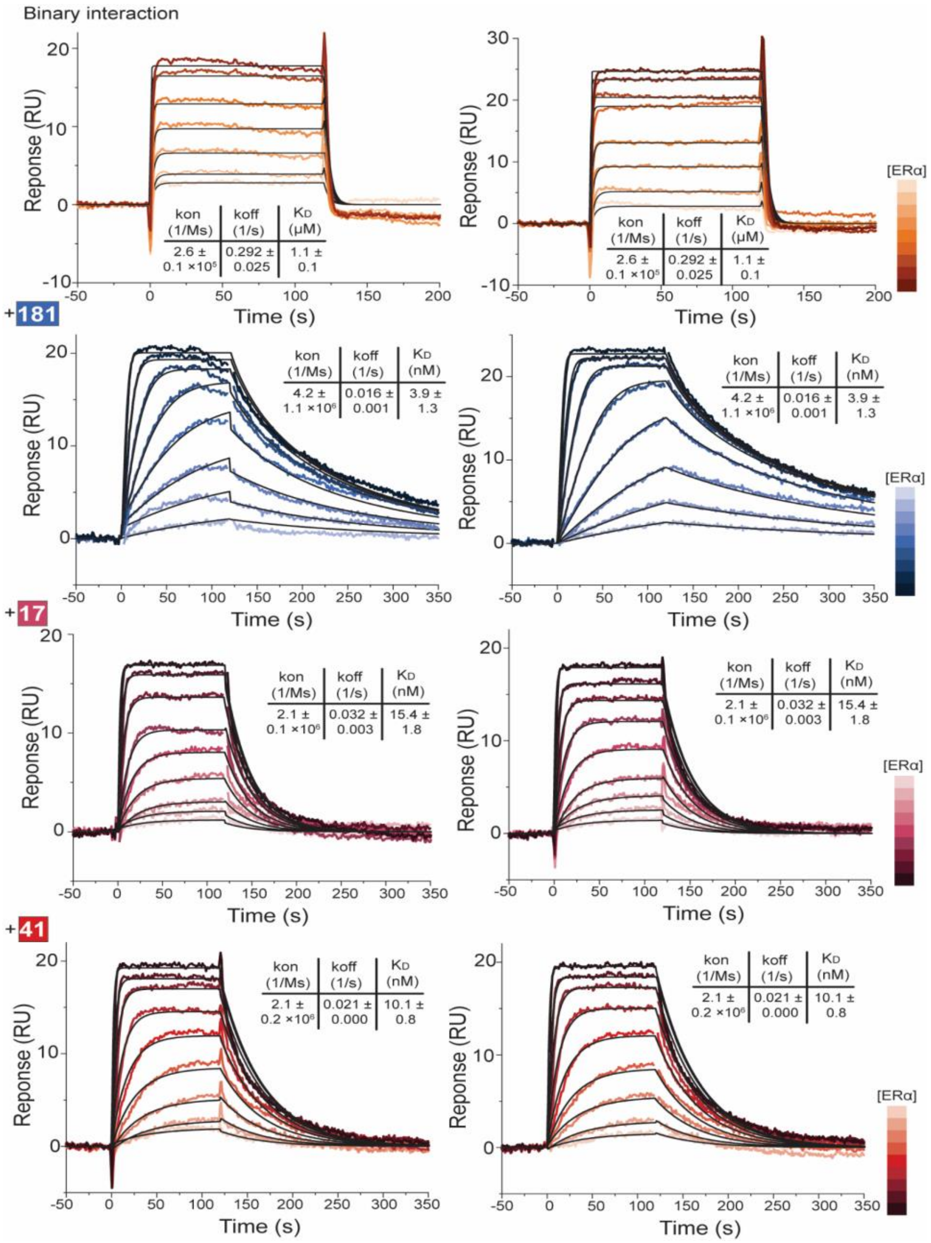

Supplementary Figure 10. SPR replicates for the binary 14-3-3σ/ERα interaction and ternary interactions with **181**, **17** and **41.**

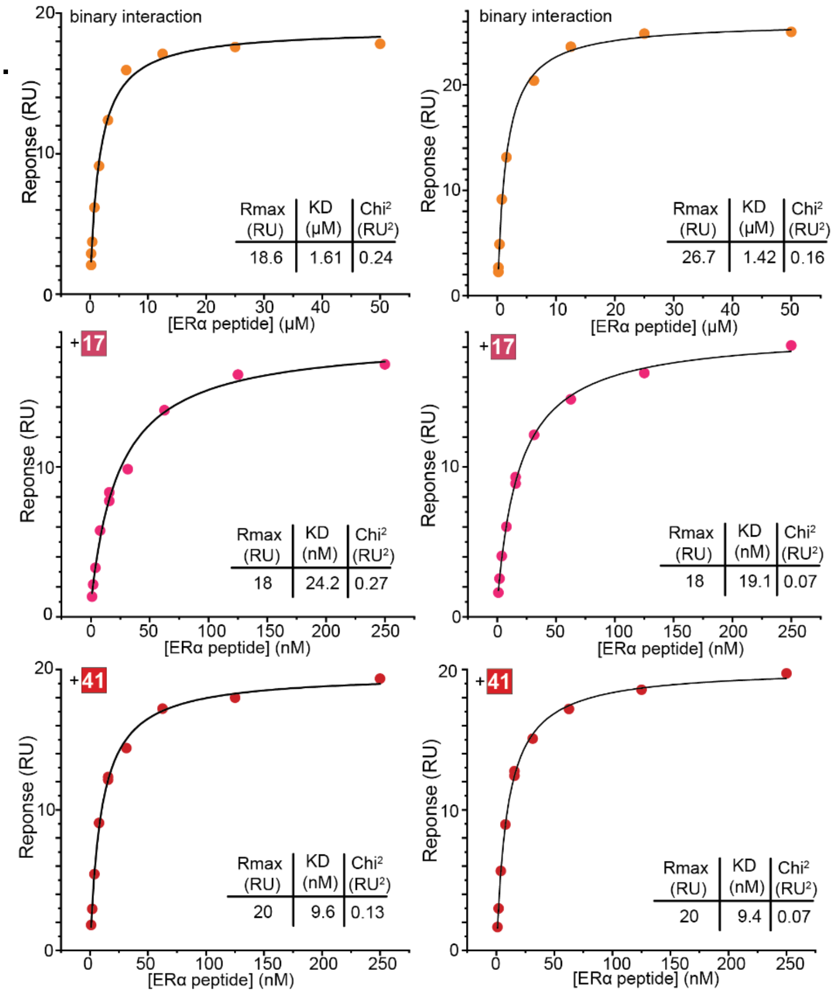

Supplementary Figure 11. Affinity fits of sensorgrams in SI Figure 10 that reached equilibrium by plotting the response (RU) over the ERα peptide concentration (μM for the binary interaction, nM for compounds **17** and **41**). The Rmax, KD value and Chi^2 are reported for each replicate.

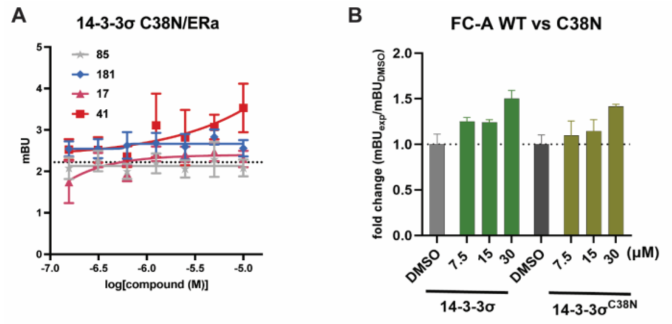

Supplementary Figure 12. A) 14-3-3σ^{C38N}/ERα NanoBRET. 1:10 ratio of NanoLuc- ERα:14-3-3σ^{C38N}-HaloTag plasmids. Cells treated with compound for 24 hours. B) 14-3-3σ/ERα NanoBRET and 14-3-3σ^{C38N}/ERα NanoBRET for the natural product FC-A, indicating that the effect is not specific to the sigma isoform. Data points presented are mean values +/- SD (n=3 technical)

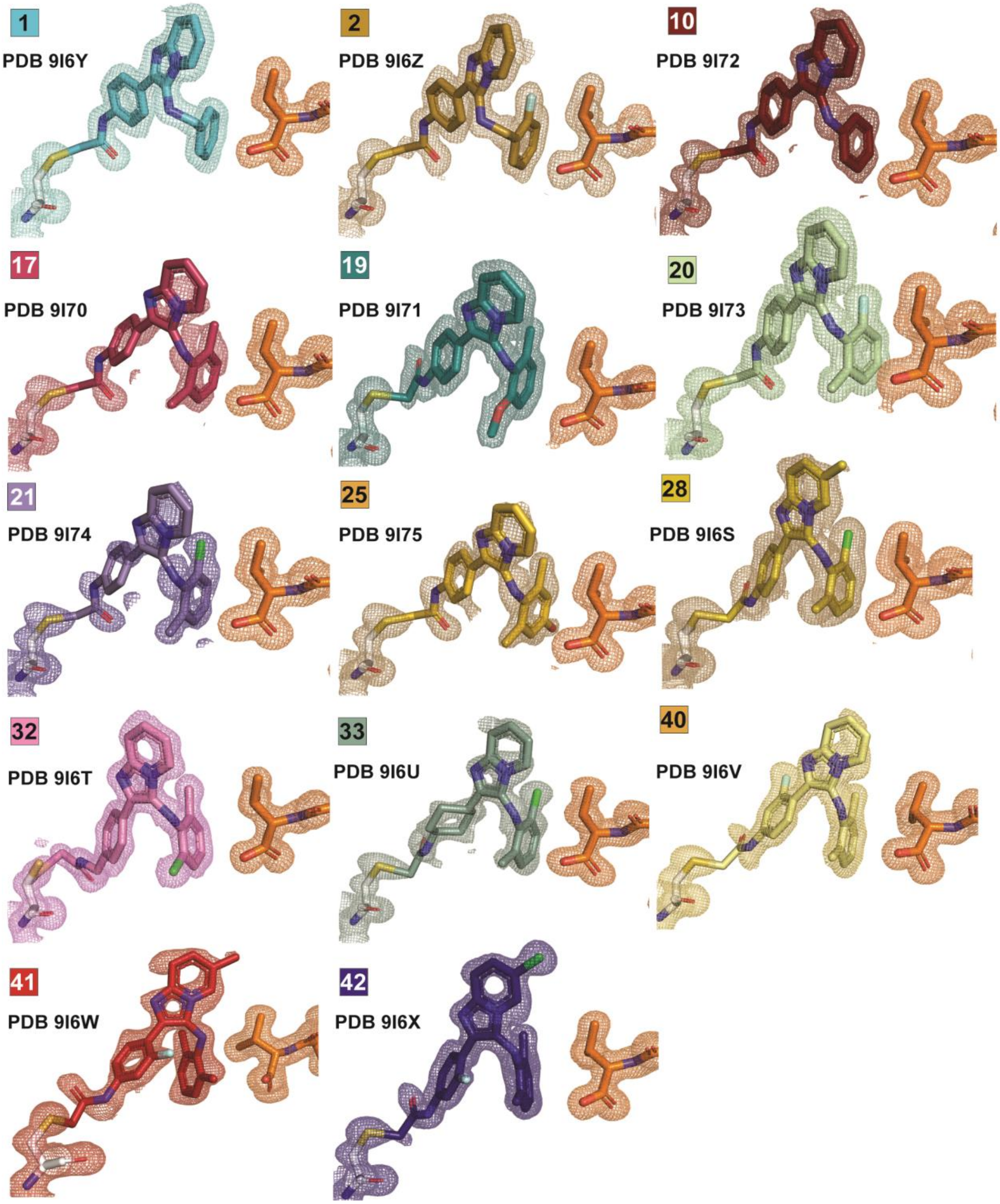

Supplementary Figure 13. Densities and PDB IDs of MCR molecular glues (represented as sticks, compound number at top left). 2Fo-Fc electron density maps (blue mesh) are contoured at 1σ. Crystallographic statistics are listed in table S6.

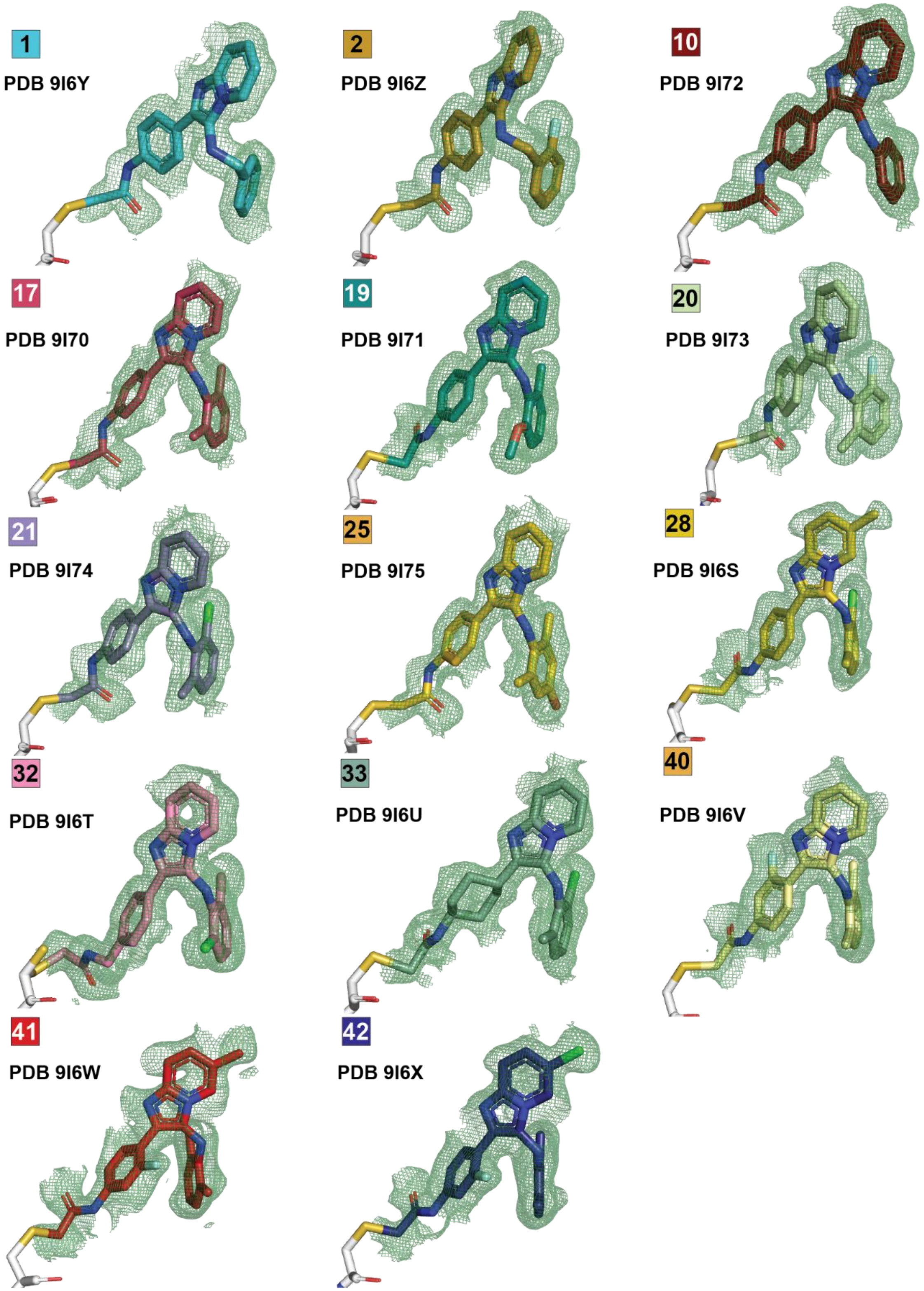

Supplementary Figure 14. Omit density maps (Fo-Fc) of MCR molecular glues (represented as sticks, compound number at top left), which are contoured at 1σ. Crystallographic statistics are listed in table S6.

2. SUPPLEMENTARY TABLES

Supplementary Table 1. Structures of compounds, assays performed and table number for the assays.

No.	SMDC ID	STRUCTURE	MS	TR-FRET	Nano-BRET	Crystallography
**(1)**	**1076388**	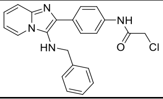	S2	S3	-	S6
**(2)**	**1083860**	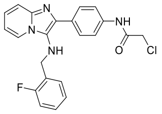	S2	-	-	S6
**(3)**	**1083861**	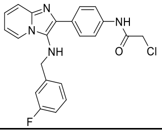	S2	-	-	-
**(4)**	**1083857**	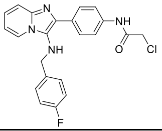	S2	-	-	-
**(5)**	**1084657**	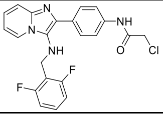	S2	-	-	-
**(6)**	**1083858**	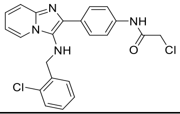	S2	-	-	-
**(7)**	**1083859**	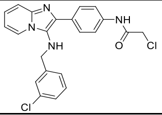	S2	-	-	-
**(8)**	**1084659**	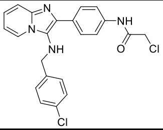	S2	-	-	-
**(9)**	**1083866**	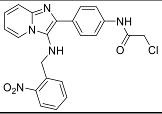	S2	-	-	-
**(10)**	**1124905**	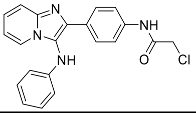	S2	S3	S5	S6
**(11)**	**1083865**	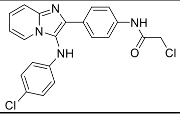	S2	S3	-	-
**(12)**	**1084656**	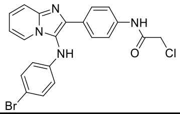	S2	S3	-	-
**(13)**	**1124899**	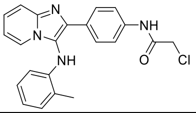	S2	S3	S5	-

**(14)**	**1084652**	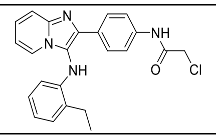	S2	S3	-	-
**(15)**	**1124900**	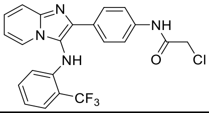	S2	S3	-	-
**(16)**	**1124902**	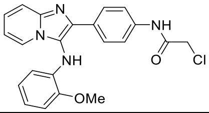	S2	S3	-	-
**(17)**	**1084662**	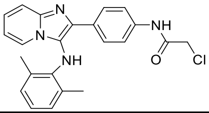	S2	S3	S5	S6
**(18)**	**1124901**	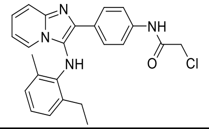	S2	S3	-	-
**(19)**	**1124904**	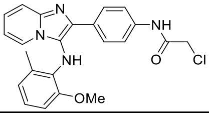	S2	S3	-	S6
**(20)**	**1124906**	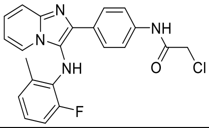	S2	S3	-	S6
**(21)**	**1124907**	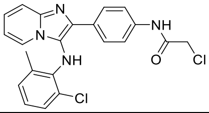	S2	S3	-	S6
**(22)**	**1084654**	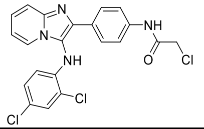	S2	S3	-	-
**(23)**	**1124903**	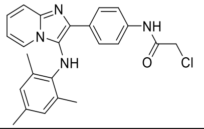	S2	S3	-	-
**(24)**	**1124908**	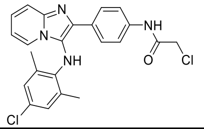	S2	S3	-	-
**(25)**	**1124909**	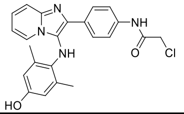	S2	S3	-	S6
**(26)**	**1132336**	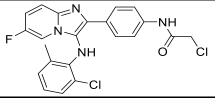	S2	S3	-	-
**(27)**	**1132335**	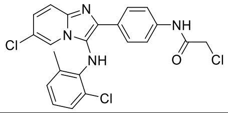	S2	S3	-	-

**(28)**	**1132334**	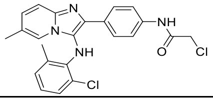	S2	S3	-	S6
**(29)**	**1132332**	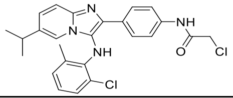	S2	S3	-	-
**(30)**	**1132333**	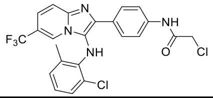	S2	S3	-	-
**(31)**	**1132337**	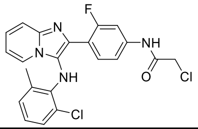	S2	S3	-	-
**(32)**	**1132338**	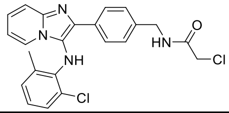	S2	S3	-	S6
**(33)**	**1132339**	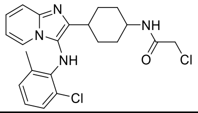	S2	S3	-	S6
**(34)**	**1132341**	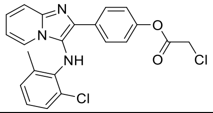	S2	S3	-	-
**(35)**	**1132340**	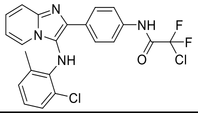	S2	S3	-	-
**(36)**	**1132342**	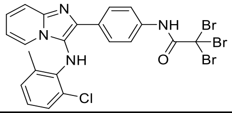	S2	S3	-	-
**(37)**	**1132343**	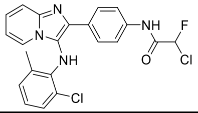	S2	S3	-	-
**(38)**	**1133566**	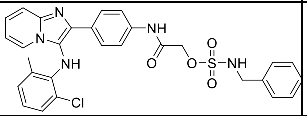	S2	S3	-	-
**(39)**	**1132364**	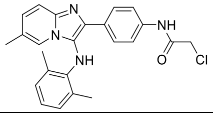	S2	S3	-	-
**(40)**	**1132365**	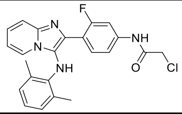	S2	S3	S5	S6
**(41)**	**1132366**	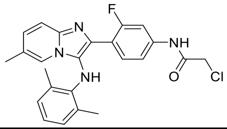	S2	S3	S5	S6
**(42)**	**1132367**	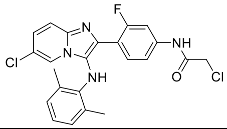	S2	S3	S5	S6

Supplementary Table 2. Percentage (%) bound of compound (1 μM) to 14-3-3σ (100 nM) measured by mass spectrometry in the absence of peptide (apo) or with ERα peptide (2 μM) after 1, 8, 16 and 24 hours.

		APO				ERα			
**No.**	**SMDC ID**	**1h**	**8h**	**16h**	**24h**	**1h**	**8h**	**16h**	**24h**
**(1)**	**1076388**	2	35.7	40.1	58.5	31.5	55.7	64.9	73.2
**(2)**	**1083860**	2	4.8	9.1	13.4	3.8	11.5	17.3	29
**(3)**	**1083861**	2	4.8	7.4	13.4	5.2	7.3	15.4	23.8
**(4)**	**1083857**	1.4	3.3	10.2	12.1	2.4	11.8	16.3	31.9
**(5)**	**1084657**	4.3	9.9	20.5	27.6	10	26.3	45.3	62.9
**(6)**	**1083858**	1	3.9	4.3	9.1	1	3.8	6.9	20.9
**(7)**	**1083859**	1	2	3.4	4.7	1	2	4.7	7.4
**(8)**	**1084659**	9.5	20	29	44.2	14.1	22	31.7	45.9
**(9)**	**1083866**	0	0.5	1.5	2.4	1.5	1.5	2.4	3.8
**(10)**	**1124905**	30.5	56.3	78.8	94	43.6	68.9	92.6	96.6
**(11)**	**1083865**	1.5	3.4	5.2	7.4	11.4	24.3	44.5	78.5
**(12)**	**1084656**	10.2	16.4	28.3	38.7	12.4	31.4	59.2	73.2
**(13)**	**1124899**	0	1.4	1.5	2.9	24.9	42.1	74.2	85.8
**(14)**	**1084652**	1.9	2.9	5.2	7.4	6.5	17.6	23.3	30.7
**(15)**	**1124900**	3.8	3.8	4.3	4.3	4.8	13.4	17.1	23.4
**(16)**	**1124902**	0.5	1.9	2.4	14.4	12.2	18.6	37.2	62.3
**(17)**	**1084662**	1.5	2.4	3.4	6.1	66.8	88.5	98.5	99
**(18)**	**1124901**	0	0	3.8	5.2	23	42.5	66	81.9
**(19)**	**1124904**	3.4	5.2	13.4	27.4	56.3	83.2	94.3	96.5
**(20)**	**1124906**	2.9	5.2	7	14.5	65.9	84.4	97.2	97.5
**(21)**	**1124907**	3.4	5.2	5.2	12.7	62.3	85.2	93.9	98.2
**(22)**	**1084654**	3.8	6.5	9.1	22.8	21	43.2	71.7	92.5
**(23)**	**1124903**	0.5	2.4	2.4	5.6	10.5	16.2	33.9	34.9
**(24)**	**1124908**	5.2	5.6	5.6	6.1	28.1	45.2	60.6	74.5
**(25)**	**1124909**	5.2	5.2	9.1	9.1	7.1	10.5	14.7	19.6
**(26)**	**1132336**	0	0	0	0	10.4	31	54.9	76.9
**(27)**	**1132335**	0	0.5	2.9	13.4	19.1	42.2	60.2	85.8
**(28)**	**1132334**	0	0	1	5.6	34.7	54.6	83.3	93.3
**(29)**	**1132332**	0	0	0	3.8	3.4	31.5	50.9	79.3
**(30)**	**1132333**	0	0	0	0	1.5	12.1	25.9	45.5
**(31)**	**1132337**	0	0	0	0	6.5	19.1	50	72.1
**(32)**	**1132338**	0	0	2.9	4.3	4.7	12.2	19.2	29.8
**(33)**	**1132339**	0	0	0	1	6.5	21.1	36.2	49.9
**(34)**	**1132341**	0	0	0	0	0	0	0	0
**(35)**	**1132340**	0	0	1.9	6.7	0	0	0	0
**(36)**	**1132342**	0	0	0	3.8	0	0	0	0
**(37)**	**1132343**	0	0	0	0.9	0	0	0	0
**(38)**	**1133566**	0	0.9	0.9	3.4	17.3	37.6	87.9	83.5
**(39)**	**1132364**	0.9	2.4	14.5	23.6	46.1	70.9	85.4	93.2
**(40)**	**1132365**	3.4	6.5	9.9	15.2	41.6	67.7	83.3	87.7
**(41)**	**1132366**	0	3.8	3.8	6.5	47	69	86.3	95.3
**(42)**	**1132367**	1.9	1.9	14.5	20.2	47	72.2	91.2	93.3

Supplementary Table 3. TR-FRET protein titrations data. *App*Kd, Emax, fold-increase and fold-stabilization values refer to measurements after 2h-incubation at room temperature. Fold increase = Emax/Emin. Fold stabilization = *App*Kd$_{(DMSO)}$/*App*Kd$_{(Compound)}$. NT = not tested in this experiment, NA = not applicable.

No.	SMDC ID	*App*Kd compound (nM)	EMax	Fold increase	Fold stabilization
**(1)**	**1076388**	NA	-	-	-
**(10)**	**1124905**	NA	-	-	-
**(11)**	**1083865**	NA	-	-	-
**(12)**	**1084656**	NA	-	-	-
**(13)**	**1124899**	8.1	887.8	2.36	4.19
**(14)**	**1084652**	NA	-	-	-
**(15)**	**1124900**	NA	-	-	-
**(16)**	**1124902**	NA	-	-	-
**(17)**	**1084662**	7.1	991.6	2.58	4.78
**(18)**	**1124901**	11.1	742.8	2.14	3.06
**(19)**	**1124904**	7.7	818.7	2.1	4.41
**(20)**	**1124906**	7.1	894.0	2.47	4.78
**(21)**	**1124907**	6.5	772.7	2.28	5.23
**(22)**	**1084654**	NA	-	-	-
**(23)**	**1124903**	NA	-	-	-
**(24)**	**1124908**	16.7	628.6	1.85	2.03
**(25)**	**1124909**	NA	-	-	-
**(26)**	**1132336**	13.4	757.7	2.1	2.53
**(27)**	**1132335**	8.4	761.3	2.1	4.04
**(28)**	**1132334**	10	627.7	1.73	3.40
**(29)**	**1132332**	NA	-	-	-
**(30)**	**1132333**	NA	-	-	-
**(31)**	**1132337**	9.7	753.5	2.13	3.50
**(32)**	**1132338**	8.6	810	2	3.95
**(33)**	**1132339**	12.1	855.8	2.11	2.80
**(34)**	**1132341**	NA	-	-	-
**(35)**	**1132340**	NA	-	-	-
**(36)**	**1132342**	NA	-	-	-
**(37)**	**1132343**	NA	-	-	-
**(38)**	**1133566**	NA	-	-	-
**(39)**	**1132364**	7.8	1017.2	2.61	4.35
**(40)**	**1132365**	8.4	1184.8	3.12	4.04
**(41)**	**1132366**	5.2	1359.3	3.71	6.53
**(42)**	**1132367**	8.8	1036.7	2.97	3.87
**(181)**	**1083744**	8.6	1725.3	5.52	3.95
**-**	**FC-A**	11	1715.2	5.52	3.09
**-**	**DMSO**	34	-	-	-

Supplementary Table 4. SPR data. Fitted parameters of 1:1 binding model, with ka (association constant), kd (dissociation constant), KD (affinity constant), the standard error (SE), and the maximal signal (Rmax). Other parameters (tc, Chi^2, U-value) indicate the quality of the fit.

		**ERα binary**			**ERα + 181**		
		Replicate 1	Replicate 2	Average ± SD	Replicate 1	Replicate 2	Average ± SD
Kinetic fit	ka (1/Ms)	2.63E+05	2.50E+05	2.6E+05 ± 9.3E+03	3.41E+06	4.96E+06	4.2E+06± 1.1E+06
	SE (ka)	2.70E+04	1.40E+04	-	6.40E+04	6.30E+04	-
	kd (1/s)	0.309	0.274	0.292 ± 0.025	0.0163	0.0147	0.016 ± 0.001
	SE (kd)	0.013	0.005	-	0.0003	0.0002	-
	KD (M)	1.18E-06	1.10E-06	1.1E-06± 5.6E-08	4.79E-09	2.97E-09	3.9E-09 ± 1.3E-09
	Rmax (RU)	20	27	-	21	23	-
	tc	1.46E+12	6.65E+14	-	1.28E+7	1.14E+7	-
	Chi² (RU²)	1.76	1.04	-	0.323	0.176	-
	U-value	15	9	-	4	2	-
Affinity fit	KD (M)	1.61E-06	1.42E-06	1.51E-06± 1.33E-07	-	-	-
	Rmax (RU)	18.6	26.7	-	-	-	-
		**ERα + 17**			**ERα + 41**		
		Replicate 1	Replicate 2	Average ± SD	Replicate 1	Replicate 2	Average ± SD
Kinetic fit	ka (1/Ms)	2.03E+06	2.12E+06	2.1E+06± 6.5E+04	2.24E+06	1.97E+06	2.1E+06± 1.9E+05
	SE (ka)	3.40E+04	3.50E+04	-	3.10E+04	2.00E+04	-
	kd (1/s)	0.0339	0.0301	0.032 ± 0.003	0.0214	0.0211	0.021± 0.000
	SE (kd)	0.0003	0.0003	-	0.0002	0.0001	-
	KD (M)	1.67E-08	1.42E-08	1.5E-08± 1.8E-09	9.55E-09	1.07-08	1.0E-08 ± 8.3E-10
	Rmax (RU)	17	17	-	19	20	-
	tc	1.58E+13	8.81+11	-	8.80E+09	3.65E+09	-
	Chi² (RU²)	0.228	0.346	-	0.353	0.265	-
	U-value	4	4	-	3	2	-
Affinity fit	KD (M)	2.42E-08	1.91E-08	2.2E-08± 3.6E-09	9.64E-09	9.37E-09	9.5E-09 ± 1.9E-10
	Rmax (RU)	18	18	-	20	20	-

Supplementary Table 5. 14-3-3σ-HaloTag/NanoLuc-ERα NanoBRET data. Calculated EC_{50} values from compound titrations (2-fold dilution series, starting at 40 μM), pEC_{50} values, and the maximum fold-increase in BRET signal for each compound. Average EC_{50} values from 3 biological replicates.

No.	SMDC ID	EC_{50} (μM)	pEC_{50}	Fold Increase
**(181)**	**1083744**	5.19	5.3	1.66
**(10)**	**1124905**	383	3.4	1.59
**(13)**	**1124899**	11.7	4.9	1.78
**(17)**	**1084662**	2.69	5.6	1.84
**(40)**	**1132365**	7.21	5.1	1.96
**(41)**	**1132366**	4.97	5.3	2.63
**(42)**	**1132367**	4.56	5.3	2.01

Supplementary Table 6: Crystallography

**PDB**	**9I6Y**	**9I6Z**	**9I72**
Protein	14-3-3σΔC	14-3-3σΔC	14-3-3σΔC
Peptide	ERα pT594	ERα pT594	ERα pT594
Compound	**1 (1076388)**	**2 (1083860)**	**10 (1124905)**
Beam	ESRF ID23-1	DESY PETRA III	ESRF ID30A-3
Data collection			
Wavelength (Å)	0.77490	1.033200	0.967697
Space group	C 2 2 21	C 2 2 21	C 2 2 21
Cell dimensions<br>a, b, c (Å)<br>α, β, γ (°)	 82.06, 112.31, 62.62<br>90, 90, 90	 82.14, 112.45, 62.65<br>90, 90, 90	 81.96, 112.46, 62.48<br>90, 90, 90
Resolution (Å)	45.51 – 1.50 (1.53 – 1.50)	45.55 – 1.40 (1.42 – 1.40)	62.48 – 1.35 (1.37 – 1.35)
I / σ(I)	12.0 (3.7)	33.3 (10.3)	20.7 (2.2)
Completeness (%)	100.0 (99.9)	99.5 (98.3)	99.4 (94.7)
Redundancy	12.3 (13.4)	11.4 (9.1)	13.3 (8.8)
$CC_{1/2}$	0.999 (0.940)	0.999 (0.981)	1.000 (0.763)
Refinement			
No. reflections	46592	56783	63101
R_{work}/R_{free}	0.164/0.187	0.150/0.162	0.149/0.172
No. atoms<br>Protein<br>Ligand/ion<br>Water	 1957<br>33<br>314	 1985<br>35<br>324	 1944<br>32<br>314
B-factors<br>Protein<br>Ligand/ion<br>Water	 15.03<br>22.03<br>26.13	 14.25<br>21.53<br>28.48	 16.21<br>26.84<br>30.43
Occupancy ligand	1.00	1.00	1.00
R.m.s. deviations<br>Bond lengths (Å)<br>Bond angles (°)	 0.185<br>4.62	 0.009<br>0.99	 0.012<br>1.13
Ramachandran<br>favored (%)<br>outliers (%)	 98.72<br>0.00	 98.72<br>0.00	 98.72<br>0.00

**PDB**	**9I70**	**9I71**	**9I73**
Protein	14-3-3σΔC	14-3-3σΔC	14-3-3σΔC
Peptide	ERα pT594	ERα pT594	ERα pT594
Compound	**17 (1084662)**	**19 (1124904)**	**20 (1124906)**
Beam	ESRF ID23-1	ESRF ID30A-3	ESRF ID30A-3
Data collection			
Wavelength (Å)	0.885603	0.967697	0.967697
Space group	C 2 2 21	C 2 2 21	C 2 2 21
Cell dimensions<br>a, b, c (Å)<br>α, β, γ (°)	 82.27, 112.49, 62.65<br>90, 90, 90	 82.56, 112.85, 62.57<br>90, 90, 90	 82.36, 112.61, 62.75<br>90, 90, 90
Resolution (Å)	56.24 – 1.40 (1.42 – 1.40)	62.57 – 1.35 (1.37 – 1.35)	41.91 – 1.40 (1.43 – 1.40)
I / σ(I)	18.1 (8.0)	25.2 (3.5)	21.6 (4.4)
Completeness (%)	97.0 (98.9)	99.8 (98.5)	99.9 (99.8)
Redundancy	11.3 (10.6)	13.3 (8.4)	13.8 (13.0)
$CC_{1/2}$	0.996 (0.961)	0.999 (0.897)	0.999 (0.924)
Refinement			
No. reflections	55463	64155	57558
R_{work}/R_{free}	0.136/0.158	0.144/0.176	0.140/0.160
No. atoms<br>Protein<br>Ligand/ion<br>Water	 1957<br>33<br>301	 1983<br>34<br>355	 1982<br>32<br>343
B-factors<br>Protein<br>Ligand/ion<br>Water	 18.19<br>42.12<br>31.29	 18.88<br>20.72<br>31.47	 14.44<br>20.92<br>29.14
Occupancy ligand	0.81	1.00	1.00
R.m.s. deviations<br>Bond lengths (Å)<br>Bond angles (°)	 0.384<br>5.34	 0.014<br>1.24	 0.013<br>1.20
Ramachandran<br>favored (%)<br>outliers (%)	 98.30<br>0.00	 98.30<br>0.00	 98.72<br>0.00

**PDB**	**9I74**	**9I75**	**9I6S**
Protein	14-3-3σΔC	14-3-3σΔC	14-3-3σΔC
Peptide	ERα pT594	ERα pT594	ERα pT594
Compound	**21 (1124907)**	**25 (1124909)**	**28 (1132334)**
Beam	ESRF ID30A-3	ESRF ID30A-3	ESRF ID23-2
Data collection			
Wavelength (Å)	0.967697	0.967697	0.873128
Space group	C 2 2 21	C 2 2 21	C 2 2 21
Cell dimensions<br>a, b, c (Å)<br>α, β, γ (°)	 82.18, 112.16, 62.51<br>90, 90, 90	 82.40, 112.44, 62.59<br>90, 90, 90	 82.96, 113.10, 62.97<br>90, 90, 90
Resolution (Å)	56.08 – 1.50 (1.53 – 1.50)	56.22 – 1.40 (1.42 – 1.40)	66.89 – 1.30 (1.32 - 1.30)
$I / \sigma(I)$	21.3 (5.4)	26.2 (6.0)	16.8 (2.1)
Completeness (%)	99.9 (99.9)	99.5 (97.8)	100.0 (99.8)
Redundancy	13.8 (14.1)	13.8 (13.4)	11.3 (11.1)
$CC_{1/2}$	0.999 (0.958)	0.999 (0.962)	0.999 (0.726)
Refinement			
No. reflections	46639	57133	72001
R_{work}/R_{free}	0.143/0.166	0.138/0.160	0.164/0.192
No. atoms<br>Protein<br>Ligand/ion<br>Water	 1992<br>32<br>300	 1979<br>36<br>342	 1930<br>32<br>279
B-factors<br>Protein<br>Ligand/ion<br>Water	 16.64<br>36.28<br>30.18	 14.85<br>33.22<br>29.44	 19.83<br>28.23<br>31.75
Occupancy ligand	1.00	1.00	1.00
R.m.s. deviations<br>Bond lengths (Å)<br>Bond angles (°)	 0.017<br>1.37	 0.011<br>1.08	 0.019<br>1.44
Ramachandran<br>favored (%)<br>outliers (%)	 98.72<br>0.00	 98.72<br>0.00	 98.30<br>0.00

**PDB**	**9I6T**	**9I6U**
Protein	14-3-3σΔC	14-3-3σΔC
Peptide	ERα pT594	ERα pT594
Compound	**32 (1132338)**	**33 (1132339)**
Beam	ESRF ID23-2	ESRF ID23-2
Data collection		
Wavelength (Å)	0.873128	0.873128
Space group	C 2 2 21	C 2 2 21
Cell dimensions<br>a, b, c (Å)<br>α, β, γ (°)	 82.19, 113.03, 62.89<br>90, 90, 90	 82.78, 113.12, 62.98<br>90, 90, 90
Resolution (Å)	62.89 – 1.30 (1.32 – 1.30)	62.98 – 1.30 (1.32 – 1.30)
$I / \sigma(I)$	21.7 (3.7)	19.7 (3.0)
Completeness (%)	99.9 (100.0)	100.0 (100.0)
Redundancy	11.8 (11.6)	12.3 (12.1)
$CC_{1/2}$	1.000 (0.881)	0.999 (0.839)
Refinement		
No. reflections	72033	72612
R_{work}/R_{free}	0.145/0.171	0.147/0.175
No. atoms<br>Protein<br>Ligand/ion<br>Water	 1986<br>33<br>310	 2006<br>32<br>285
B-factors<br>Protein<br>Ligand/ion<br>Water	 27.30<br>21.26<br>31.41	 19.45<br>33.17<br>31.63
Occupancy ligand	1.00	1.00
R.m.s. deviations<br>Bond lengths (Å)<br>Bond angles (°)	 0.011<br>1.13	 0.013<br>1.14
Ramachandran<br>favored (%)<br>outliers (%)	 98.30<br>0.00	 98.72<br>1.28

PDB	9I6V	9I6W	9I6X
Protein	14-3-3σΔC	14-3-3σΔC	14-3-3σΔC
Peptide	ERα pT594	ERα pT594	ERα pT594
Compound	**40 (1132365)**	**41 (1132366)**	**42 (1132367)**
Beam	ESRF ID23-2	ESRF ID23-2	ESRF ID23-2
Data collection			
Wavelength (Å)	0.873128	0.873128	0.873128
Space group	C 2 2 21	C 2 2 21	C 2 2 21
Cell dimensions<br>a, b, c (Å)<br>α, β, γ (°)	 82.99, 113.07, 62.90<br>90, 90, 90	 82.88, 113.01, 62.92<br>90, 90, 90	 82.89, 112.98, 62.86<br>90, 90, 90
Resolution (Å)	66.90 – 1.30 (1.32 – 1.30)	45.81 – 1.30 (1.32 – 1.30)	45.79 – 1.30 (1.32 – 1.30)
$I / \sigma(I)$	19.9 (3.5)	13.9 (1.3)	15.3 (1.6)
Completeness (%)	86.0 (84.5)	99.7 (99.4)	99.6 (99.1)
Redundancy	12.4 (11.2)	12.5 (12.5)	10.9 (10.7)
$CC_{1/2}$	0.999 (0.842)	0.999 (0.525)	0.999 (0.599)
Refinement			
No. reflections	62491	72395	72326
R_{work}/R_{free}	0.145/0.175	0.153/0.184	0.149/0.174
No. atoms<br>Protein<br>Ligand/ion<br>Water	 1984<br>34<br>283	 1973<br>35<br>297	 1967<br>35<br>290
B-factors<br>Protein<br>Ligand/ion<br>Water	 18.14<br>47.55<br>29.66	 21.75<br>40.95<br>33.32	 21.46<br>30.11<br>33.24
Occupancy ligand	1.00	1.00	1.00
R.m.s. deviations<br>Bond lengths (Å)<br>Bond angles (°)	 0.014<br>1.15	 0.014<br>1.24	 0.016<br>1.40
Ramachandran<br>favored (%)<br>outliers (%)	 98.72<br>0.00	 98.72<br>0.00	 98.30<br>0.00

3. Supplementary Methods

Synthetic procedures

General remarks

All the reagents and solvents were purchased from Sigma-Aldrich, AK Scientific, Fluorochem, Abcr GmbH, Acros, AA blocks, Ambeed, 1PlusChem, Combi-blocks, ChemScene, Enamine and were used without further purification. Thin layer chromatography was performed on Millipore precoated silica gel plates (0.20 mm thick, particle size 25 µm). Nuclear magnetic resonance spectra were recorded on a Bruker Avance 500 spectrometer {^{1}H NMR (500 MHz), ^{13}C NMR (125 MHz)} or on a Bruker Advance-III 400 MHz equipped with a BBFO probe from Bruker {^{1}H NMR (400 MHz), ^{13}C NMR (100 MHz)}. Chemical shifts for ^{1}H-NMR were reported as δ values and coupling constants were in hertz (Hz). The following abbreviations were used for spin multiplicity: s = singlet, d = doublet, t = triplet, q = quartet, quin = quintet, dd = double of doublets, dt = doublet of triplets, td = triplet of doublets, m = multiplet. Chemical shifts for ^{13}C-NMR were reported in ppm relative to the solvent peak. High resolution mass spectra were recorded using a LTQ-Orbitrap-XL (Thermo) at a resolution of 60000@m/z400.

Synthetic routes

Scheme 1

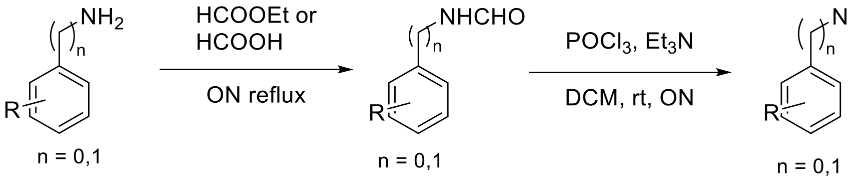

Step 1: In a round bottom flask, the appropriate benzylamine or aniline (1.0 equiv.) was dissolved in ethyl formate or formic acid (6.0 equiv.). Formic acid was used for *ortho*-substituted anilines. The reaction mixture was refluxed overnight. The solvent was removed under reduced pressure. *Step 2:* The intermediate formamide was dissolved in DCM (0.33 M). Triethylamine (5.0 equiv.) was added, and the reaction mixture was cooled at 0°C. $POCl_3$ (1.3 equiv.) was added dropwise over 30 min. The reaction mixture was stirred overnight at room temperature under a $CaCl_2$ tube. The reaction mixture was quenched at 0°C with saturated $NaHCO_3$, and was stirred vigorously for 2h. The mixture was filtered under vacuum and the filtrate was extracted with DCM (x3). The combined organic layers were collected, dried with sodium sulfate, filtered and concentrated under reduced pressure. The crude was purified by filtration over silica under vacuum with ethyl acetate as the eluent. The solvent was removed under reduced pressure. The isocyanides were isolated with yields >80%.

Scheme 2

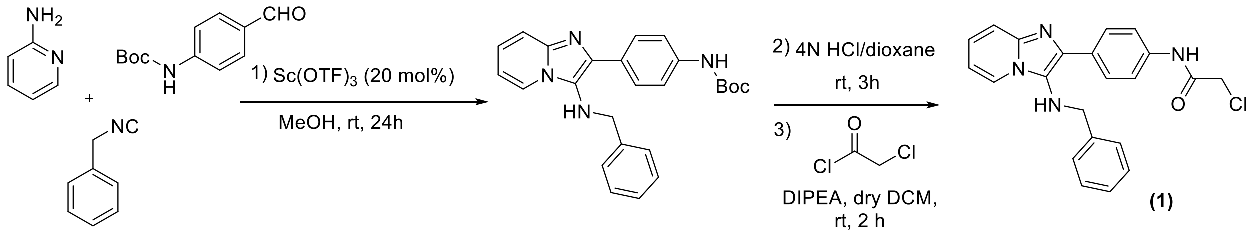

(1)

Step 1: In a vial, *tert*-butyl N-(4-formylphenyl)carbamate (0.5 mmol, 1.0 equiv.) and 2-aminopyridine (0.5 mmol, 1.0 equiv) were dissolved in MeOH (0.5M). Scandium triflate (20 mol%) and benzyl isocyanide (0.5 mmol, 1.0 equiv) were added sequentially under stirring. The reaction mixture was stirred at rt overnight. The solvent was removed under reduced pressure and the residue was purified with flash column chromatography [hexane – EtOAc, 0-100% EtOAc in hexane]. The obtained yellow solid was used directly in the next step. *Step 2*: The residue was suspended in 3 ml HCl/dioxane (4N) for Boc-deprotection. Stirring at rt for 3h. The solvent was removed under reduced pressure and the obtained oil was used directly in the next step. *Step 3*: the obtained HCl salt (0.34 mmol, 1.0 equiv.) was suspended in 4 ml dry DCM and cooled at 0 °C. Under stirring, DIPEA (1.4 mmol, 4 equiv.) was added. After 10 min, chloroacetyl chloride (0.52 mmol, 1.5 equiv.) was added slowly. Stirring at rt for 2h. The reaction mixture was diluted with DCM (10 ml), quenched with sat. $NaHCO_3$ (10 ml) and extracted with DCM (3 x 10 ml). The combined organic phases were dried over $MgSO_4$, filtered and concentrated under reduced pressure. The obtained crude was purified with flash column chromatography [hexane – EA, 0-100% EtOAc in hexane].

Scheme 3

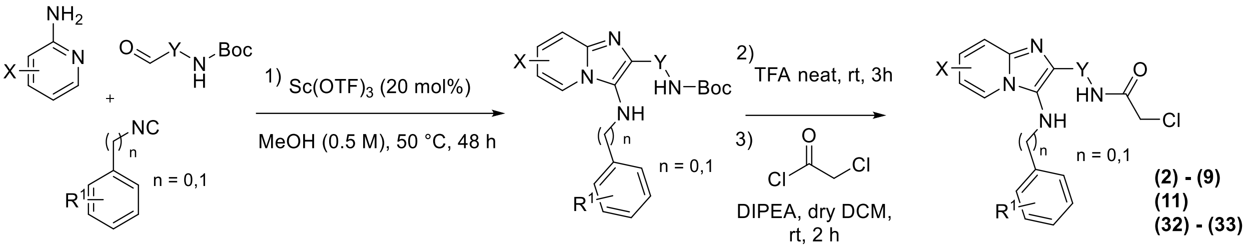

Step 1: To a stirred solution of the appropriate Boc-protected aldehyde (1.2 mmol, 1.0 equiv.) in MeOH (2.4 ml, 0.5M), the appropriate 2-amino pyridine (1.2 mmol, 1.0 equiv.) was added at room temperature. Then, scandium triflate was added (20 mol%) followed by the corresponding isocyanide (1.2 mmol, 1.0 equiv.) and the reaction mixture was stirred vigorously at 50 °C for 48 h. The solvent was removed under reduced pressure and the reaction mixture was diluted with ethyl acetate and washed with water (x3). The organic layer was collected, dried with sodium sulfate, filtered and concentrated under reduced pressure. The crude product was used directly in the next step. *Step 2*: To a stirred solution of the intermediate (1.0-1.2 mmol, 1 equiv.), TFA (10.0-12.0 mmol, 10.0 equiv.) was added at room temperature. The reaction mixture was stirred vigorously for 3 h. TFA was removed under reduced pressure, and the TFA salt was used directly in the next step. *Step 3:* To a stirred solution of the TFA salt (1.0-1.2 mmol, 1.0 equiv) in dry DCM (0.08 M), DIPEA (4.0-4.8 mmol, 4.0 equiv.) was added at 0 °C. The reaction mixture was stirred for 10 min. Then, chloroacetyl chloride was added dropwise (1.5-1.8 mmol, 1.5 equiv.) and the reaction mixture was stirred vigorously at room temperature for 2 h. The reaction mixture was diluted with DCM (10 ml), quenched with sat. $NaHCO_3$ (10 ml) and extracted with DCM (3 x 10 ml). The combined organic layers were collected, dried with sodium sulfate, filtered and concentrated under reduced pressure. The crude was purified with column chromatography (PE-EtOAc 2:1-1:2) to yield compounds **(2)-(9), (11), (32)** and **(33)**.

Scheme 4

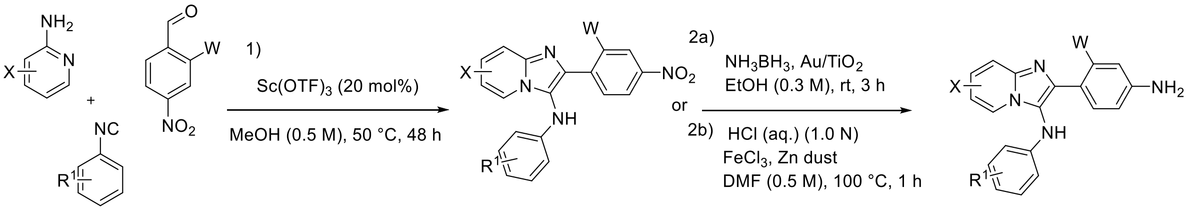

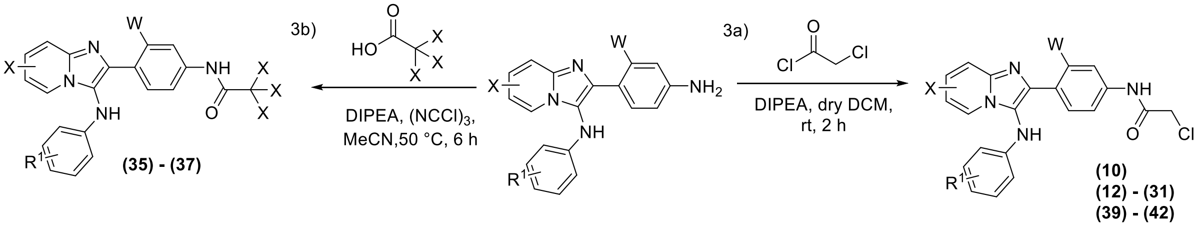

Step 1: To a stirred solution of the appropriate 4-nitrobenzaldehydes (1.2 mmol, 1.0 equiv.) in MeOH (2.4 ml), the appropriate 2-amino pyridine (1.2 mmol, 1.0 equiv.) was added at room temperature. Then, scandium triflate was added (20 mol%) followed by the corresponding isocyanide (1.2 mmol, 1.0 equiv.). The reaction mixture was stirred vigorously at 50 °C for 48 h. The solvent was removed under reduced pressure and the reaction mixture was diluted with ethyl acetate and washed with water (x3). The organic layer was collected, dried with sodium sulfate, filtered and the solvent was removed under reduced pressure. The crude product was used directly in the next step. Two methods were used for the reduction of the nitro group, leading to quantitative yields. *Step 2*, *method A*: To a stirred solution of the intermediate (1.0-1.2 mmol, 1.0 equiv.) in EtOH (0.3 M), ammoniotrihydroborate (2.5-3.0 mmol, 2.5 equiv.) was added, followed by Au/TiO_2 (2.0-2.4 mmol, 2.0 equiv.). The reaction mixture was stirred vigorously for 3 h at rt. The reaction mixture was filtrated over celite, and the solvent was removed under reduced pressure. This method was used for compounds **(10), (13), (15), (16), (17), (18), (19)** and **(23).** *Step 2*, *method B*: To a stirred solution of the intermediate (1.0-1.2 mmol, 1.0 equiv.) in DMF (0.5 M), HCl (1.0 N, 2.0-2.4 mmol, 2 equiv.) was added followed by iron trichloride (3.0-3.6 mmol, 3.0 equiv.) and Zn dust (10.0-12.0 mmol, 10 equiv.). The reaction mixture was stirred vigorously at 100

°C for 1 h. The reaction mixture was filtered over celite, and the filtrate was washed with water and sat. $NaHCO_3$. The organic layer was collected, dried with sodium sulfate, filtered and the solvent was removed under reduced pressure. The crude product was used directly in the next step. This method was all remaining compounds. *Step 3, method A*: To a stirred solution of the intermediate (1.0-1.2 mmol, 1.0 equiv) in dry DCM (0.08 M), DIPEA (4.0-4.8 mmol, 4.0 equiv.) was added at 0 °C. The reaction mixture was stirred for 10 min. Then, chloroacetyl chloride was added (1.5-1.8 mmol, 1.5 equiv.) dropwise and the reaction mixture was stirred vigorously at room temperature for 2 h. The reaction mixture was diluted with DCM (10 ml), quenched with sat. $NaHCO_3$ (10 ml) and extracted with DCM (3 x 10ml). The combine organic layers were collected, dried with sodium sulfate and filtered. The solvent was removed under reduced pressure. The crude was purified with column chromatography (PE-EtOAc 2:1-1:2) to yield compounds **(10), (12)-(31), (39)-(42)**. *Step 3, method B*: To a stirred solution of the intermediate (1.0-1.2 mmol, 1.0 equiv.) in MeCN (0.1 M), DIPEA was added (1.2 equiv.), followed by the appropriate carboxylic acid (1.0 equiv.) and cyanuric chloride (0.6 equiv.) The reaction mixture was stirred vigorously at 50 °C for 6 h. Solvent was removed under reduced pressure. The crude was purified with column chromatography (PE-EtOAc 2:1-1:2) to yield compounds **(35)-(37)**.

Scheme 5

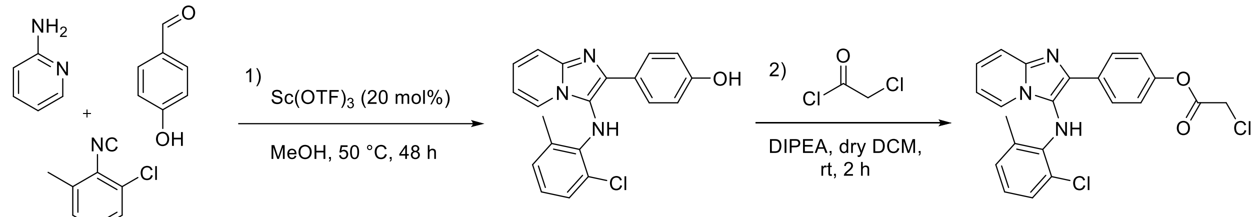

(34)

Step 1: To a stirred solution of 4-hydroxybenzaldehyde (1.2 mmol, 1.0 equiv.) in MeOH (2.4 ml), 2-amino pyridine (1.2 mmol, 1.0 equiv.) was added at room temperature. Then, scandium triflate was added (20 mol%) followed by the isocyanide (1.2 mmol, 1.0 equiv.). The reaction mixture was stirred vigorously at 50°C for 48 h. The solvent was removed under reduced pressure and the reaction mixture was diluted with ethyl acetate and washed with water (x3). The organic layer was collected, dried with sodium sulfate, filtered and the solvent was removed under reduced pressure. The crude product was used directly in the next step. *Step 2*: To a stirred solution of the intermediate (1.0 equiv.) in dry DCM (0.08 M), DIPEA (4.0 equiv.) was added at 0 °C. The reaction mixture was stirred for 10 min. Then, chloroacetyl chloride was added dropwise (1.5 equiv.) and the reaction mixture was stirred vigorously at room temperature for 2 h. The reaction mixture was diluted with DCM (10 ml), quenched with sat. $NaHCO_3$ (10 ml) and extracted with DCM (3 x 10 ml). The combined organic layers were collected, dried with sodium sulfate, filtered and concentrated under reduced pressure. The crude was purified with column chromatography (PE-EtOAc 2:1-1:2) to yield compound **(34)**.

Scheme 6

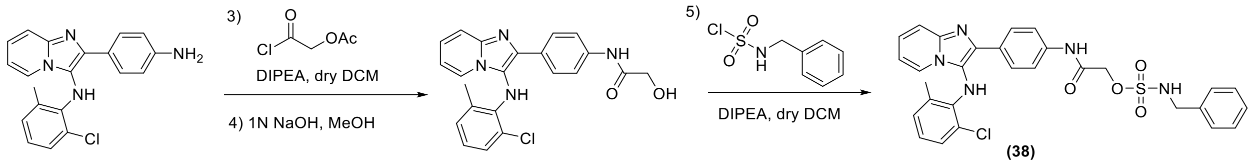

(38)

The main intermediate was synthesized following steps 1 and 2 descripted in scheme 4. *Step 3:* To a stirred solution of the intermediate (0.4 mmol, 1.0 equiv.) in dry DCM (0.08 M), DIPEA (4.0 equiv.) was added at 0 °C. The reaction mixture was stirred for 10 min. Then, acetoxyacetyl chloride was added slowly (1.0 equiv.) and the reaction mixture was stirred at room temperature for 2 h. The reaction mixture was diluted with DCM (10 ml), quenched with sat. $NaHCO_3$ (10 ml) and extracted with DCM (3 x 10 ml). The combined organic layers were collected, dried with sodium sulfate, filtered and concentrated under reduced pressure. *Step 4:* the crude was dissolved in MeOH (2 ml) and NaOH (1N, 1ml) was added to the reaction mixture. The reaction mixture was stirred at rt for 4h. The solvent was removed under reduced pressure and the residue was redissolved in ethyl acetate (10 ml). The organic layer was washed with water (x3). The organic layer was collected, dried with sodium sulfate, filtered and the solvent was removed under reduced pressure. *Step 5:* the crude was dissolved in 3 ml dry DCM and cooled at 0 ^{o}C. Under stirring, DIPEA (4 equiv.) was added. After 10 min, benzylsulfamoyl chloride (1.5 equiv.) was added slowly. Stirring at rt for 2h. The reaction mixture was diluted with DCM (10 ml), quenched with sat. $NaHCO_3$ (10 ml) and extracted with DCM (3 x 10 ml). The combined organic phases were dried over $MgSO_4$, filtered and concentrated under reduced pressure. The obtained crude was purified with flash column chromatography [hexane – EA, 0-100% EtOAc in hexane] to obtain compound **(38)**.

***N*-(4-(3-(benzylamino)imidazo[1,2-a]pyridin-2-yl)phenyl)-2-chloroacetamide (1) 1076388**

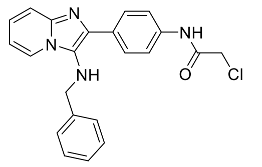

Obtained using scheme 2; 0.5 mmol scale, 71.1 mg, yield 53%, yellow solid. ^{1}H NMR (400 MHz, $CDCl_3$) δ 8.40 (b, 1H), 7.99 (dd, *J* = 10.1, 7.8 Hz, 3H), 7.65 (d, *J* = 8.6 Hz, 2H), 7.54 (d, *J* = 9.0 Hz, 1H), 7.36 – 7.26 (m, 5H), 7.14 (dd, *J* = 11.3, 4.3 Hz, 1H), 6.75 (t, *J* = 6.5 Hz, 1H), 4.21 (s, 2H), 4.19 (d, *J* = 5.1 Hz, 2H), 3.46 (b, 1H). ^{13}C NMR (100 MHz, $CDCl_3$) δ 163.8, 141.6, 138.8, 135.9, 135.4, 131.1, 128.7, 128.2, 127.7, 127.6, 125.5, 124.2, 122.3, 120.1, 117.4, 111.8, 52.4, 42.9. LCMS (ESI): m/z calcd for $C_{22}H_{19}ClN_4O$; found $[M+H]^+$ 391.24.

2-chloro-*N*-(4-(3-((2-fluorobenzyl)amino)imidazo[1,2-a]pyridin-2-yl)phenyl)acetamide (2) 1083860

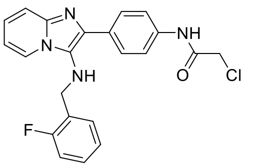

Obtained using scheme 3; 1.2 mmol scale, 236 mg, yield 38%, dark red solid. ^{1}H NMR (500 MHz, $CDCl_3$): 8.48 (s, 1H), 8.01-7.96 (m, 3H), 7.62 (d, *J* = 8.5 Hz, 2H), 7.51 (d, *J* = 9 Hz, 1H), 7.25-7.17 (m, 2H), 7.14-7.10 (m, 1H), 7.05-7.01 (m, 2H), 6.74 (td, *J1* = 6.5 Hz, *J2* = 1 Hz, 1H), 4.21-4.19 (m, 4H); ^{13}C NMR (125 MHz, $CDCl_3$): 163.8, 161.2 (d, $J_{C\text{-}F}$ = 244 Hz), 141.6, 136.0, 135.7, 131.0, 130.5 (d, $J_{C\text{-}F}$ = 4.5 Hz), 129.6 (d, $J_{C\text{-}F}$ = 8 Hz), 127.6, 125.8 (d, $J_{C\text{-}F}$ = 15 Hz), 125.1, 124.3 (d, $J_{C\text{-}F}$ = 3.5 Hz), 124.2, 122.3, 120.1, 117.3, 115.5 (d, $J_{C\text{-}F}$ = 21.5 Hz), 111.8, 46.2 (d, $J_{C\text{-}F}$ = 3 Hz), 42.9; HRMS (ESI) m/z: $[M+H]^+$: $C_{22}H_{19}ClFN_4O$, calculated 409.12259; found 409.12319.

2-chloro-*N*-(4-(3-((3-fluorobenzyl)amino)imidazo[1,2-a]pyridin-2-yl)phenyl)acetamide (3) 1083861

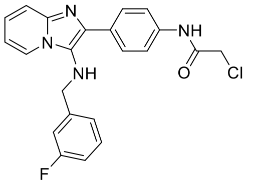

Obtained using scheme 3; 1.2 mmol scale, 152 mg, yield 25%, dark red solid. ^{1}H NMR (500 MHz, $CDCl_3$): 8.34 (s, 1H), 8.00-7.97 (m, 3H), 7.66 (d, *J* = 9 Hz, 2H), 7.55 (dt, J_1 = 9 Hz, J_2= 1 Hz, 1H), 7.29-7.25 (m, 1H), 7.16-7.13 (m, 1H), 7.10-7.07(m, 2H), 6.99-6.95 (m, 1H), 6.77 (td, J_1 = 6.5 Hz, J_2 = 1 Hz, 1H), 4.22 (s, 2H), 4.19 (d, *J* = 5.5 Hz, 2H); ^{13}C NMR (125 MHz, $CDCl_3$): 163.7, 141.7, 141.4 (d, $J_{C\text{-}F}$ = 6.5 Hz), 136.0, 135.8, 131.1, 130.2 (d, $J_{C\text{-}F}$ = 8.5 Hz), 127.7, 125.1, 124.3, 123.7 (d, $J_{C\text{-}F}$ = 3 Hz), 122.2, 120.2, 117.5, 115.0 (d, $J_{C\text{-}F}$ = 21 Hz), 114.7, 114.5, 111.9, 51.9, 42.9; HRMS (ESI) m/z: $[M+H]^+$: $C_{22}H_{19}ClFN_4O$, calculated 409.12259; found 409.12316.

2-chloro-*N*-(4-(3-((4-fluorobenzyl)amino)imidazo[1,2-a]pyridin-2 yl)phenyl)acetamide (4) 1083857

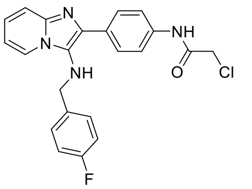

Obtained using scheme 3; 1.2 mmol scale, 291 mg, yield 53%, dark red solid. ^{1}H NMR (500 MHz, $CDCl_3$): 8.54 (s, 1H), 7.97-7.92 (m, 3H), 7.63 (d, *J* = 8 Hz, 2H), 7.54 (d, *J* = 9 Hz, 1H), 7.25-7.22 (m, 2H), 7.17-7.13 (m, 1H), 6.96 (t, *J* = 8.5 Hz, 2H), 6.76 (t, *J* = 6.5 Hz, 1H), 4.21 (s, 2H), 4.12 (s, 2H); ^{13}C NMR (125 MHz, $CDCl_3$): 164.0, 162.3 (d, $J_{C\text{-}F}$ = 245 Hz), 141.3, 136.2, 135.2, 134.6 (d, $J_{C\text{-}F}$ = 3 Hz), 130.4, 129.9 (d, $J_{C\text{-}F}$ = 8 Hz), 127.7, 125.2, 124.8, 122.3, 120.2, 117.0, 115.5 (d, $J_{C\text{-}F}$ = 21 Hz), 112.1, 51.6, 43.0; HRMS (ESI) m/z: $[M+H]^+$: $C_{22}H_{19}ClFN_4O$, calculated 409.12259; found 409.12272.

2-chloro-*N*-(4-(3-((2,6-difluorobenzyl)amino)imidazo[1,2-a]pyridin-2-yl)phenyl)acetamide (5) 1084657

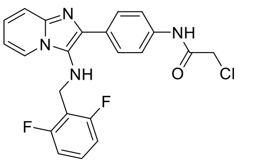

Obtained using scheme 3; 1.2 mmol scale, 160 mg, yield 31%, dark red solid. ^{1}H NMR (500 MHz, $CDCl_3$): 8.43 (s, 1H), 8.11 (d, *J* = 6.5 Hz, 1H), 7.93 (d, *J* = 8.5 Hz, 2H), 7.61 (d, *J* = 8.5 Hz, 2H), 7.57 (d, *J* = 9 Hz, 1H), 7.21-7.17 (m, 2H), 6.85-6.81 (m, 3H), 4.26 (s, 2H), 4.22 (s, 2H). ^{13}C NMR (125 MHz, $CDCl_3$): δ 163.7, 161.5 (dd, $^{1}J_{C\text{-}F}$ = 246 Hz, $^{2}J_{C\text{-}F}$ = 8 Hz), 141.5, 136.1, 135.5, 129.8 (t, $J_{C\text{-}F}$ = 10.5 Hz), 127.6, 127.0, 124.7, 122.4, 120.1, 117.2, 114.4 (t, $J_{C\text{-}F}$ = 20 Hz), 112.2, 111.3 (dd, $^{1}J_{C\text{-}F}$ = 20 Hz, $^{2}J_{C\text{-}F}$ = 5.5 Hz), 42.9, 39.1; HRMS (ESI) m/z: $[M+H]^+$: $C_{22}H_{18}ClF_2N_4O$, calculated 427.11317; found 427.11277.

2-chloro-*N*-(4-(3-((2-chlorobenzyl)amino)imidazo[1,2-a]pyridin-2-yl)phenyl)acetamide (6) 1083858

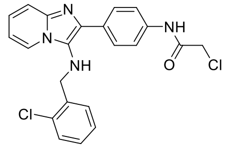

Obtained using scheme 3; 1.2 mmol scale, 234 mg, yield 45%, dark green solid. ^{1}H NMR (500 MHz, $CDCl_3$): 8.38 (s, 1H), 8.00 (dt, J_1 = 7 Hz, J_2 = 1.25 Hz, 1H), 7.92 (d, *J* = 8 Hz, 2H), 7.62-7.59 (m, 3H), 7.33 (dd, J_1 = 8 Hz, J_2 = 1.5 Hz, 1H), 7.20-7.12 (m, 4H), 6.78 (td, J_1 = 7 Hz, J_2 = 1 Hz, 1H), 4.25 (s, 2H), 4.21 (s, 2H); ^{13}C NMR (125 MHz, $CDCl_3$): 174.8, 163.8, 141.2, 136.2, 136.2, 133.9, 130.5, 130.1, 129.7, 129.3, 127.8, 127.1, 125.0, 122.4, 120.1, 116.9, 112.3, 50.1, 42.9; HRMS (ESI) m/z: $[M+H]^+$: $C_{22}H_{19}Cl_2N_4O$, calculated 425.09304; found 425.09382.

2-chloro-*N*-(4-(3-((3-chlorobenzyl)amino)imidazo[1,2-a]pyridin-2-yl)phenyl)acetamide (7) 1083859

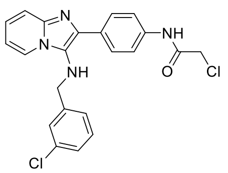

Obtained using scheme 3; 1.2 mmol scale, 397 mg, yield 40%, dark green solid. ^{1}H NMR (500 MHz, $CDCl_3$): 8.42 (s, 1H), 7.98 (d, J = 7 Hz, 1H), 7.94 (d, J = 8.5 Hz, 2H), 7.64 (d, J = 8.5 Hz, 2H), 7.53 (d, J = 9 Hz, 1H), 7.34 (s, 1H), 7.24-7.20 (m, 2H), 7.17-7.14 (m, 2H), 6.78 (td, J_1 = 7 Hz, J_2 = 1 Hz, 1H), 4.20 (s, 2H), 4.14 (s, 2H); ^{13}C NMR (125 MHz, $CDCl_3$): 163.9, 141.3, 140.8, 136.2, 135.4, 134.4, 129.9, 128.2, 127.8, 127.7, 127.1, 126.2, 125.1, 124.7, 122.3, 120.2, 117.1, 112.1, 51.7, 42.9; HRMS (ESI) m/z: $[M+H]^+$: $C_{22}H_{19}Cl_2N_4O$, calculated 425.09304; found 425.09353.

2-chloro-*N*-(4-(3-((4-chlorobenzyl)amino)imidazo[1,2-a]pyridin-2-yl)phenyl)acetamide (8) 1084659

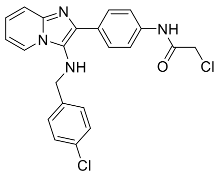

Obtained using scheme 3; 1.2 mmol scale, 197 mg, yield 39%, yellow solid. ^{1}H NMR (500 MHz, $CDCl_3$): 8.77 (s, 1H), 7.99 (d, J = 6.5 Hz, 1H), 7.88 (d, J = 8 Hz, 2H), 7.60 (d, J = 8 Hz, 2H), 7.51 (d, J = 9 Hz, 1H), 7.22-7.16 (m, 5H), 6.79 (t, J = 6.5 Hz), 4.21 (s, 2H), 4.10 (s, 2H); ^{13}C NMR (125 MHz, $CDCl_3$): δ 164.1, 161.1, 140.9, 137.2, 136.5, 133.4, 129.5, 129.1, 128.8, 128.7, 127.7, 125.2, 122.5, 120.1, 116.6, 112.4, 51.5, 43.0. HRMS (ESI) m/z: $[M+H]^+$: $C_{22}H_{19}Cl_2N_4O$, calculated 425.09304; found 425.09297.

2-chloro-*N*-(4-(3-((2-nitrobenzyl)amino)imidazo[1,2-a]pyridin-2-yl)phenyl)acetamide (9) 1083866

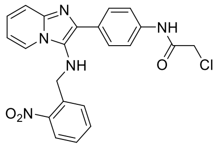

Obtained using scheme 3; 0.5 mmol scale, 87 mg, yield 40%, orange solid. ^{1}H NMR (500 MHz, $CDCl_3$): δ 8.66 (s, 1H), 8.03 (d, J = 6.5 Hz, 1H), 7.88 (d, J = 8 Hz, 1H), 7.76 (d, J = 8 Hz, 2H), 7.53-7.50 (m, 4H), 7.38 (td, J_1 = 7.5 Hz, J_2 = 1.5 Hz, 2H), 7.34 (d, J = 7.5 Hz, 1H), 7.21 (t, J = 7.5 Hz, 1H), 7.10 (dd, J_1 = 7 Hz, J_2 = 1.5 Hz, 1H), 6.84 (t, J = 6.5 Hz, 1H), 4.33 (s, 2H), 4.17 (s, 2H); ^{13}C NMR (125 MHz, $CDCl_3$): δ 164.3, 148.8, 136.7, 133.8, 133.7, 132.1, 129.1, 127.9, 127.2, 125.2, 125.0, 124.8, 122.6, 120.4, 120.2, 118.1, 116.5, 113.1, 49.8, 43.2. LCMS (ESI): m/z: $[M+H]^+$:$C_{22}H_{19}ClN_5O_3$; calculated 436.11; found 436.42.

2-chloro-*N*-(4-(3-(phenylamino)imidazo[1,2-a]pyridin-2-yl)phenyl)acetamide (10) 1124905

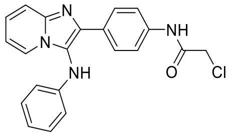

Obtained using scheme 4; 1.2 mmol scale, 189mg, yield 42%, white solid. ^{1}H NMR (500 MHz, $CDCl_3$): 8.27 (s, 1H), 8.01 (d, J = 8.5 Hz, 2H), 7.86 (d, J = 7 Hz, 1H), 7.65 (d, J = 9 Hz, 1H), 7.55 (d, J = 8.5 Hz, 2H), 7.25-7.20 (m, 3H), 6.88 (t, J = 7.5 Hz, 1H), 6.79 (t, J = 7 Hz, 1H), 6.60 (d, J = 8Hz, 2H), 5.64 (s, 1H), 4.18 (s, 2H). ^{13}C NMR (125 MHz, $CDCl_3$): δ 163.7, 144.5, 142.7, 136.8, 130.7, 127.8, 125.5, 123.2, 121.2, 120.1, 120.1, 118.6, 117.6, 114.6, 112.9, 111.9, 43.3. HRMS (ESI) m/z: $[M+H]^+$:$C_{21}H_{18}ClN_4O$, calculated 377.11637; found 377.11656.

2-chloro-*N*-(4-(3-((4-chlorophenyl)amino)imidazo[1,2-a]pyridin-2-yl)phenyl)acetamide (11) 1083865

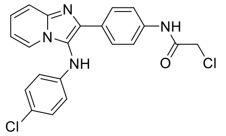

Obtained using scheme 3; 1.2 mmol scale, 225 mg, yield 40%, dark green solid. ^{1}H NMR (500 MHz, $CDCl_3$): 8.52 (s, 1H), 7.84 (d, J = 7 Hz, 2H), 7.64 (d, J = 9 Hz, 1H), 7.43 (d, J = 9 Hz, 2H), 7.28-7.25 (m, 2H), 7.12 (d, J = 9 Hz, 2H), 6.84 (t, J = 7 Hz, 1H), 6.52 (d, J = 9 Hz, 2H), 4.15 (s, 2H); ^{13}C NMR (125 MHz, $CDCl_3$): 164.1, 142.9, 136.9, 129.7, 127.6, 124.8, 122.9, 120.1, 118.1, 116.4, 114.7, 113.4, 43.0; HRMS (ESI) m/z: $[M+H]^+$: $C_{21}H_{17}Cl_2N_4O$, calculated 411.07739; found 411.07805.

***N*-(4-(3-((4-bromophenyl)amino)imidazo[1,2-a]pyridin-2-yl)phenyl)-2-chloroacetamide (12) 1084656**

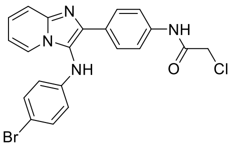

Obtained using scheme 4; 1.2 mmol scale, 96 mg, yield 18%, dark green solid. ^{1}H NMR (500 MHz, MeOD-d_4): 8,33 (d, J = 7 Hz, 1H), 7.89-7.86 (m, 4H), 7.74 (d, J = 9 Hz, 2H), 7.39-7.37 (m, 1H), 7.31 (d, J = 9 Hz, 3H), 6.63 (d, J = 9 Hz, 2H), 4.20 (s, 2H); ^{13}C NMR (125 MHz, MeOD-d_4): δ 167.6, 144.9, 141.0, 140.6, 133.7, 133.5, 128.9, 125.8, 124.7, 121.5, 121.2, 117.6, 116.6, 114.3, 113.1, 112.9, 44.0. HRMS (ESI) m/z: $[M+H]^+$: $C_{21}H_{17}BrClN_4O$, calculated 455.02688; found 455.02668.

2-chloro-*N*-(4-(3-(o-tolylamino)imidazo[1,2-a]pyridin-2-yl)phenyl)acetamide (13) 1124899

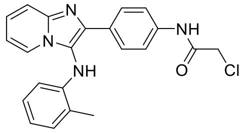

Obtained using scheme 4; 1.2 mmol scale, 105 mg, yield 27%, dark green solid. ^{1}H NMR (500 MHz, $CDCl_3$): 8.29 (s, 1H), 7.96 (d, J = 8.5 Hz, 2H), 7.77 (d, J = 7 Hz, 1H), 7.64 (d, J = 9 Hz, 1H), 7.54 (d, J = 8.5 Hz, 2H), 7.25-7.21 (m, 2Hz), 6.97 (t, J = 7.5 Hz, 1H), 6.81 (dt, J_1 = 7.5 Hz, J_2 = 1 Hz, 1H), 6.78 (t, J = 7.5 Hz, 1H), 6.17 (d, J = 1 Hz, 1H), 5.47 (s, 1H), 4.17 (s, 2H), 2.43 (s, 3H); ^{13}C NMR (125 MHz, $CDCl_3$): δ 163.7, 142.7, 142.3, 138.4, 136.3, 131.0, 130.3, 127.7, 127.6, 125.2, 122.7, 122.4, 120.1, 119.9, 118.2, 117.5, 112.3, 111.8, 42.9, 17.6; HRMS (ESI) m/z: $[M+H]^+$: $C_{22}H_{20}ClN_4O$, calculated 391.13202; found 391.13157.

2-chloro-*N*-(4-(3-((2-ethylphenyl)amino)imidazo[1,2-a]pyridin-2-yl)phenyl)acetamide (14) 1084652

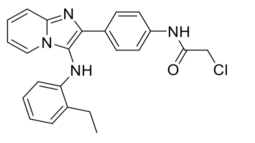

Obtained using scheme 4; 1.2 mmol scale, 160 mg, yield 33%, dark green solid. ^{1}H NMR (500 MHz, $CDCl_3$): 8.43 (s, 1H), 7.92 (d, *J* = 9 Hz, 2H), 7.71 (d, *J* = 6.5 Hz, 1H), 7.60 (d, *J* = 9 Hz, 1H), 7.50 (d, *J* = 8.5 Hz, 2H), 7.24-7.18 (m, 2H), 6.96-6.93 (m, 1H), 6.84 (t, *J* = 8 Hz, 1H), 6.72 (t, *J* = 7 Hz, 1H), 6.13 (d, *J* = 8 Hz, 1H), 5.62 (s, 1H), 4.14 (s, 2H), 2.79 (q, *J* = 7.5 Hz, 2H), 1.41 (t, *J* = 7.5 Hz, 3H), 1.28 (s, 3H); ^{13}C NMR (125 MHz, $CDCl_3$): δ 163.8, 143.2, 141.6, 136.4, 130.2, 129.0, 128.2, 127.6, 127.4, 125.7, 125.1, 122.7, 120.0, 120.0, 118.1, 117.4, 112.2, 111.9, 42.9, 31.5, 24.1, 13.5. HRMS (ESI) m/z: $[M+H]^+$: $C_{23}H_{22}ClN_4O$, calculated 405.14767; found 405.14785.

2-chloro-*N*-(4-(3-((2-(trifluoromethyl)phenyl)amino)imidazo[1,2-a]pyridin-2-yl)phenyl)acetamide (15) 1124900

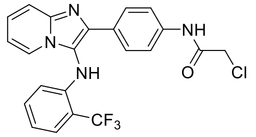

Obtained using scheme 4; 1.2 mmol scale, 85 mg, yield 16%, white solid. ^{1}H NMR (500 MHz, Acetone-d_6): 9.46 (s, 1H), 8.10 (d, *J* = 8.5 Hz, 2H), 8.00 (d, *J* = 7 Hz, 1H), 7.70-7.66 (m, 3H), 7.61 (d, *J* = 9 Hz, 1H), 7.35-7.33 (m, 1H), 7.31-7.27 (m, 1H), 6.96-6.92 (m, 2H), 6.29 (d, *J* = 8.5 Hz, 1H), 4.23 (s, 2H); ^{13}C NMR (125 MHz, Acetone-d_6): δ 165.4, 144.2, 143.6, 139.7, 139.3, 134.9, 130.9, 128.2, 127.8, 127.8, 127.0, 126.4, 123.8, 120.4, 119.8, 118.2, 117.8, 114.9, 113.4, 44.3. LCMS (ESI):m/z: $[M+H]^+$:$C_{22}H_{17}ClF_3N_4O$; calculated 445.1; found 445.42.

2-chloro-*N*-(4-(3-((2-methoxyphenyl)amino)imidazo[1,2-a]pyridin-2-yl)phenyl)acetamide (16) 1124902

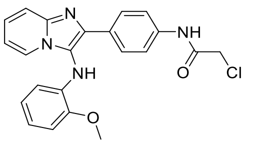

Obtained using scheme 4; 1.2 mmol scale, 51 mg, yield 13%, brown solid. ^{1}H NMR (500 MHz, $CDCl_3$): 8.49 (s, 1H), 7.98 (d, *J* = 8.5 Hz, 2H), 7.86 (d, *J* = 6.5 Hz, 1H), 7.70 (d, *J* = 9 Hz, 1H), 7.55 (d, *J* = 8.5 Hz, 2H), 7.29 (t, *J* = 7.5 Hz, 1H), 6.95 (dd, J_1 = 8.5 Hz, J_2 = 1.5 Hz, 1H), 6.85-6.81 (m, 2H), 6.70 (td, J_1 = 7.5 Hz, J_2 = 1.5 Hz, 1H), 6.16-6.15 (m, 2H), 4.17 (s, 2H), 4.39 (s, 3H); ^{13}C NMR (125 MHz, $CDCl_3$): δ 163.9, 147.1, 142.0, 137.3, 136.7, 133.6, 129.0, 127.7, 126.2, 122.9, 121.4, 120.0, 119.7, 118.0, 116.9, 112.9, 111.7, 110.5, 55.7, 42.9; HRMS (ESI) m/z: $[M+H]^+$: $C_{22}H_{20}ClN_4O_2$, calculated 407.12693; found 407.12653.

2-chloro-*N*-(4-(3-((2,6-dimethylphenyl)amino)imidazo[1,2-a]pyridin-2-yl)phenyl)acetamide (17) 1084662

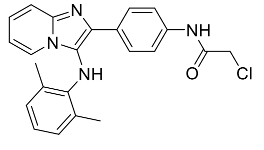

Obtained using scheme 4; 1.2 mmol scale, 260 mg, yield 54%, white solid. ^{1}H NMR (500 MHz, $CDCl_3$): 8.33 (s, 1H), 8.09 (d, *J* = 8.5 Hz, 2H), 7.66 (d, *J* = 6.5 Hz, 1H), 7.62 (d, *J* = 9 Hz, 1H), 7.55 (d, *J* = 8.5 Hz, 2H), 7.20-7.17 (m, 1H), 6.97 (d, *J* = 7.5 Hz, 2H), 6.81 (t, *J* = 7.5 Hz, 1H), 6.74 (t, *J* = 7 Hz, 1H), 5.48 (s, 1H), 4.19 (s, 2H), 2.00 (s, 6H); ^{13}C NMR (125 MHz, $CDCl_3$): δ 163.7, 141.1, 140.1, 136.1, 129.9, 127.8, 125.4, 124.9, 122.4, 121.3, 120.8, 119.9, 117.2, 112.7, 42.9, 18.5. HRMS (ESI) m/z: $[M+H]^+$: $C_{23}H_{22}ClN_4O$, calculated 405.14767; found 405.14774.

2-chloro-*N*-(4-(3-((2-ethyl-6-methylphenyl)amino)imidazo[1,2-a]pyridin-2-yl)phenyl)acetamide (18) 1124901

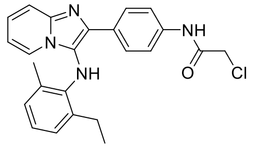

Obtained using scheme 4; 1.2 mmol scale, 140 mg, yield 28%, yellow solid. ^{1}H NMR (500 MHz, $CDCl_3$): 8.29 (s, 1H), 8.13 (d, *J* = 8.5 Hz, 2H), 7.60-7.56 (m, 4H), 7.16-7.13 (m, 1H), 7.07 (dd, J_1 = 7.5 Hz, J_2 = 2 Hz, 1H), 6.94 (dd, J_1 = 7.5 Hz, J_2 = 2 Hz, 1H), 6.87 (t, *J* = 7 Hz, 1H), 6.68 (td, J_1 = 7 Hz, J_2 = 1.5 Hz, 1H), 5.46 (s, 1H), 4.19 (s, 2H), 2.53 (q, *J* = 7.5 Hz, 2H), 1.86 (s, 3H), 1.13 (t, *J* = 7.5 Hz, 3H). ^{13}C NMR (125 MHz, $CDCl_3$): δ 163.7, 141.3, 139.5, 136.3, 135.9, 131.5, 130.6, 130.0, 127.7, 127.4, 125.8, 124.2, 122.3, 121.5, 121.1, 119.8, 117.4, 112.2, 42.9, 24.5, 18.5, 13.8. HRMS (ESI) m/z: $[M+H]^+$: $C_{24}H_{24}ClN_4O$, calculated 419.16332; found 419.16326.

2-chloro-*N*-(4-(3-((2-methoxy-6-methylphenyl)amino)imidazo[1,2-a]pyridin-2-yl)phenyl)acetamide (19) 1124904

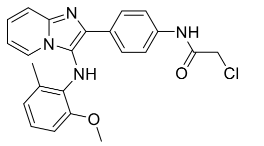

Obtained using scheme 4; 1.2 mmol scale, 135 mg, yield 27%, dark green solid. ^{1}H NMR (500 MHz, $CDCl_3$): 8.45 (s, 1H), 8.07 (d, *J* = 8.5 Hz, 2H), 7.84 (d, *J* = 7 Hz, 1H), 7.60 (d, *J* = 9 Hz, 1H), 7.53 (d, *J* = 8.5 Hz, 2H), 7.20-7.16 (m, 1H), 6.82-6.74 (m, 3H), 6.56 (d, *J* = 6.5 Hz, 1H), 6.11 (s, 1H), 4.16 (s, 2H), 3.92 (s, 3H), 1.55 (s, 3H); ^{13}C NMR (125 MHz, $CDCl_3$): δ 163.7, 148.8, 141.3, 137.0, 136.1, 131.8, 130.0, 127.9, 125.6, 124.8, 124.6, 122.7, 120.6, 120.5, 119.7, 117.0, 112.4, 108.4, 56.0, 42.9, 17.5; HRMS (ESI) m/z: $[M+H]^+$: $C_{23}H_{22}ClN_4O_2$, calculated 421.14258; found 421.14217. HRMS (ESI) m/z: $[M+H]^+$:$C_{23}H_{22}ClN_4O_2$, calculated 421.14258; found 421.14217.

2-chloro-*N*-(4-(3-((2-fluoro-6-methylphenyl)amino)imidazo[1,2-a]pyridin-2-yl)phenyl)acetamide (20) 1124906

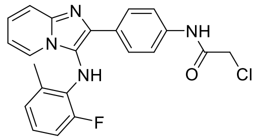

Obtained using scheme 4; 1.2 mmol scale, 52 mg, yield 11%, yellow solid. ^{1}H NMR (500 MHz, $CDCl_3$): 8.43 (s, 1H), 7.94 (d, *J* = 8.5 Hz, 2H), 7.87 (d, *J* = 7 Hz, 1H), 7.58 (d, *J* = 9 Hz, 1H), 7.50 (d, *J* = 8.5 Hz, 2H), 7.20-7.17 (m, 1H), 6.93-6.88 (m, 1H), 6.80-6.72 (m, 3H), 5.69 (d, *J* = 5 Hz, 1H), 4.14 (s, 1H), 1.82 (s, 3H); ^{13}C NMR (125 MHz, $CDCl_3$): δ 163.8, 153.0 (d, $J_{C\text{-}F}$ = 239 Hz), 141.6, 137.6, 136.2, 130.7 (d, $J_{C\text{-}F}$ = 8.5 Hz), 129.9, 127.8, 127.2 (dd, $^1J_{C\text{-}F}$ = 9.5 Hz, $^2J_{C\text{-}F}$ = 3 Hz), 125.0, 122.5, 120.6 (d, $J_{C\text{-}F}$ = 8 Hz), 119.8, 119.5, 117.2, 113.6 (d, $J_{C\text{-}F}$

= 20 Hz), 112.4, 42.9, 17.6 (d, J_{C-F} = 3 Hz); HRMS (ESI) m/z: $[M+H]^+$: $C_{22}H_{19}ClFN_4O$, calculated 409.12259; found 409.12223.

2-chloro-*N*-(4-(3-((2-chloro-6-methylphenyl)amino)imidazo[1,2-a]pyridin-2-yl)phenyl)acetamide (21) 1124907

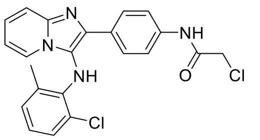

Obtained using scheme 4; 1.2 mmol scale, 294 mg, yield 58%, dark green solid. ^{1}H NMR (500 MHz, $CDCl_3$): 8.32 (s, 1H), 8.04 (d, J = 9 Hz, 2H), 7.82 (dt, J_1 = 7 Hz, J_2 = 1 Hz, 1H), 7.60 (dt, J_1 = 9 Hz, J_2 = 1 Hz, 1H), 7.55 (d, J = 9 Hz, 2H), 7.27 (dd, J_1 = 8 Hz, J_2 = 1.5 Hz, 1H), 7.22-7.18 (m, 1H), 6.84 (d, J = 7 Hz, 1H), 6.79 (td, J_1 = 7 Hz, J_2 = 1 Hz, 1H), 6.76 (t, J = 8 Hz, 1H), 6.14 (s, 1H), 4.17 (s, 2H), 1.55 (s, 3H); ^{13}C NMR (125 MHz, $CDCl_3$): δ 163.7, 141.7, 138.6, 137.8, 136.2, 131.2, 130.1, 127.9, 127.6, 127.2, 124.8, 122.8, 122.4, 121.4, 119.7, 119.3, 117.5, 112.6, 42.9, 18.2; HRMS (ESI) m/z: $[M+H]^+$: $C_{22}H_{19}Cl_2N_4O$, calculated 425.09304; found 425.09246.

2-chloro-N-(4-(3-((2,4-dichlorophenyl)amino)imidazo[1,2-a]pyridin-2-yl)phenyl)acetamide (22) 1084654

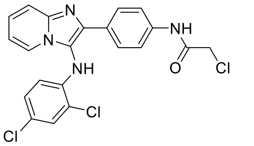

Obtained using scheme 4; 1.2 mmol scale, 117mg, yield 26%, dark green solid. ^{1}H NMR (500 MHz, $CDCl_3$): 8.37 (s, 1H), 7.94 (d, J = 9 Hz, 2H), 7.79 (d, J = 6.5 Hz, 1H), 7.66 (d, J = 9 Hz, 1H), 7.56 (d, J = 9 Hz, 2H), 7.28-7.25 (m, 1H), 6.95 (dd, J_1 = 8.5 Hz, J_2 = 2 Hz, 1H), 6.83 (t, J = 7 Hz, 1H), 6.23 (s, 1H), 6.14 (d, J = 8.5 Hz, 1H), 4.17 (s, 2H); ^{13}C NMR (125 MHz, $CDCl_3$): δ 163.8, 142.8, 139.4, 138.8, 136.7, 129.5, 129.4, 128.4, 127.6, 125.8, 124.7, 122.3, 120.1, 120.1, 117.6, 116.2, 113.9, 112.8, 42.9. HRMS (ESI) m/z: $[M+H]^+$: $C_{21}H_{16}Cl_3N_4O$, calculated 445.03842; found 445.03795.

2-chloro-*N*-(4-(3-(mesitylamino)imidazo[1,2-a]pyridin-2-yl)phenyl)acetamide (23) 1124903

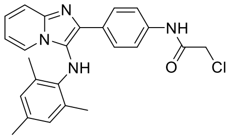

Obtained using scheme 4; 1.2 mmol scale, 184 mg, yield 37%, dark green solid. ^{1}H NMR (500 MHz, $CDCl_3$): 8.35 (s, 1H), 8.09 (d, J = 8.5 Hz, 2H), 7.62 (d, J = 7 Hz, 1H), 7.59 (d, J = 9 Hz, 1H), 7.54 (d, J = 8.5 Hz, 2H), 7.18-7.14 (m, 1H), 6.79 (s, 2H), 6.71 (td, J_1 = 6.5 Hz, J_2 = 1 Hz, 1H), 5.38 (s, 1H), 4.18 (s, 2H), 2.23 (s, 3H), 1.97 (s, 6H); ^{13}C NMR (125 MHz, $CDCl_3$): δ 163.8, 141.1, 137.5, 136.1, 135.9, 130.5, 130.4, 128.6, 127.7, 125.5, 124.4, 122.4, 121.1, 119.8, 117.2, 112.3, 42.9, 20.3, 18.4; HRMS (ESI) m/z: $[M+H]^+$: $C_{24}H_{24}ClN_4O$, calculated 419.16332; found 419.16279.

2-chloro-*N*-(4-(3-((4-chloro-2,6-dimethylphenyl)amino)imidazo[1,2-a]pyridin-2-yl)phenyl)acetamide (24) 1124908

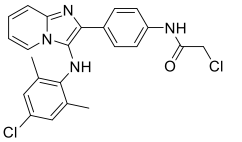

Obtained using scheme 4; 1.2 mmol scale, 73 mg, yield 17%, dark green solid. ^{1}H NMR (500 MHz, $CDCl_3$): 8.39 (s, 1H), 7.92 (d, J = 8.5 Hz, 2H), 7.71-7.68 (m, 2H), 7.51 (d, J = 8.5 Hz, 2H), 7.30-7.25 (m, 1H), 6.94 (s, 2H), 6.83 (t, J = 7 Hz), 5.69 (s, 1H), 4.18 (s, 2H), 1.95 (s, 6H); ^{13}C NMR (125 MHz, $CDCl_3$): δ 163.9, 140.5, 138.5, 136.5, 130.5, 129.4, 127.9, 127.4, 126.0, 122.5, 120.6, 119.9, 119.1, 116.6, 113.5, 42.9, 18.4; HRMS (ESI) m/z: $[M+H]^+$: $C_{23}H_{21}Cl_2N_4O$, calculated 439.10869; found 439.10839.

2-chloro-*N*-(4-(3-((4-hydroxy-2,6-dimethylphenyl)amino)imidazo[1,2-a]pyridin-2-yl)phenyl)acetamide (25) 1124909

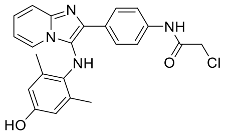

Obtained using scheme 4; 0.5 mmol scale, 113 mg, yield 55%, white solid. ^{1}H NMR (500 MHz, $CDCl_3$): δ 8.29 (s, 1H), 8.05 (d, J = 8.5 Hz, 1H), 8.01 (d, J = 9 Hz, 1H), 7.62 (t, J = 8.5 Hz, 1H), 7.58-7.52 (m, 3H), 7.18-7.13 (m, 1H), 6.75-6.71 (m, 3H), 5.36 (d, J = 18.5 Hz, 1H), 4.18 (s, 2H), 1.98 (s, 6H); ^{13}C NMR (125 MHz, $CDCl_3$): δ 170.1, 166.4, 163.9, 144.3, 143.9, 141.6, 141.5, 138.7, 138.1, 136.9, 136.2, 136.1, 130.6, 127.9, 126.8, 126.7, 124.6, 124.6, 122.6, 122.4, 122.3, 122.1, 120.6, 120.4, 120.1, 117.6, 117.6, 112.6, 112.6, 43.0, 41.0, 21.2, 18.7. LCMS (ESI):m/z: $[M+H]^+$:$C_{23}H_{22}ClN_4O_2$; calculated 421.14; found 421.46.

2-chloro-*N*-(4-(3-((2-chloro-6-methylphenyl)amino)-6-fluoroimidazo[1,2-a]pyridin-2-yl)phenyl)acetamide (26) 1132336

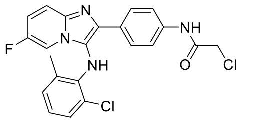

Obtained using scheme 4; 1.2 mmol scale, 77 mg, yield 15%, dark red solid. ^{1}H NMR (500 MHz, $CDCl_3$): 8.33 (s, 1H), 8.25 (s, 1H), 7.91 (d. J = 8.5 Hz, 2H), 7.76 (d, J = 9.5 Hz, 1H), 7.54 (d, J = 8.5 Hz, 2H), 7.37 (dd, J_1 = 9.5 Hz, J_2 = 2 Hz, 1H), 7.28 (dd, J_1 = 8 Hz, J_2 = 2 Hz, 1H), 6.86 (d, J = 7.5 Hz, 1H), 6.79 (t, 7.5 Hz, 1H), 6.13 (s, 1H), 4.18 (s, 2H), 1.58 (s, 3H); ^{13}C NMR (125 MHz, $CDCl_3$): δ 163.8, 141.1, 138.0, 136.8, 131.1, 129.4, 128.2, 127.8, 127.4, 123.2, 122.2, 121.6, 121.2, 120.7, 119.7, 118.7, 117.8, 117.5, 42.9, 18.3. HRMS (ESI) m/z: $[M+H]^+$: $C_{22}H_{18}Cl_2FN_4O$, calculated 443.08362; found 443.08376.

2-chloro-*N*-(4-(6-chloro-3-((2-chloro-6-methylphenyl)amino)imidazo[1,2-a]pyridin-2-yl)phenyl)acetamide (27) 1132335

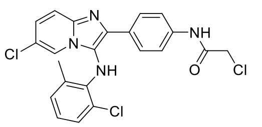

Obtained using scheme 4; 1.2 mmol scale, 252 mg, yield 46%, dark red solid. ^{1}H NMR (500 MHz, DMSO-d$_6$): 10.72 (s, 1H), 8.92 (s, 1H), 8.01-7.98 (m, 2H), 7.95-7.91 (m, 1H), 7.60 (s, 4H), 7.13 (dd, J_1 = 8 Hz, J_2 = 1.5 Hz, 1H), 7.03 (d, J = 7 Hz, 1H), 6.77 (t, J = 8 Hz, 1H), 4.28 (s, 2H), 2.00 (s, 3H); ^{13}C NMR (125 MHz, DMSO-d$_6$): δ 164.9, 155.0, 153.1, 139.4, 137.4, 134.7, 130.2, 129.5, 128.1, 127.7, 122.9, 122.9, 122.9, 122.6, 118.9, 114.1, 112.7, 112.4, 43.5, 18.4. HRMS (ESI) m/z: [M+H]$^+$: $C_{22}H_{18}Cl_3N_4O$, calculated 459.05407; found 459.05416.

2-chloro-*N*-(4-(3-((2-chloro-6-methylphenyl)amino)-6-methylimidazo[1,2-a]pyridin-2-yl)phenyl)acetamide (28) 1132334

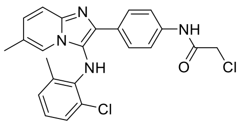

Obtained using scheme 4; 1.2 mmol scale, 164 mg, yield 31%, yellow solid. ^{1}H NMR (500 MHz, CDCl$_3$): 8.25 (s, 1H), 8.00 (d, J = 8.5 Hz, 2H), 7.66 (s, 1H), 7.55-7.51 (m, 3H), 7.28 (dd, J_1 = 7.5 Hz, J_2 = 2 Hz, 1H), 7.07 (dd, J_1 = 9.5 Hz, J_2 = 2 Hz, 1H), 6.83 (d, J = 8 Hz, 1H), 6.75 (t, J = 8 Hz, 1H), 6.09 (s, 1H), 4.18 (s, 2H), 2.30 (d, J = 1 Hz, 3H), 1.54 (s, 3H). ^{13}C NMR (125 MHz, CDCl$_3$): δ 163.6, 140.9, 138.7, 137.8, 136.0, 131.2, 130.2, 128.1, 127.8, 127.5, 126.9, 122.6, 122.4, 121.2, 120.0, 119.7, 118.9, 116.8, 42.9, 18.4, 18.2. HRMS (ESI) m/z: [M+H]$^+$: $C_{23}H_{21}Cl_2N_4O$, calculated 439.10869; found 439.10883.

2-chloro-*N*-(4-(3-((2-chloro-6-methylphenyl)amino)-6-isopropylimidazo[1,2-a]pyridin-2-yl)phenyl)acetamide (29) 1132332

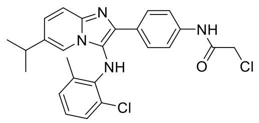

Obtained using scheme 4; 1.2 mmol scale, 213 mg, yield 38%, yellow solid. ^{1}H NMR (500 MHz, CDCl$_3$): 8.30 (s, 1H), 7.99 (d, J = 8.5 Hz, 2H), 7.68 (t, J = 1 Hz, 1H), 7.56 (d, J = 9.5 Hz), 7.53 (d, J = 8.5 Hz, 2H), 7.28 (dd, J_1 = 8 Hz, J_2 = 1.5 Hz, 1H), 7.16 (dd, J_1 = 9.5 Hz, J_2 = 2 Hz, 1H), 6.84 (d, J = 8 Hz, 1H), 6.76 (t, J = 8 Hz, 1H), 6.13 (s, 1H), 4.17 (s, 2H), 2.88 (sep, J = 7 Hz, 1H), 1.55 (s, 3H), 1.23 (d, J = 7Hz, 6H); ^{13}C NMR (125 MHz, CDCl$_3$): δ 163.6, 141.0, 138.8, 137.7, 136.1, 133.3, 131.2, 130.1, 127.8, 127.5, 127.4, 125.9, 123.0, 121.4, 119.7, 119.3, 118.4, 116.9, 42.9, 31.5, 23.3, 18.2; HRMS (ESI) m/z: [M+H]$^+$: $C_{25}H_{25}Cl_2N_4O$, calculated 467.13999; found 467.13969.

2-chloro-*N*-(4-(3-((2-chloro-6-methylphenyl)amino)-6-(trifluoromethyl)imidazo[1,2-a]pyridin-2-yl)phenyl) acetamide (30) 1132333

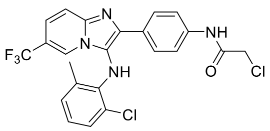

Obtained using scheme 4; 1.2 mmol scale, 21 mg, yield 4%, dark red solid. ^{1}H NMR (500 MHz, CDCl$_3$): 8.33-8.29 (m, 2H), 7.87 (d, J = 8 Hz, 2H), 7.76 (d, J = 9.5 Hz, 1H), 7.51(d, J = 8 Hz, 2H), 7.37 (dd, J_1 = 9.5 Hz, J_2 = 1.5 Hz, 1H), 7.27-7.26 (m, 1H), 6.85 (d, J = 7.5 Hz, 1H), 6.78 (t, J = 8 Hz, 1H), 6.19 (s, 1H), 4.17 (s, 2H), 1.58 (s, 3H). ^{13}C NMR (125 MHz, CDCl$_3$): δ 163.8, 141.2, 139.0, 137.9, 136.8, 131.1, 129.3, 128.5, 128.1, 127.8, 127.4, 124.5, 123.2, 122.2, 121.6 (d, $J_{C\text{-}F}$ = 5 Hz), 121.1, 120.7, 119.7, 118.7, 117.8, 42.9, 18.2. LCMS (ESI): m/z: [M+H]$^+$: $C_{23}H_{18}Cl_2F_3N_4O$; calculated 493.07; found 493.35.

2-chloro-*N*-(4-(3-((2-chloro-6-methylphenyl)amino)imidazo[1,2-a]pyridin-2-yl)-3-fluorophenyl)acetamide (31) 1132337

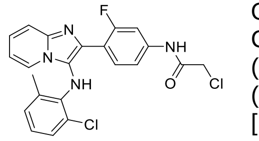

Obtained using scheme 4; 0.5 mmol scale, 55 mg, yield 25%, white solid. ^{1}H NMR (500 MHz, CDCl$_3$): δ 8.45 (s, 1H), 7.88 (d, J = 7 Hz, 1H), 7.63-7.61 (m, 2H), 7.58-7.56 (m, 1H), 7.23-7.20 (m, 1H), 7.18-7.15 (m, 3H), 6.83 (td, J_1 = 6.5 Hz, J_2 = 1 Hz, 1H), 6.78 (d, J = 7.5 Hz, 1H), 6.68 (t, J = 7.5 Hz, 1H), 6.13 (s, 1H), 4.16 (s, 2H), 1.52 (s, 3H). LCMS (ESI): m/z: [M+H]$^+$:$C_{22}H_{18}Cl_2FN_4O$; calculated 443.08; found 443.32.

2-chloro-*N*-(4-(3-((2-chloro-6-methylphenyl)amino)imidazo[1,2-a]pyridin-2-yl)benzyl)acetamide (32) 1132338

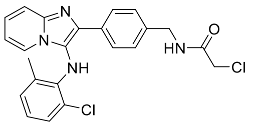

Obtained using scheme 3; 1.2 mmol scale, 185mg, yield 42%, dark green solid. ^{1}H NMR (500 MHz, DMSO-d$_6$): 8.71 (t, J = 6 Hz, 1H), 8.06 (d, J = 6.5 Hz, 1H), 7.86 (d, J = 8.5 Hz, 2H), 7.59-7.57 (m, 2H), 7.30-7.26 (m, 1H), 7.21-7.18 (m, 3H), 6.97-6.94 (m, 2H), 6.72 (t, J = 7.5 Hz, 1H), 4.28 (d, J = 6 Hz, 2H), 4.12 (s, 2H), 1.72 (s, 3H); ^{13}C NMR (125 MHz, DMSO-d$_6$): 166.0, 140.6, 139.4, 137.8, 136.6, 132.3, 130.6, 128.1, 127.3, 127.1, 126.6, 124.7, 123.2, 121.5, 120.7, 120.4, 116.6, 112.2, 42.7, 42.2, 18.2. HRMS (ESI) m/z: [M+H]$^+$: $C_{23}H_{21}Cl_2N_4O$, calculated 439.10869; found 439.10527.

2-chloro-*N*-(4-(3-((2-chloro-6-methylphenyl)amino)imidazo[1,2-a]pyridin-2-yl)cyclohexyl)acetamide (33) 1132339

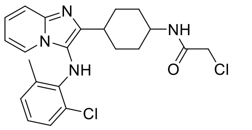

Obtained using scheme 3; 1.2 mmol scale,187 mg, yield 36%, white solid. ^{1}H NMR (500 MHz, $CDCl_3$): 8.04 (d, *J* = 7 Hz, 1H), 7.55 (d, *J* = 9 Hz, 1H), 7.28 (dd, J_1 = 8 Hz, J_2 = 1.5 Hz, 1H), 7.19-7.15 (m, 1H), 6.94 (d, *J* = 7.5 Hz, 1H), 6.85-6.81 (m, 2H), 6.33 (d, *J* = 8 Hz, 1H), 5.74 (s, 1H), 3.99 (s, 2H), 3.82-3.74 (m, 1H), 2.47-2.41 (m, 1H), 2.02 (m, 2H), 1.83-1.75 (m, 3H), 1.61 (s, 3H), 1.57 (s, 1H), 1.17-1.02 (m, 2H); ^{13}C NMR (125 MHz, $CDCl_3$): δ 164.8, 144.3, 141.4, 140.2, 130.7, 128.6, 127.4, 124.0, 123.7, 122.1, 121.9, 119.3, 117.1, 112.2, 48.5, 42.6, 35.4, 32.8, 18.1; HRMS (ESI) m/z: $[M+H]^+$: $C_{22}H_{25}Cl_2N_4O$, calculated 431.13999; found 431.13980.

4-(3-((2-chloro-6-methylphenyl)amino)imidazo[1,2-a]pyridin-2-yl)phenyl 2-chloroacetate (34) 1132341

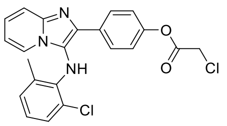

Obtained using scheme 5; 1.2 mmol scale, 316 mg, yield 62%, dark blue solid. ^{1}H NMR (500 MHz, $CDCl_3$): 8.03 (dd, J_1 = 9 Hz, J_2 = 2.5 Hz, 2H), 7.79 (d, *J* = 5 Hz), 7.62 (d, *J* = 9 Hz, 1H), 7.26-7.24 (m, 1H), 7.21-7.18 (m, 1H), 7.09 (dd, J_1 = 9 Hz, J_2 = 2.5 Hz, 2H), 6.84 (d, *J* = 7 Hz, 1H), 6.80-6.73 (m, 2H), 6.24 (s, 1H), 4.27 (s, 2H), 1.57 (s, 3H); ^{13}C NMR (125 MHz, $CDCl_3$): δ 165.6, 149.8, 141.3, 138.3, 136.7, 131.1, 130.9, 128.3, 127.6, 127.3, 125.3, 122.8, 122.5, 121.5, 120.9, 119.7, 117.2, 112.9, 40.8, 18.2; HRMS (ESI) m/z: $[M+H]^+$: $C_{22}H_{18}Cl_2N_3O_2$, calculated 426.07706; found 426.07670.

2-chloro-*N*-(4-(3-((2-chloro-6-methylphenyl)amino)imidazo[1,2-a]pyridin-2-yl)phenyl)-2,2-difluoroacetamide (35) 1132340

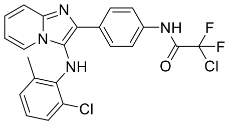

Obtained using scheme 4; 0.5 mmol scale, 46 mg, yield 20%, white solid. ^{1}H NMR (500 MHz, $CDCl_3$): δ 8.02 (d, *J* = 8 Hz, 2H), 7.75-7.73 (m, 1H), 7.60 (d, *J* = 9 Hz, 1H), 7.51 (d, *J* = 8.5 Hz, 2H), 7.18-7.14 (m, 2H), 6.82-6.71 (m, 4H), 6.17 (s, 1H), 1.54 (s, 3H).
Note: Due to the instability of the highly reactive electrophilic warhead, full spectroscopic data was not obtainable.

2,2,2-tribromo-*N*-(4-(3-((2-chloro-6-methylphenyl)amino)imidazo[1,2-a]pyridin-2-yl)phenyl)acetamide (36) 1132342

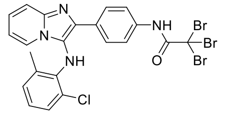

Obtained using scheme 4; 0.5 mmol scale, 69 mg, yield 22%, white solid. ^{1}H NMR (500 MHz, $CDCl_3$): δ 8.03 (d, J = 10 Hz, 1H), 7.86 (d, J = 5 Hz, 1H), 7.75-7.74 (m, 1H), 7.61-7.58 (m, 1H), 7.52-7.50 (m, 2H), 7.18-7.13 (m, 3H), 6.83-6.82 (m, 1H), 6.65-6.61 (m, 1H), 1.56 (s, 3H). *Note: Due to the instability of the highly reactive electrophilic warhead, full spectroscopic data was not obtainable.*

2-chloro-*N*-(4-(3-((2-chloro-6-methylphenyl)amino)imidazo[1,2-a]pyridin-2-yl)phenyl)-2-fluoroacetamide (37) 1132343

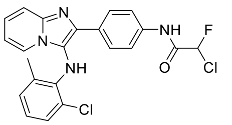

Obtained using scheme 4; 0.5 mmol scale, 55 mg, yield 25%, white solid. ^{1}H NMR (500 MHz, $CDCl_3$): δ 8.06 (d, *J* = 8.5 Hz, 2H), 7.77-7.76 (m, 1H), 7.61 (d, *J* = 9 Hz, 1H), 7.54 (d, *J* = 8 Hz, 2H), 7.20-7.17 (m, 2H), 6.83-6.71 (m, 5H), 6.16 (s, 1H), 1.55 (s, 3H). *Note: Due to the instability of the highly reactive electrophilic warhead, full spectroscopic data was not obtainable.*

2-((4-(3-((2-chloro-6-methylphenyl)amino)imidazo[1,2-a]pyridin-2-yl)phenyl)amino)-2-oxoethyl benzylsulfamate (38) 1133566

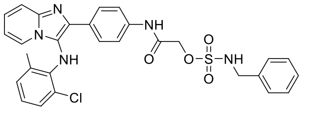

Obtained using scheme 6; 0.4 mmol scale, 23 mg, yield 10%, yellow solid. ^{1}H NMR (400 MHz, $CDCl_3$) δ 8.41 (b, 1H), 8.00 (d, *J* = 6.7 Hz, 1H), 7.76 – 7.69 (m, 3H), 7.38 – 7.33 (m, 5H), 7.30 – 7.27 (m, 2H), 7.22 – 7.17 (m, 3H), 6.97 (t, *J* = 6.7 Hz, 1H), 6.85 (d, *J* = 7.3 Hz, 1H), 6.74 (t, *J* = 7.8 Hz, 1H), 6.58 (b, 1H), 4.53 (s, 2H), 4.33 (s, 2H), 1.70 (s, 3H). LCMS (ESI): m/z calcd for $C_{29}H_{26}ClN_5O_4S$; found $[M+H]^+$ 576.07.

2-chloro-*N*-(4-(3-((2,6-dimethylphenyl)amino)-6-methylimidazo[1,2-a]pyridin-2-yl)phenyl)acetamide (39) 1132364

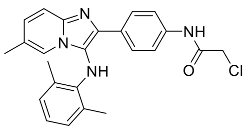

Obtained using scheme 4; 1.2 mmol scale, 243 mg, yield 49%, dark green solid. ^{1}H NMR (500 MHz, $CDCl_3$): 8.25 (s, 1H), 8.04 (d, *J* = 8.5 Hz, 2H), 7.54-7.49 (m, 4H), 7.02 (dd, J_1 = 9 Hz, J_2 = 1.5 Hz, 1H), 6.97 (d, *J* = 7.5 Hz, 2H), 6.79 (t, *J* = 7.5 Hz, 1H), 5.32 (s, 1H), 4.18 (s, 2H), 2.95 (s, 3H), 2.00 (s ,6H); ^{13}C NMR (125 MHz, $CDCl_3$): ^{13}C NMR (126 MHz, $CDCl_3$) δ 163.6, 140.6, 140.5, 135.8, 130.7, 130.0, 127.7, 124.9, 122.1, 120.9, 120.2, 120.0, 119.8, 116.9, 42.9, 18.5; HRMS (ESI) m/z: $[M+H]^+$: $C_{24}H_{24}ClN_4O$, calculated 419.16332; found 419.16289.

2-chloro-*N*-(4-(3-((2,6-dimethylphenyl)amino)imidazo[1,2-a]pyridin-2-yl)-3-fluorophenyl)acetamide (40) 1132365

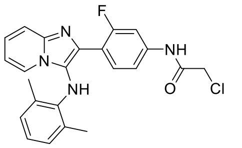

Obtained using scheme 4; 1.2 mmol scale, 316 mg, yield 63%, dark green solid. ^{1}H NMR (500 MHz, $CDCl_3$): 8.49 (s, 1H), 7.69-7.62 (m, 3H), 7.59 (dt, J_1 = 9 Hz, J_2 = 1 Hz, 1H), 7.18-7.15 (m, 2H), 6.88 (d, *J* = 7.5 Hz, 2H), 6.76-6.72 (m, 2H), 5.55 (d, *J* = 3.5 Hz, 1H), 4.18 (s, 2H), 1.90 (s, 6H); ^{13}C NMR (125 MHz, $CDCl_3$): δ 163.8, 159.8 (d, J_{C-F} = 244 Hz), 141.4, 139.7, 137.9 (d, J_{C-F} = 11 Hz), 131.8, 131.2 (d, J_{C-F} = 5 Hz), 129.4, 126.4, 124.1, 123.2, 122.4, 121.4, 117.5, 115.4 (d, J_{C-F} = 3 Hz), 112.4, 107.3 (d, J_{C-F} = 28.5 Hz), 42.8, 18.2; HRMS (ESI) m/z: [M+H]$^+$: $C_{23}H_{21}ClFN_4O$, calculated 423.13824; found 423.13791

2-chloro-*N*-(4-(3-((2,6-dimethylphenyl)amino)-6-methylimidazo[1,2-a]pyridin-2-yl)-3-fluorophenyl)acetamide (41) 1132366

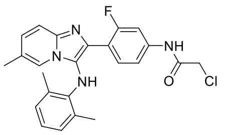

Obtained using scheme 4; 1.2 mmol scale, 382 mg, yield 73%, dark red solid. ^{1}H NMR (500 MHz, $CDCl_3$): 8.89 (s, 1H), 7.61-7.49 (m, 4H), 7.10 (t, *J* = 8 Hz, 2H), 6.84 (d, *J* = 7.5 Hz, 2H), 6.70 (t, *J* = 7 Hz, 1H), 5.68 (s, 1H), 4.17 (s, 2H), 2.27 (s, 3H), 1.89 (s, 6H); ^{13}C NMR (125 MHz, $CDCl_3$): δ 164.2, 159.7 (d, J_{C-F} = 245 Hz), 139.6, 138.5, 130.9, 129.4, 128.5, 126.3, 123.1, 121.5, 120.3, 119.4, 116.1, 115.2, 107.2, 107.0, 53.7, 43.0, 18.4, 18.2; HRMS (ESI) m/z: [M+H]$^+$: $C_{24}H_{23}ClFN_4O$, calculated 437.15389; found 437.15367.

2-chloro-*N*-(4-(6-chloro-3-((2,6-dimethylphenyl)amino)imidazo[1,2-a]pyridin-2-yl)-3-fluorophenyl)acetamide (42) 1132367

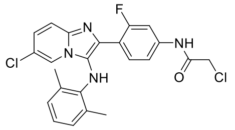

Obtained using scheme 4; 1.2 mmol scale 511 mg, yield 93%, dark red solid. ^{1}H NMR (500 MHz, $CDCl_3$): 8.49 (s, 1H), 7.74 (d, *J* = 2Hz, 1H), 7.60-7.57 (m, 2H), 7.51 (d, *J* = 9.5 Hz, 1H), 7.14-7.10 (m, 2H), 6.87 (d, *J* = 7.5 Hz, 2H), 6.73 (t, *J* = 7.5 Hz, 1H), 5.56 (d, *J* = 3 Hz, 1H), 4.15 (s, 2H), 1.90 (s, 6H); ^{13}C NMR (125 MHz, $CDCl_3$): δ 163.9, 159.7 (d, J_{C-F} = 245 Hz), 139.7, 139.2, 138.1 (d, J_{C-F} = 11.5 Hz), 132.8, 131.0 (d, J_{C-F} = 5 Hz), 129.4, 126.5, 125.4, 123.6, 121.8, 120.7, 120.2, 117.9, 115.3 (d, J_{C-F} = 3 Hz), 107.2 (d, J_{C-F} = 28 Hz), 42.8, 18.1; HRMS (ESI) m/z: [M+H]$^+$: $C_{23}H_{20}Cl_2FN_4O$, calculated 457.09927; found 457.09914.

REPRESENTATIVE NMR SPECTRA

***N*-(4-(3-(benzylamino)imidazo[1,2-a]pyridin-2-yl)phenyl)-2-chloroacetamide (1) 1076388**

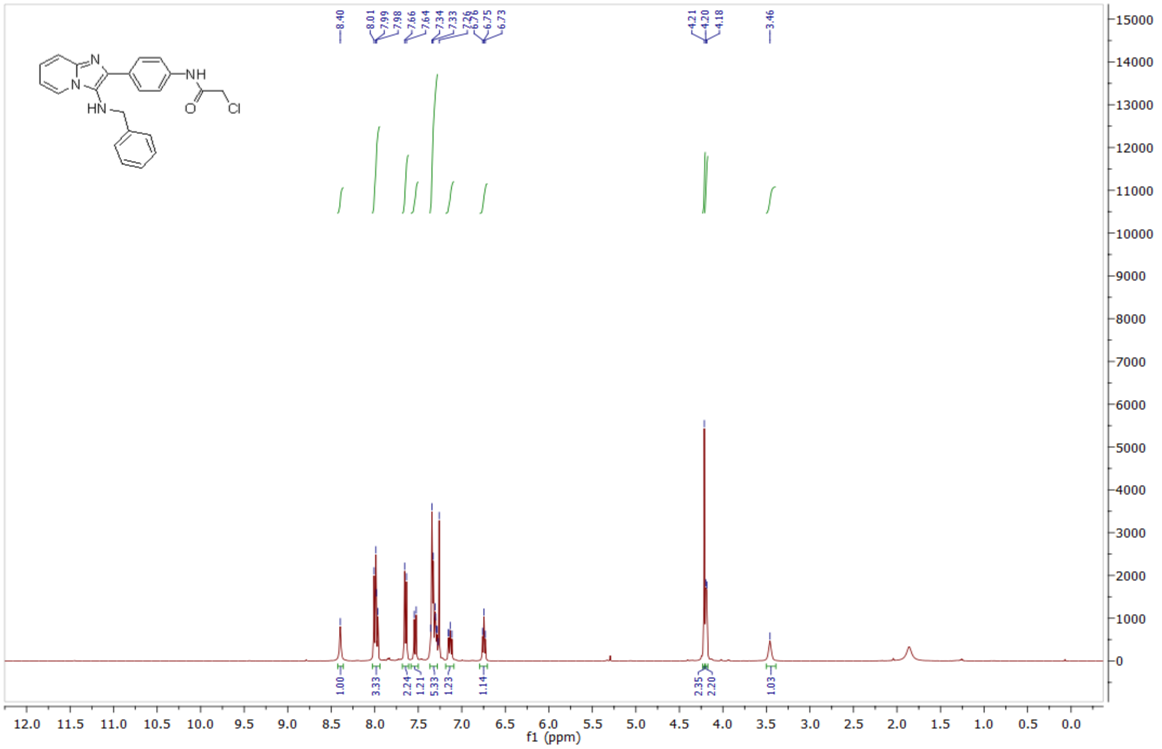

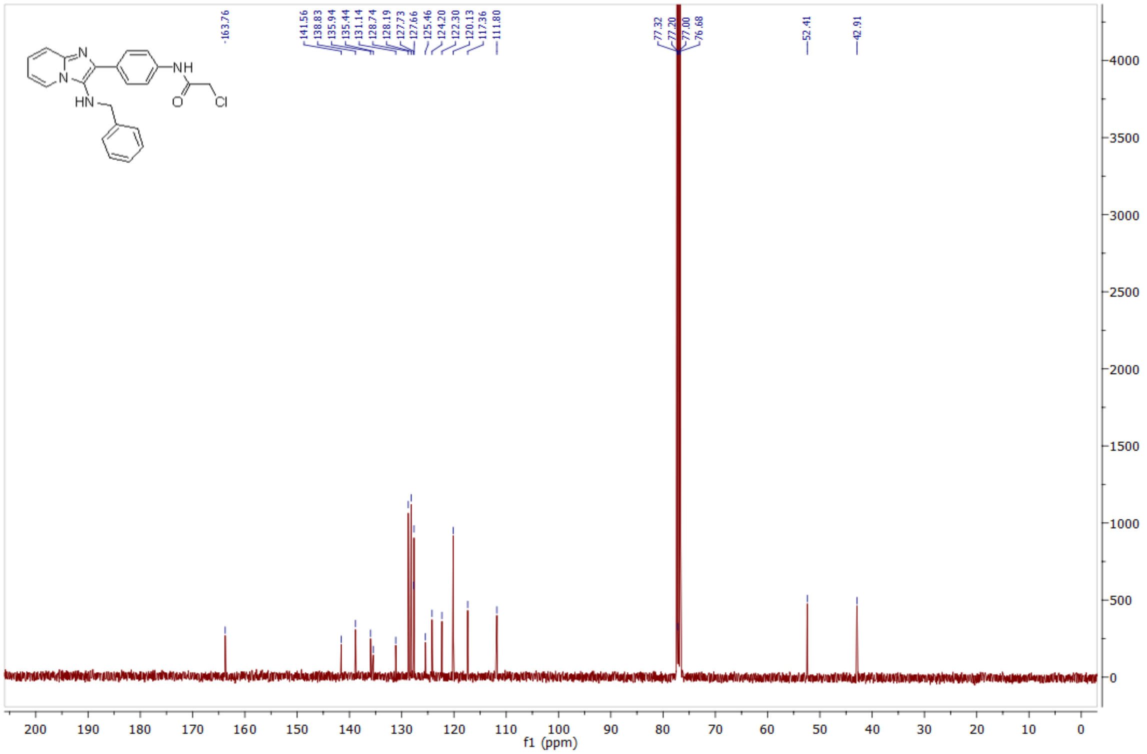

2-chloro-*N*-(4-(3-((2-fluorobenzyl)amino)imidazo[1,2-a]pyridin-2-yl)phenyl)acetamide (2) 1083860

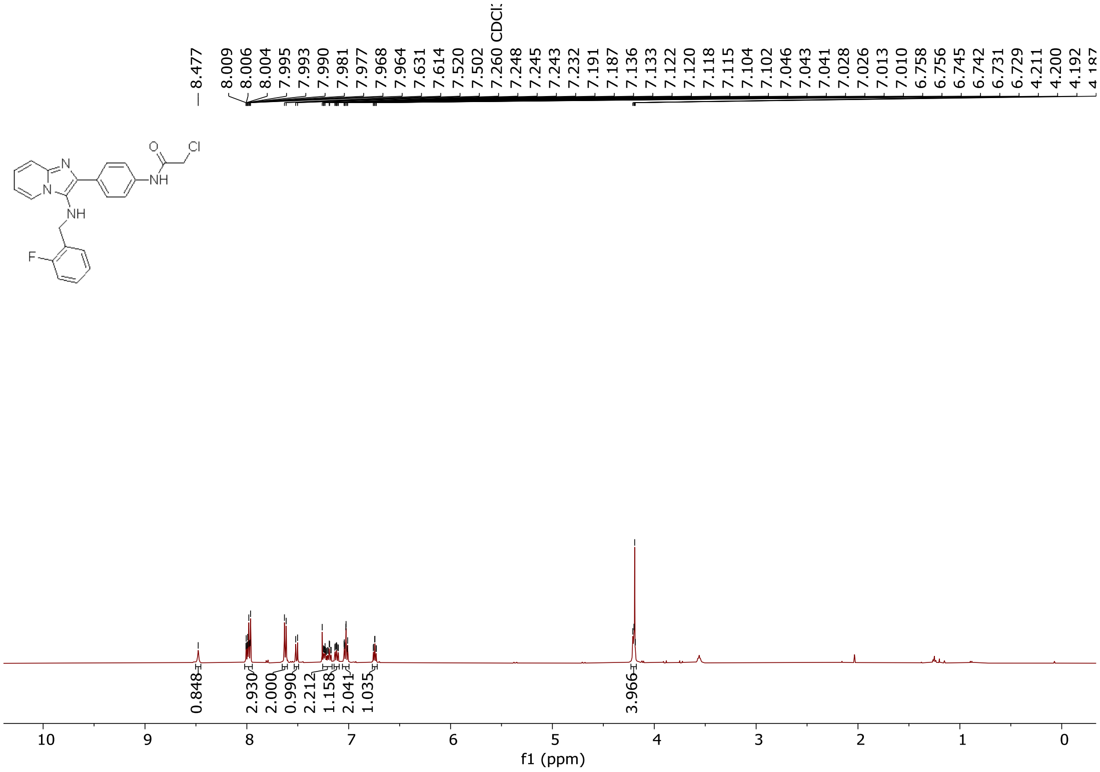

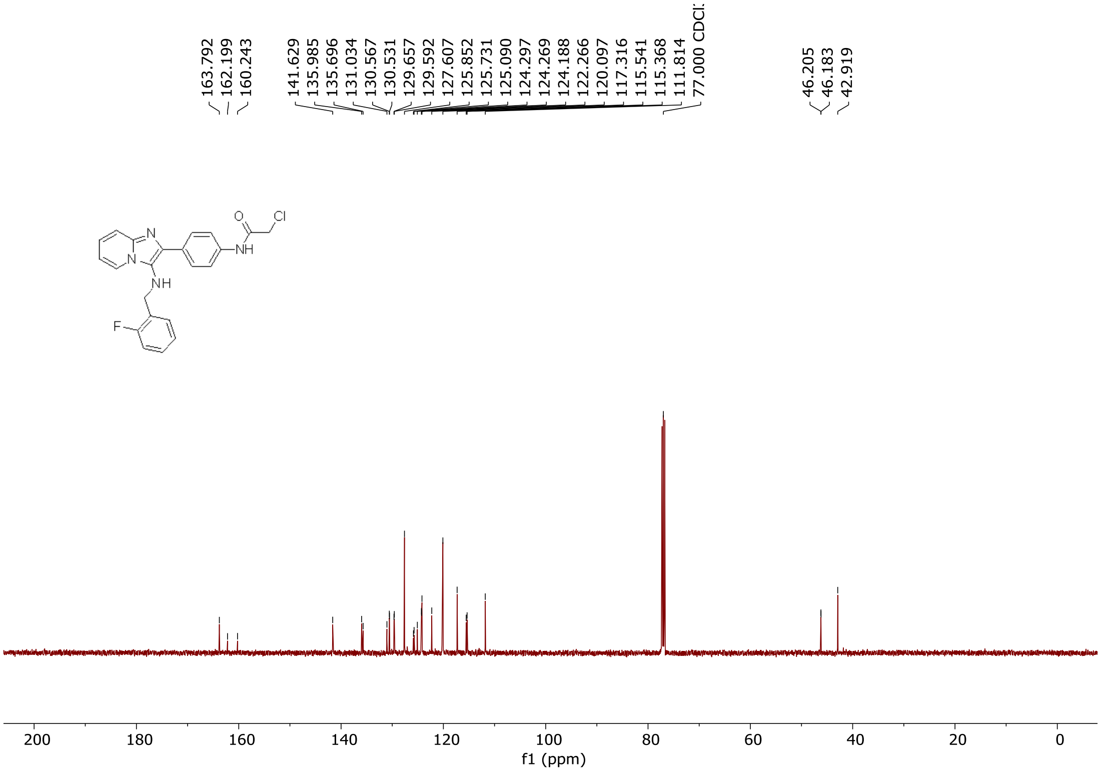

2-chloro-*N*-(4-(3-((3-fluorobenzyl)amino)imidazo[1,2-a]pyridin-2-yl)phenyl)acetamide (3) 1083861

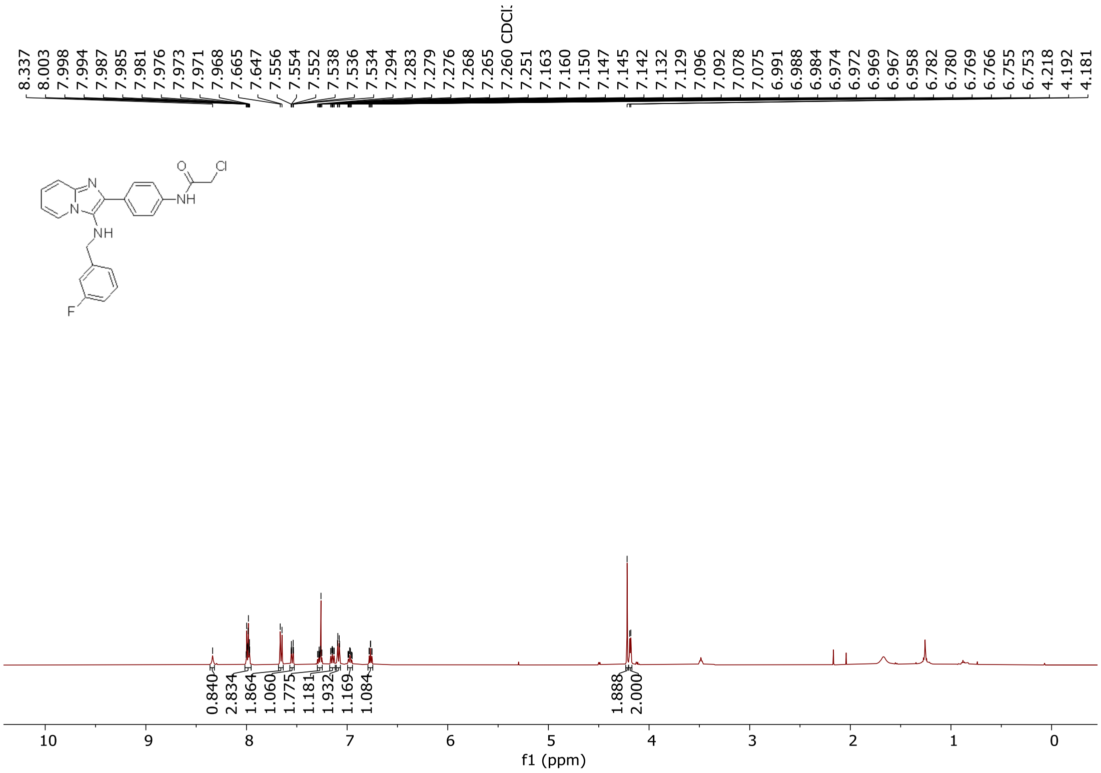

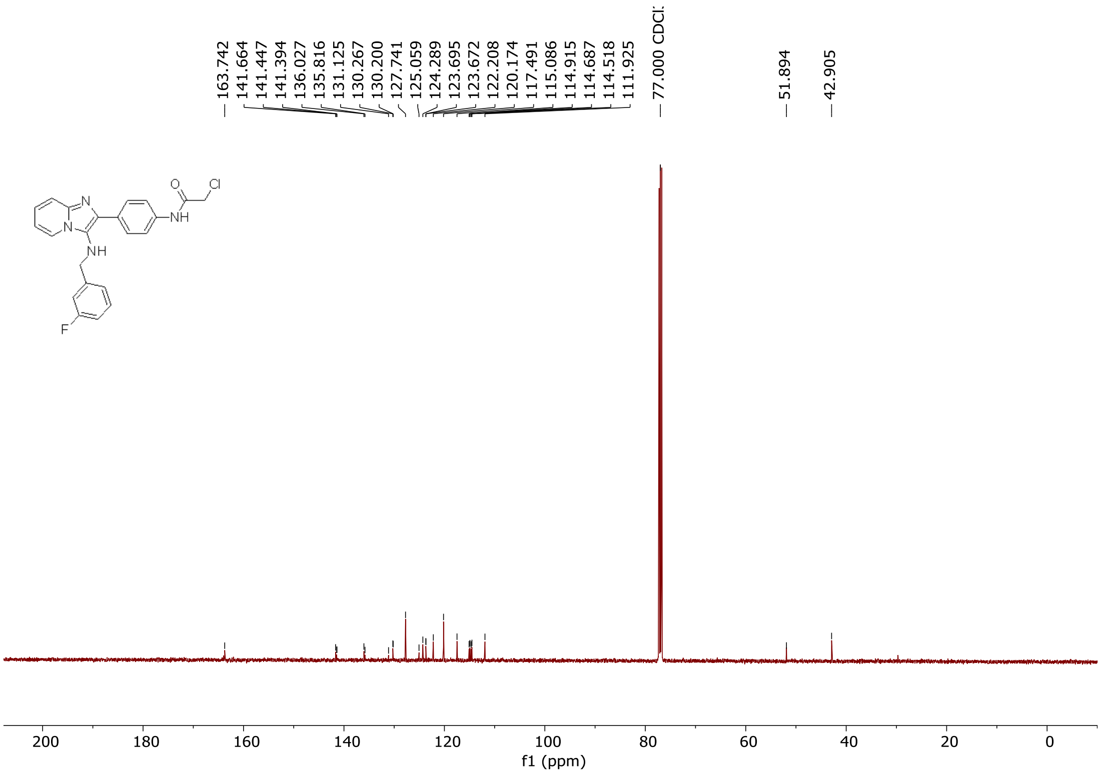

2-chloro-*N*-(4-(3-((4-fluorobenzyl)amino)imidazo[1,2-a]pyridin-2-yl)phenyl)acetamide (4) 1083857

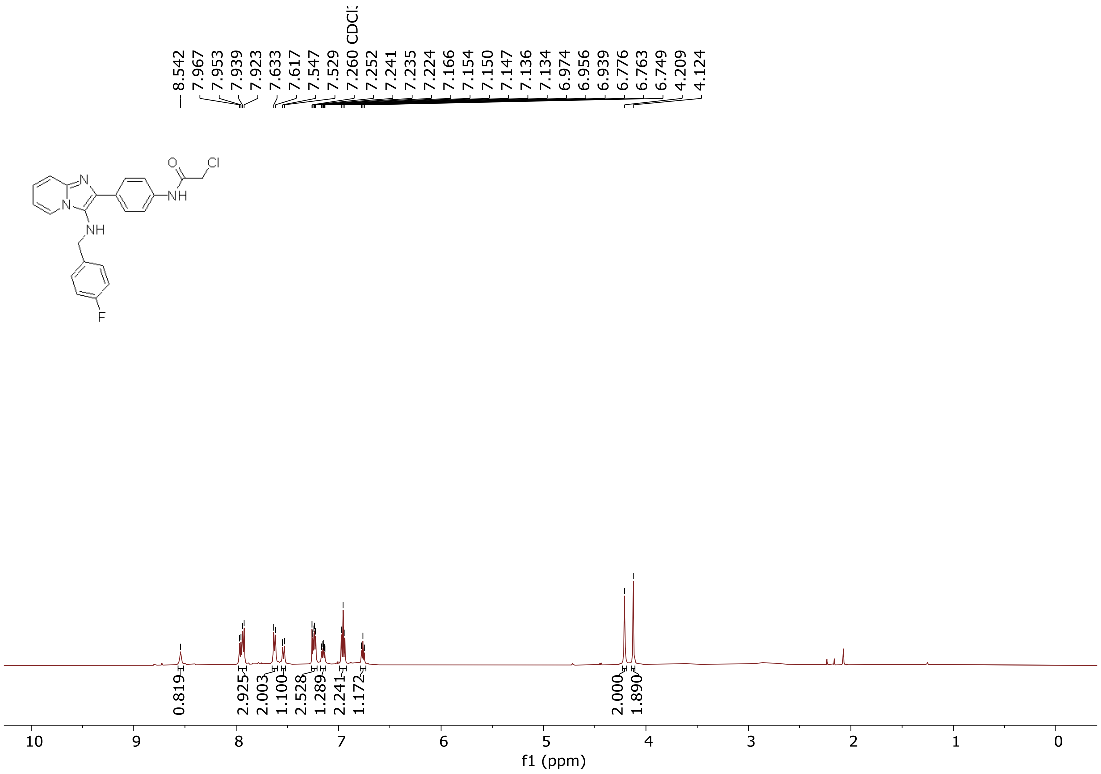

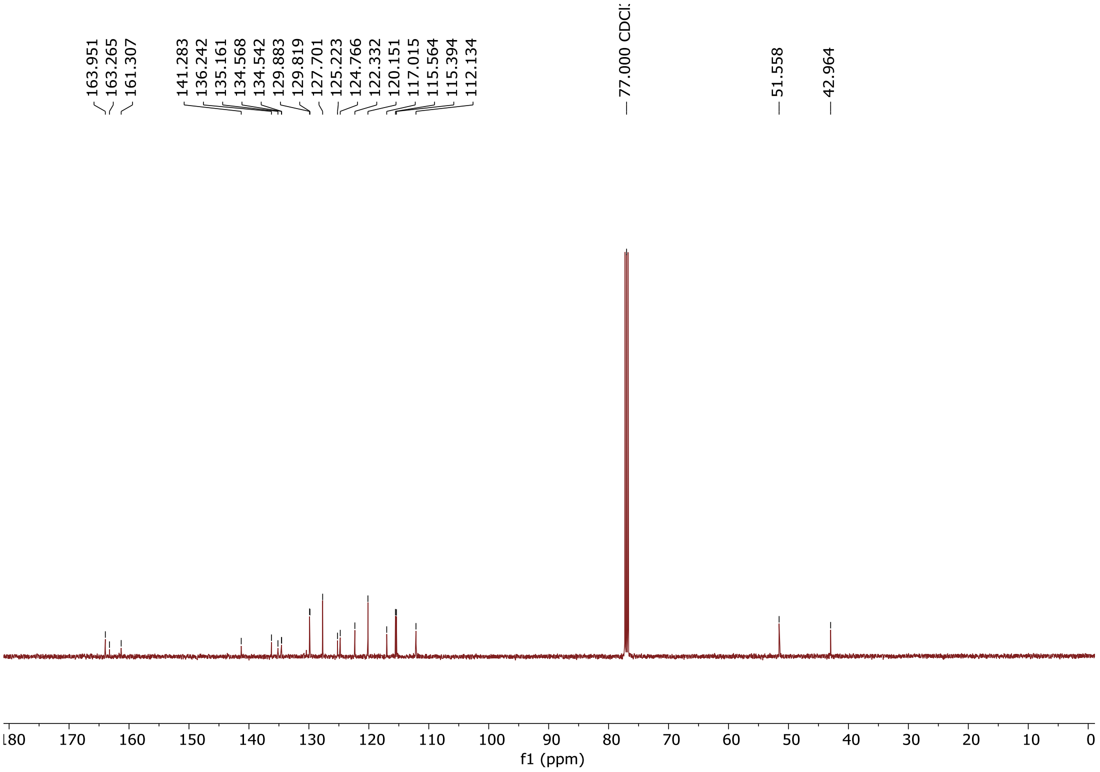

2-chloro-*N*-(4-(3-((2,6-difluorobenzyl)amino)imidazo[1,2-a]pyridin-2-yl)phenyl)acetamide (5) 1084657

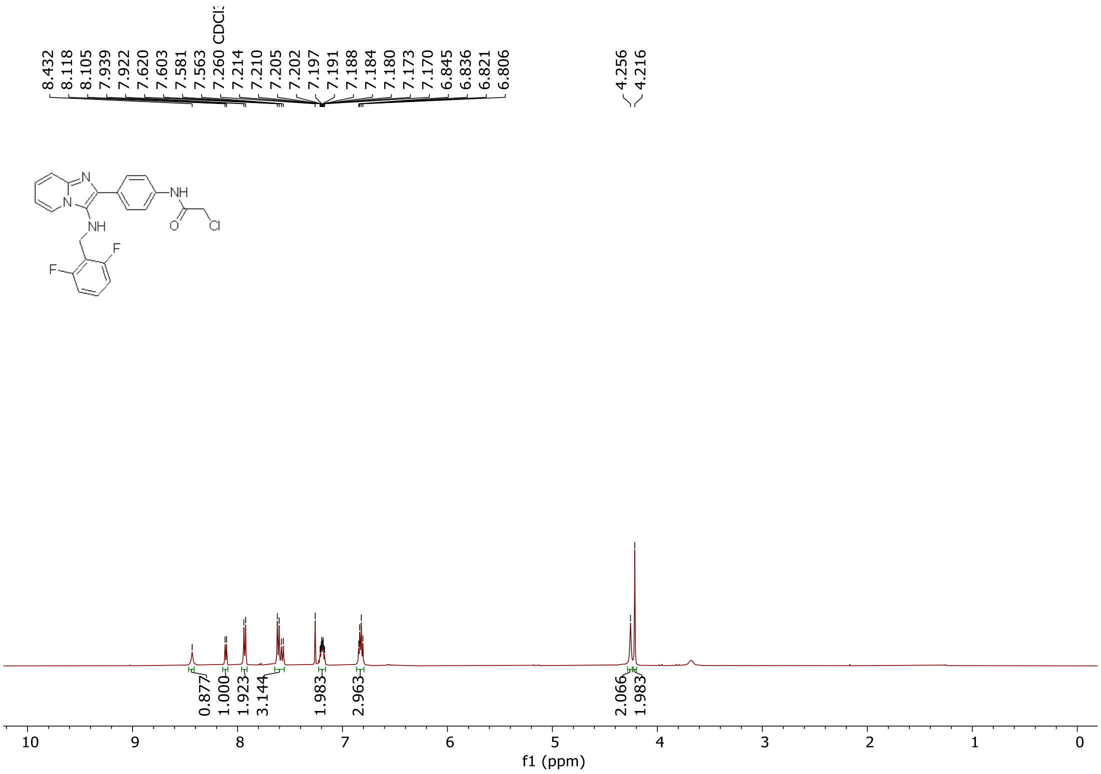

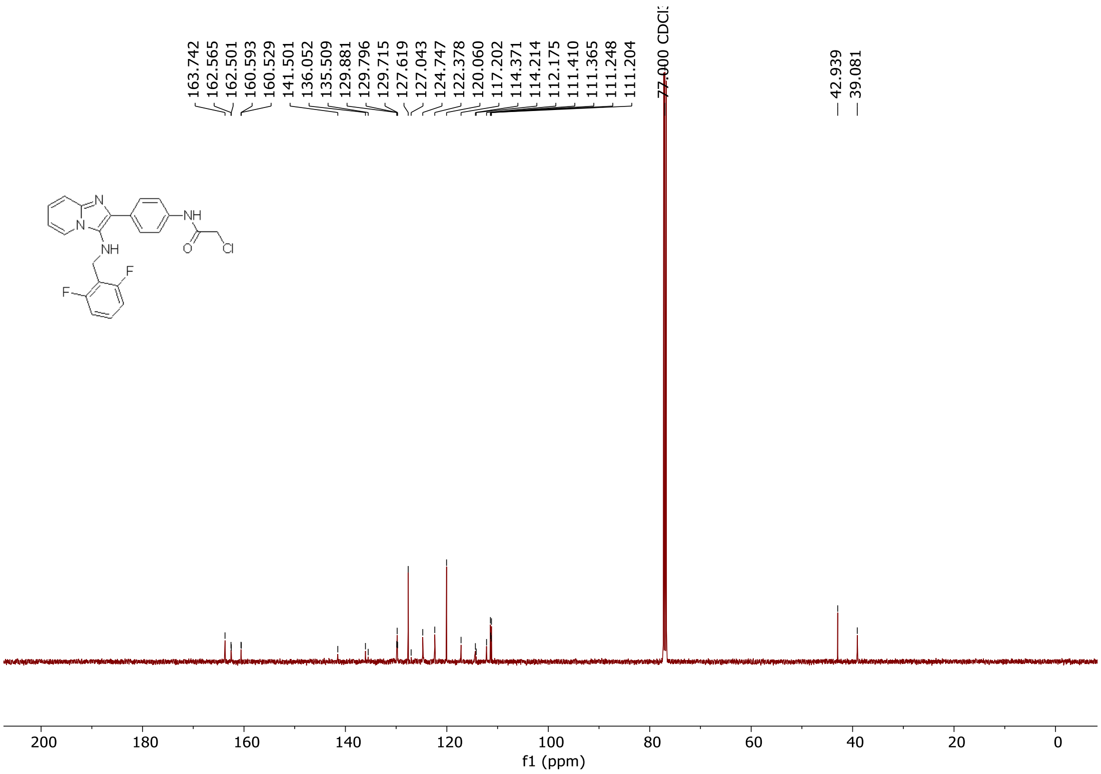

2-chloro-N-(4-(3-((3-chlorobenzyl)amino)imidazo[1,2-a]pyridin-2-yl)phenyl)acetamide (7) 1083859

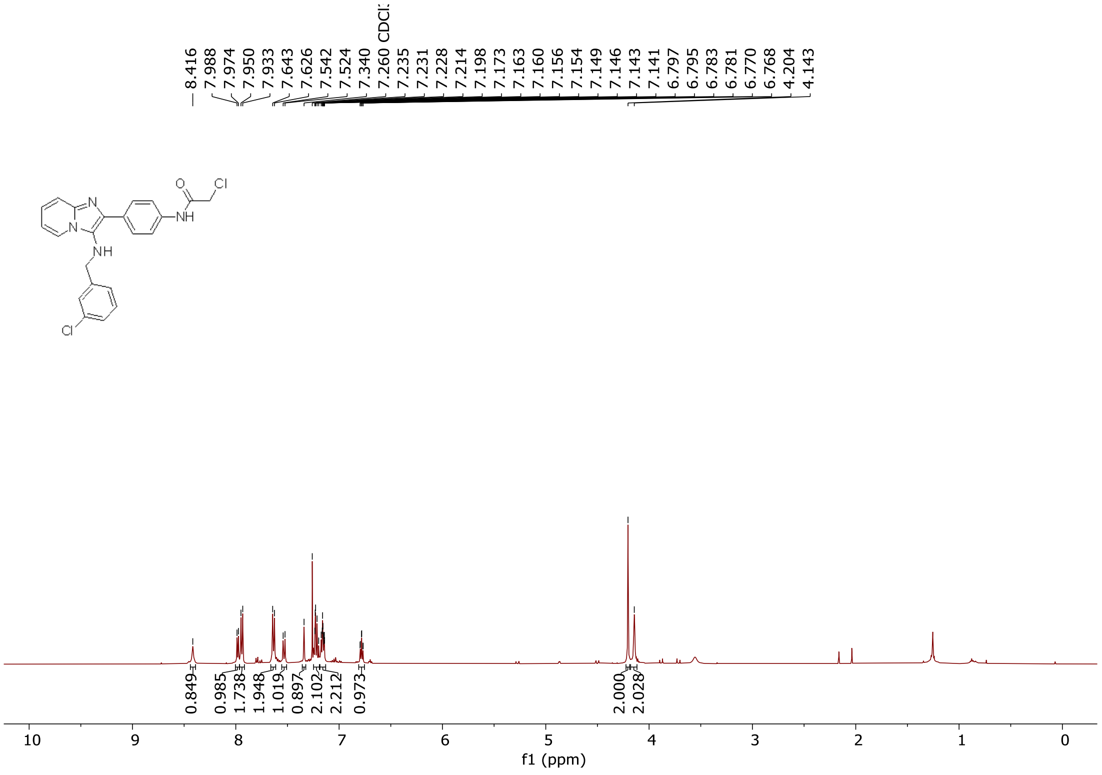

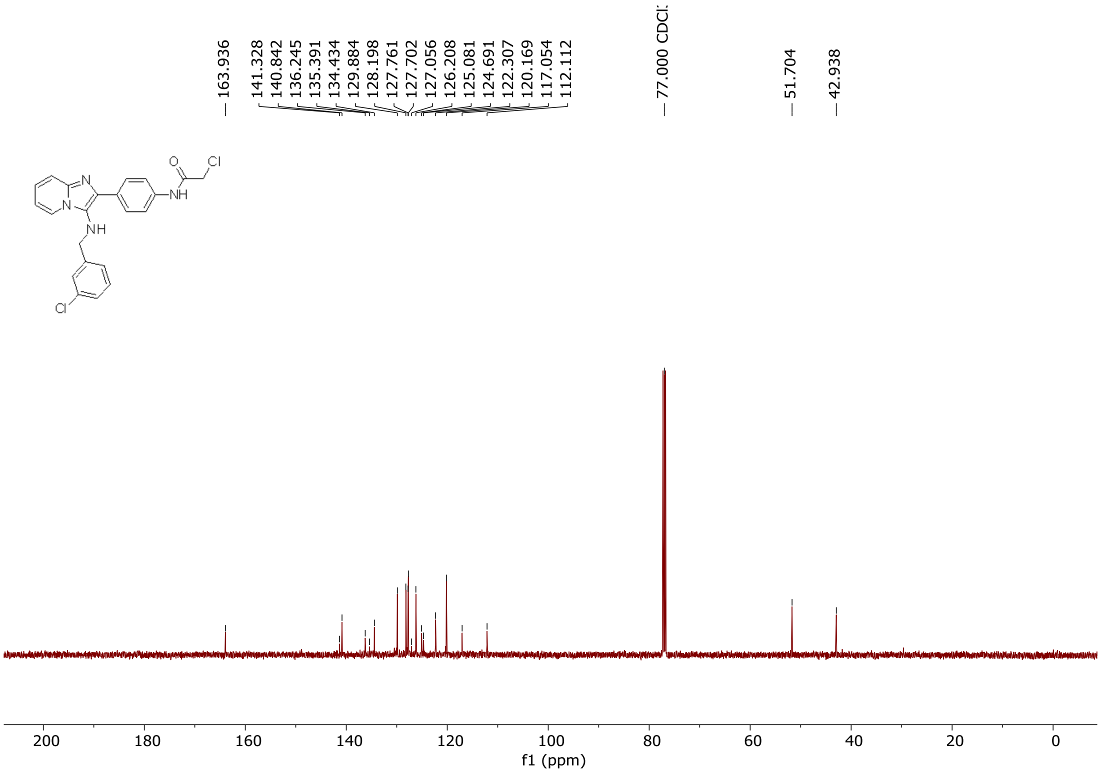

2-chloro-*N*-(4-(3-((2-nitrobenzyl)amino)imidazo[1,2-a]pyridin-2-yl)phenyl)acetamide (9) 1083866

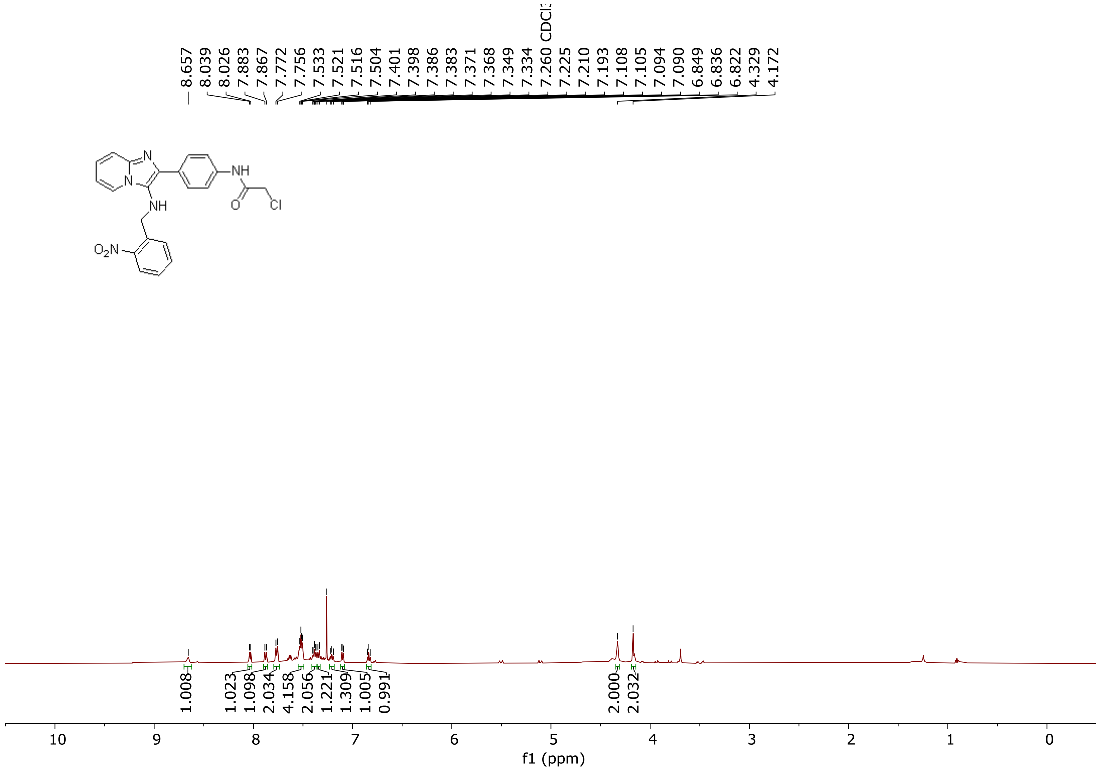

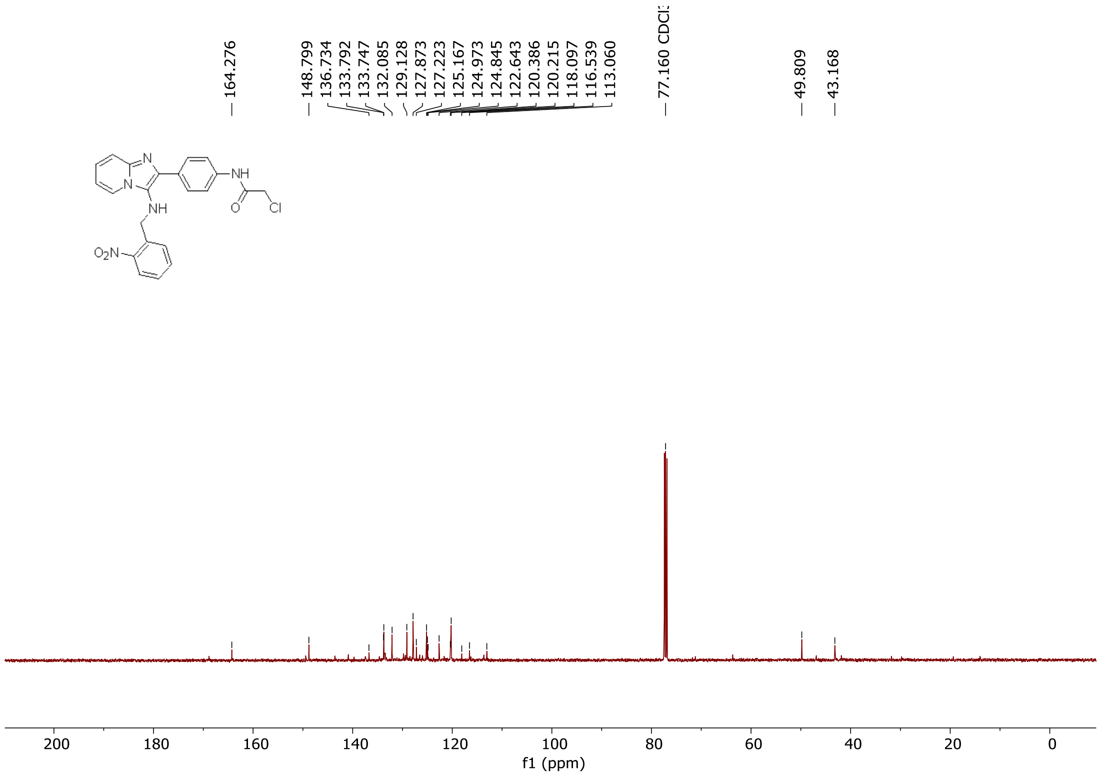

2-chloro-*N*-(4-(3-(phenylamino)imidazo[1,2-a]pyridin-2-yl)phenyl)acetamide (10) 1124905

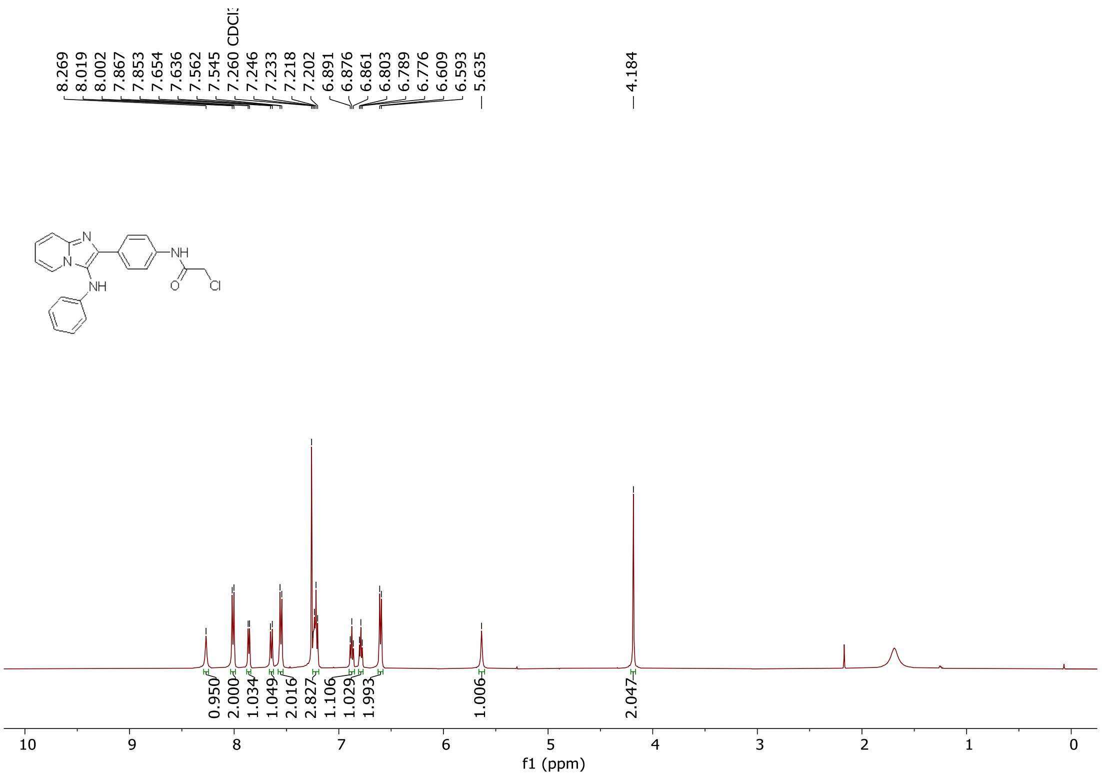

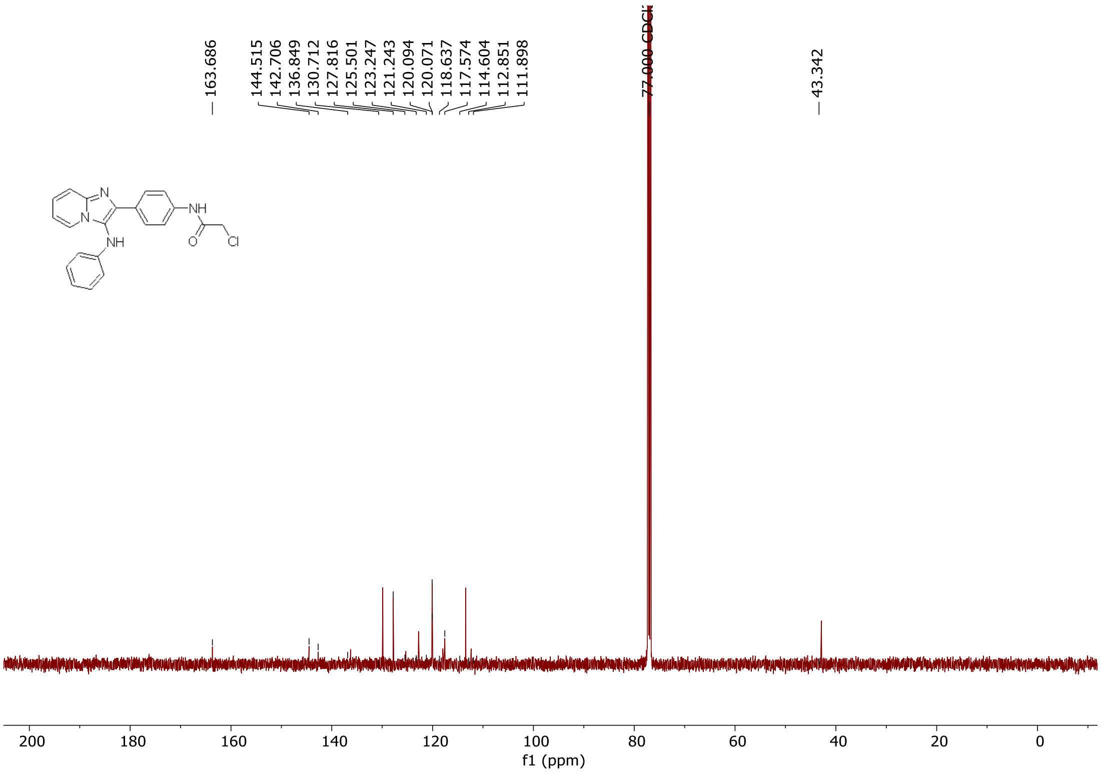

2-chloro-N-(4-(3-((4-chlorophenyl)amino)imidazo[1,2-a]pyridin-2-yl)phenyl)acetamide (11) 1083865

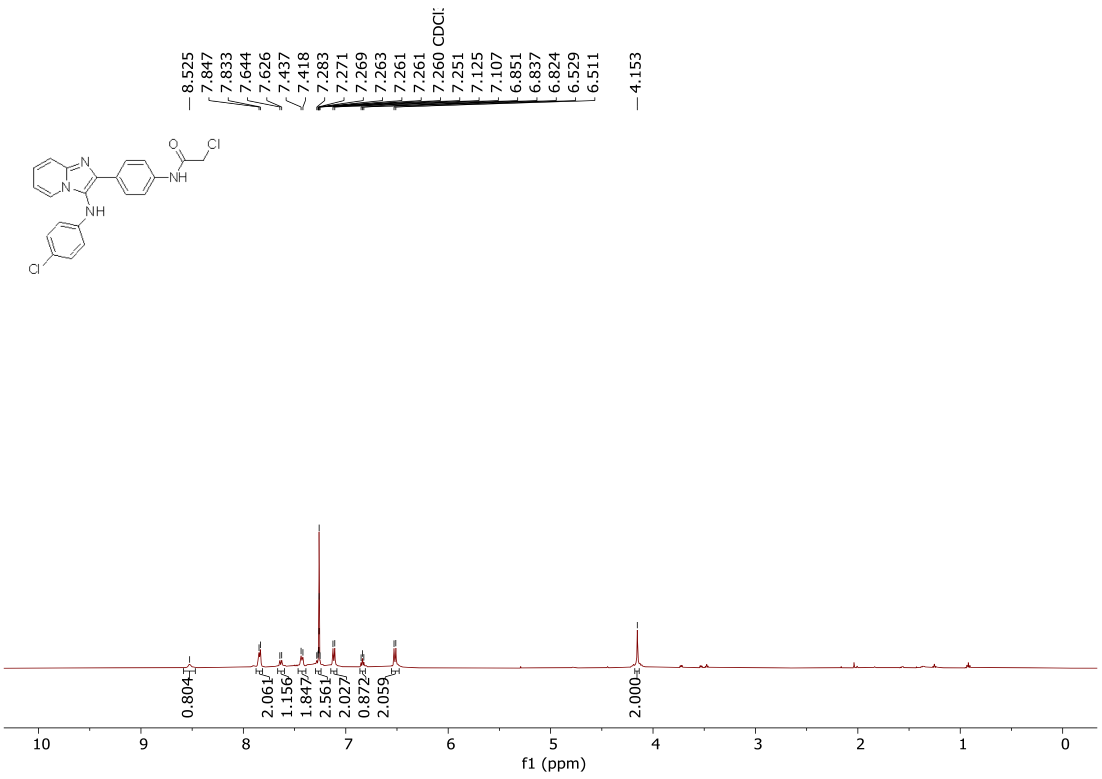

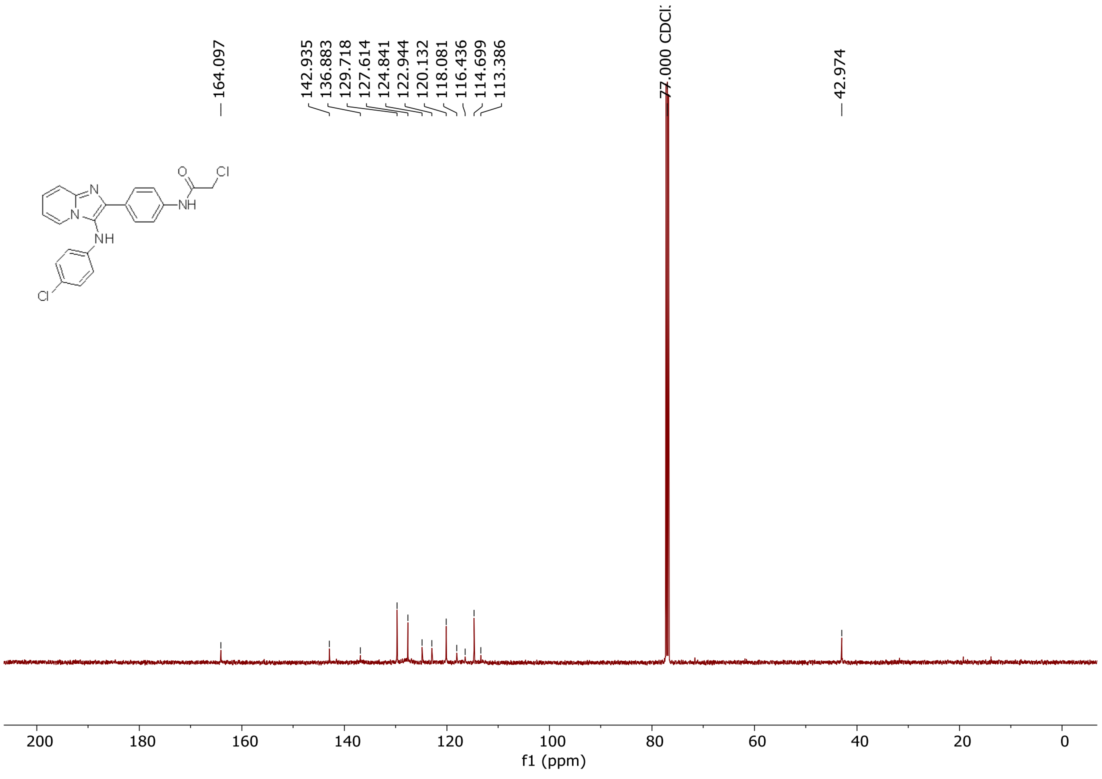

2-chloro-*N*-(4-(3-((2-(trifluoromethyl)phenyl)amino)imidazo[1,2-a]pyridin-2-yl)phenyl)acetamide (15) 1124900

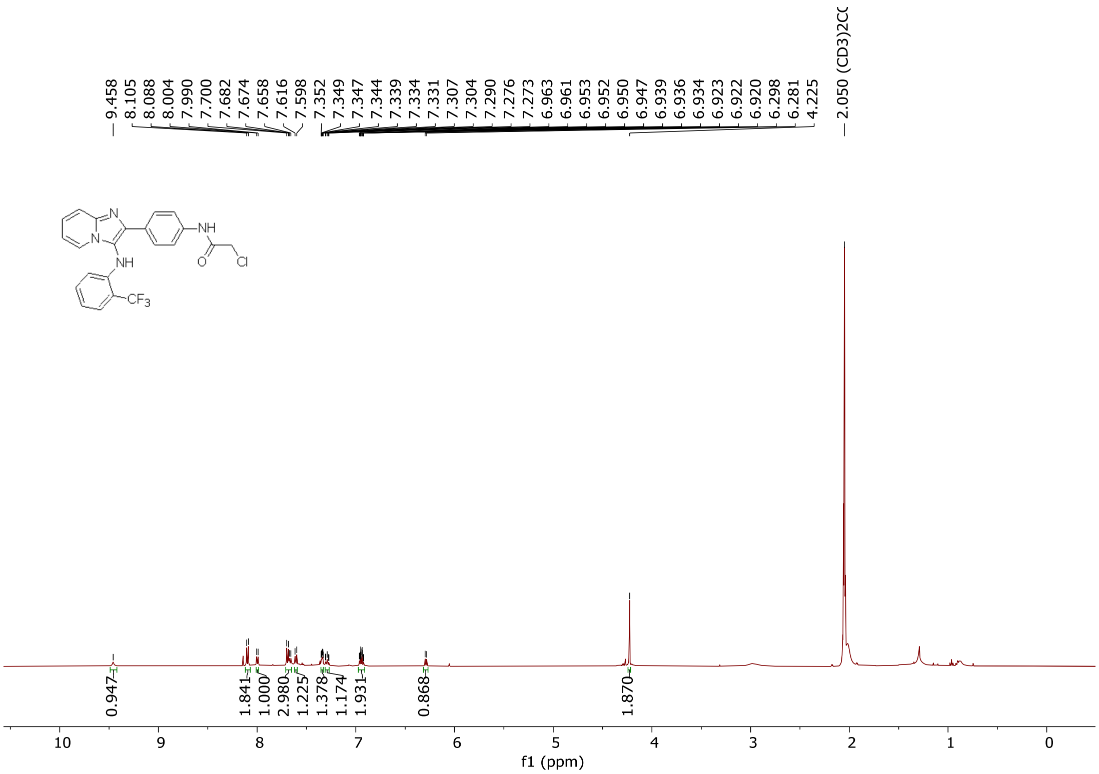

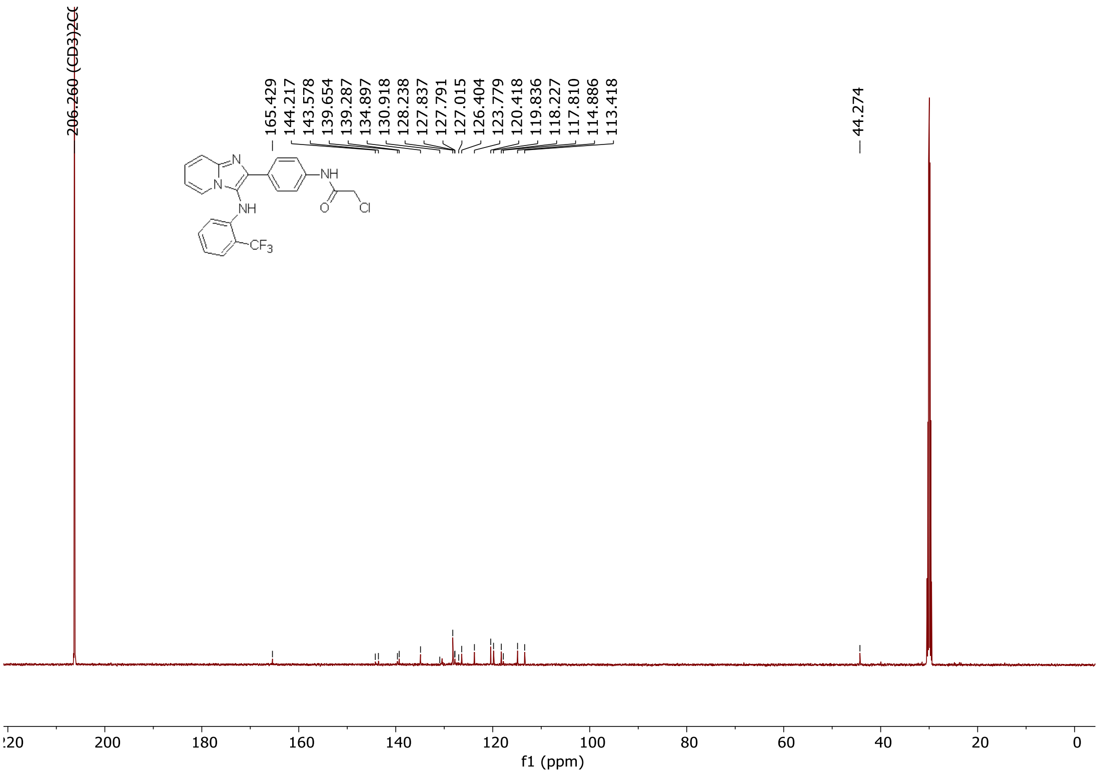

2-chloro-*N*-(4-(3-((2-methoxyphenyl)amino)imidazo[1,2-a]pyridin-2-yl)phenyl)acetamide (16) 1124902

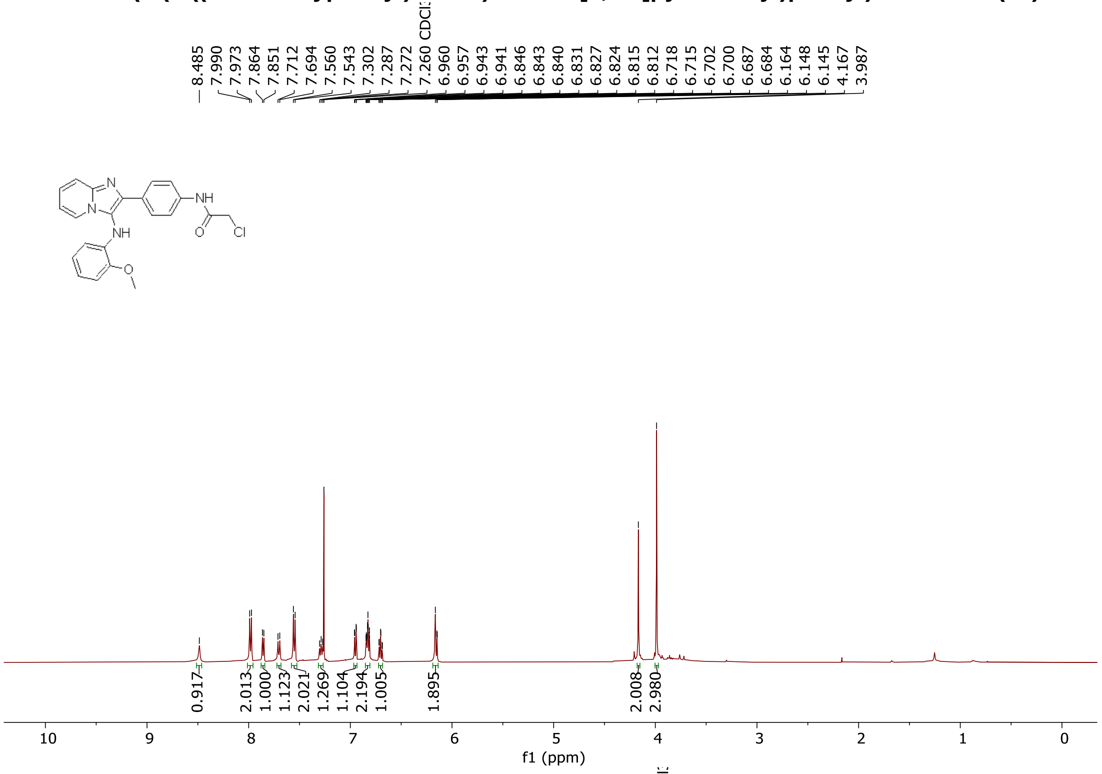

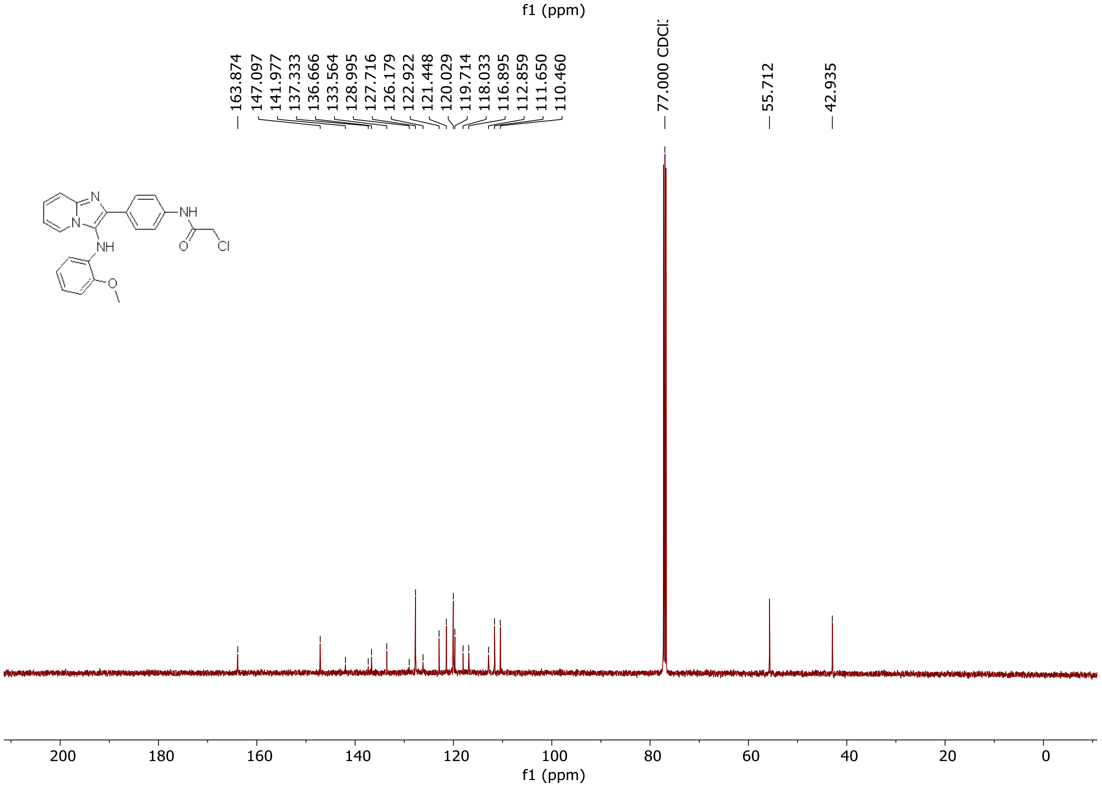

2-chloro-*N*-(4-(3-((2,6-dimethylphenyl)amino)imidazo[1,2-a]pyridin-2-yl)phenyl)acetamide (17) 1084662

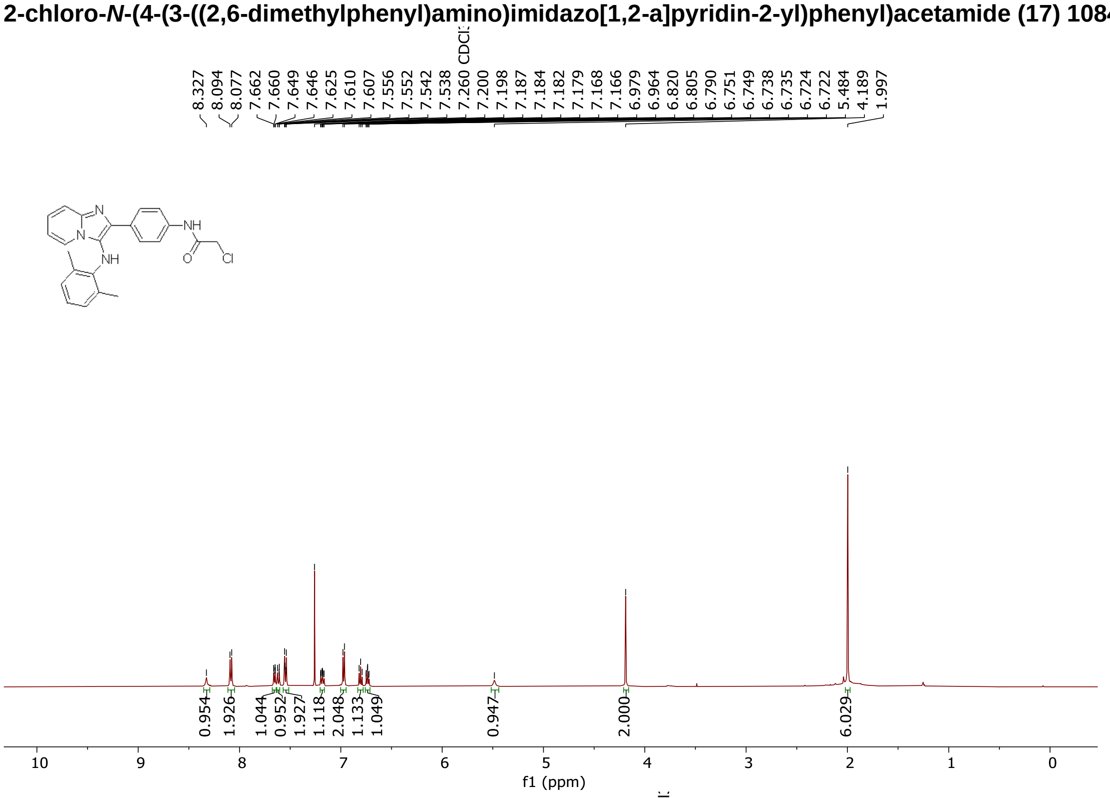

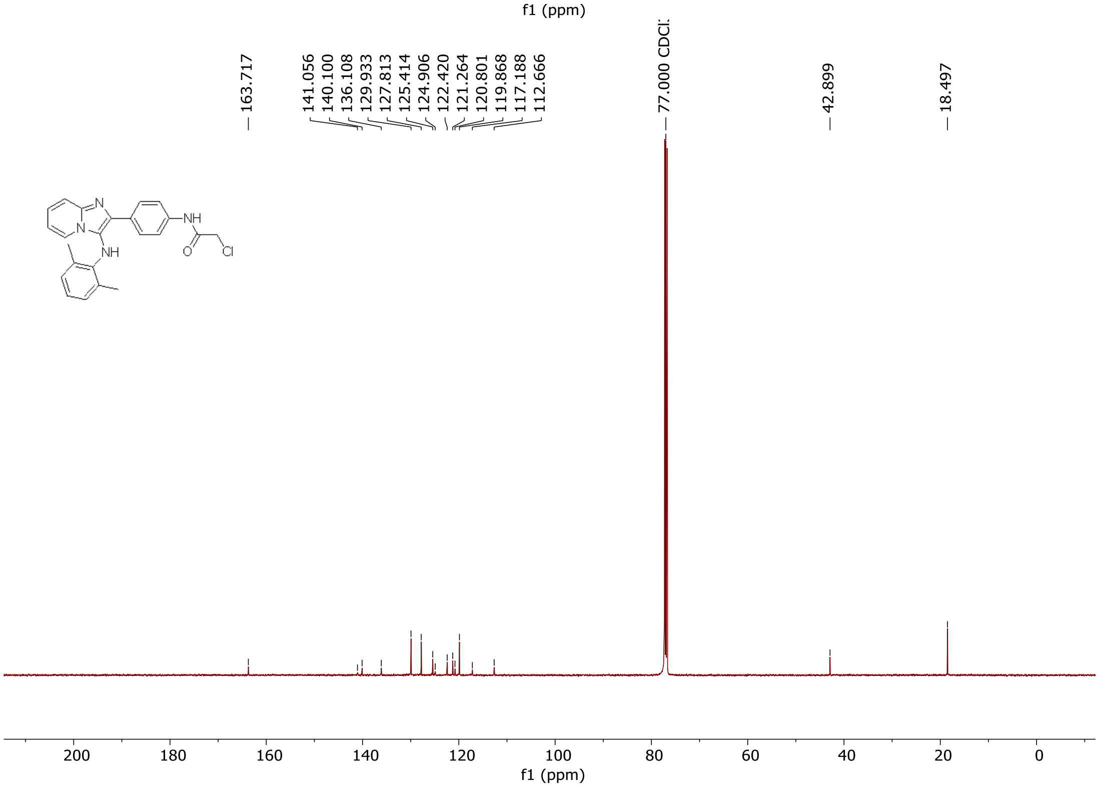

2-chloro-*N*-(4-(3-((2-ethyl-6-methylphenyl)amino)imidazo[1,2-a]pyridin-2-yl)phenyl)acetamide (18) 1124901

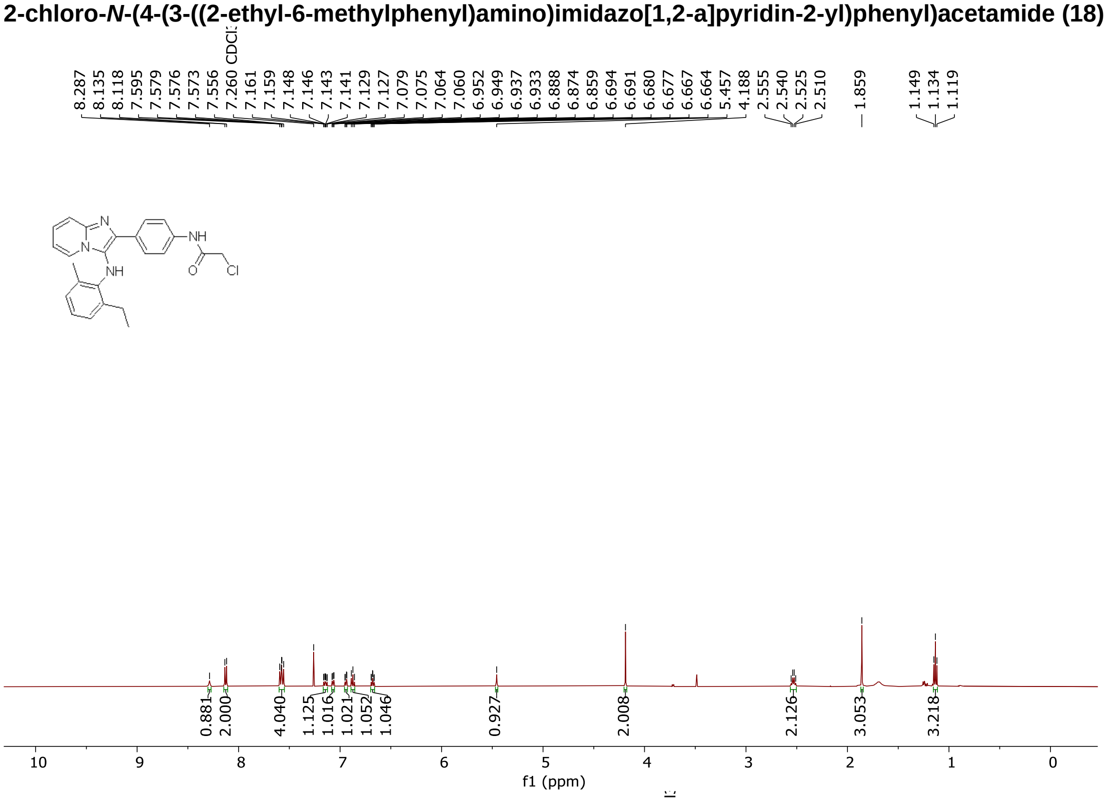

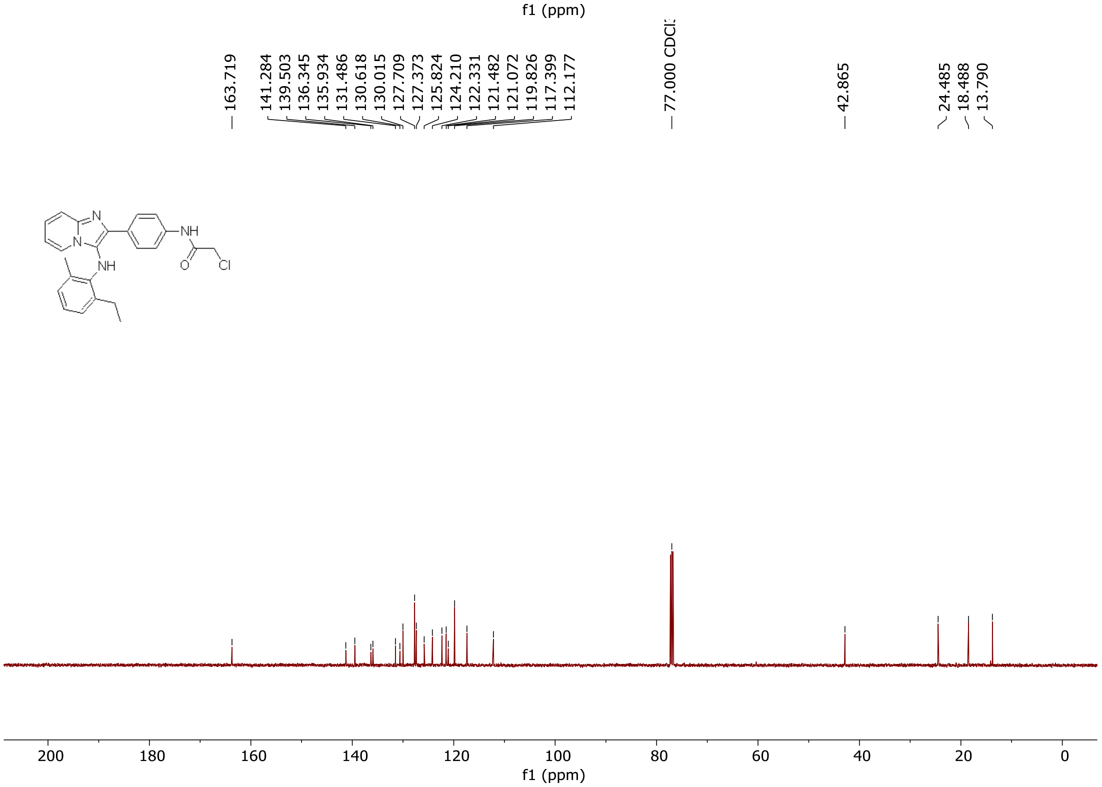

2-chloro-N-(4-(3-((2-methoxy-6-methylphenyl)amino)imidazo[1,2-a]pyridin-2-yl)phenyl)acetamide (19) 1124904

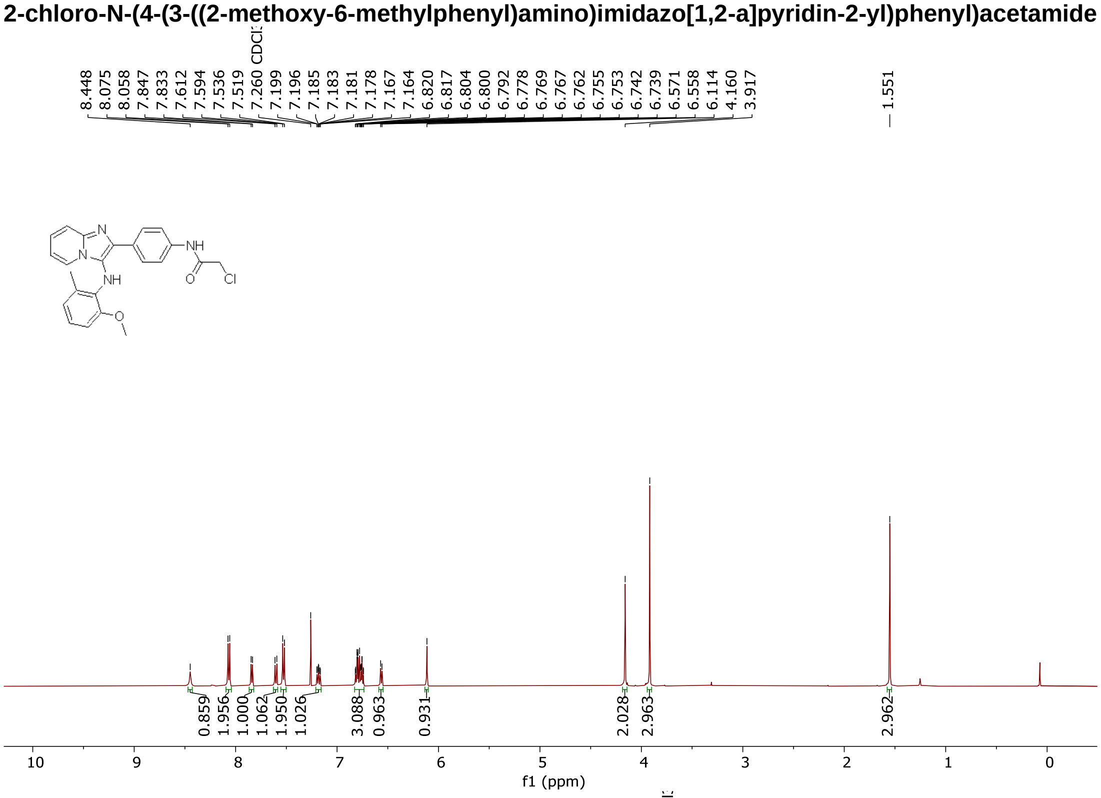

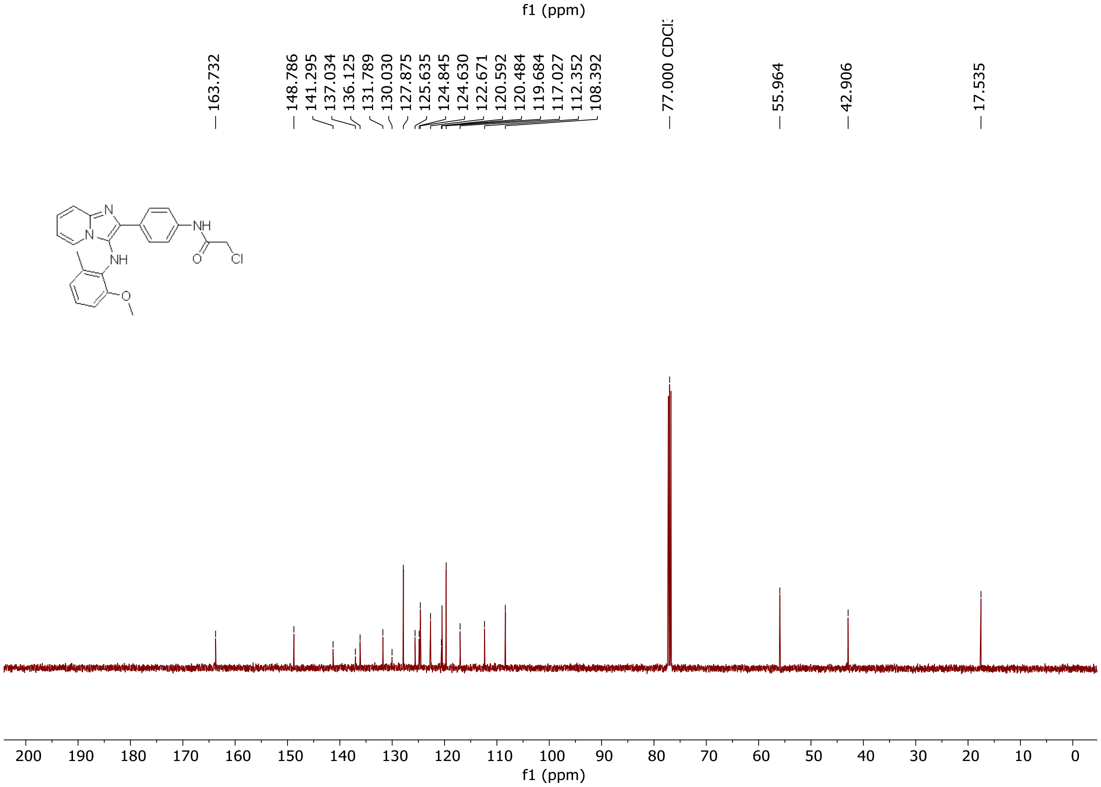

2-chloro-*N*-(4-(3-((2-fluoro-6-methylphenyl)amino)imidazo[1,2-a]pyridin-2-yl)phenyl)acetamide (20) 1124906

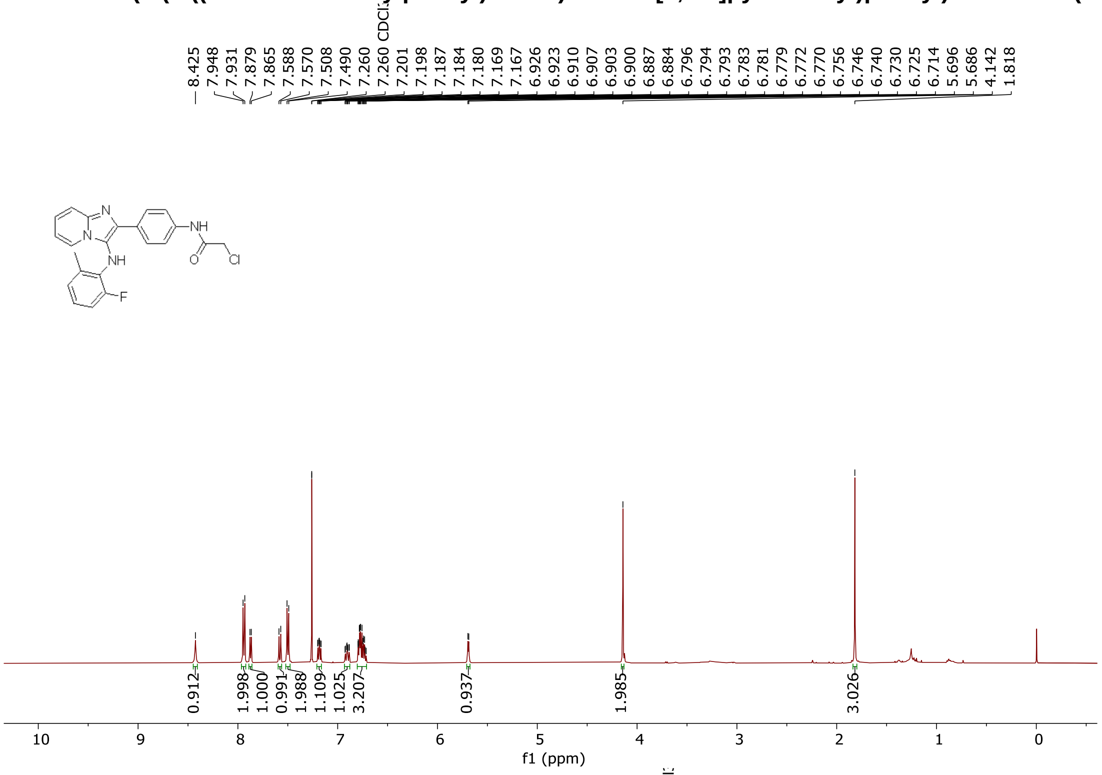

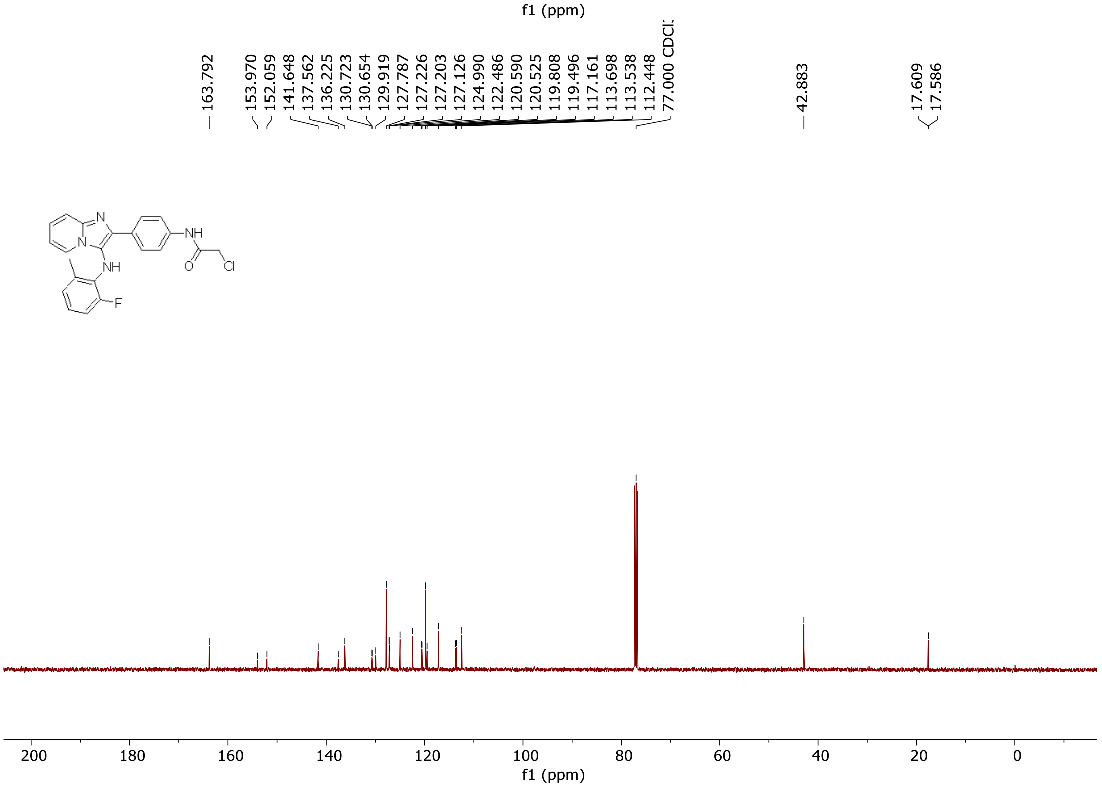

2-chloro-*N*-(4-(3-((2-chloro-6-methylphenyl)amino)imidazo[1,2-a]pyridin-2-yl)phenyl)acetamide (21) 1124907

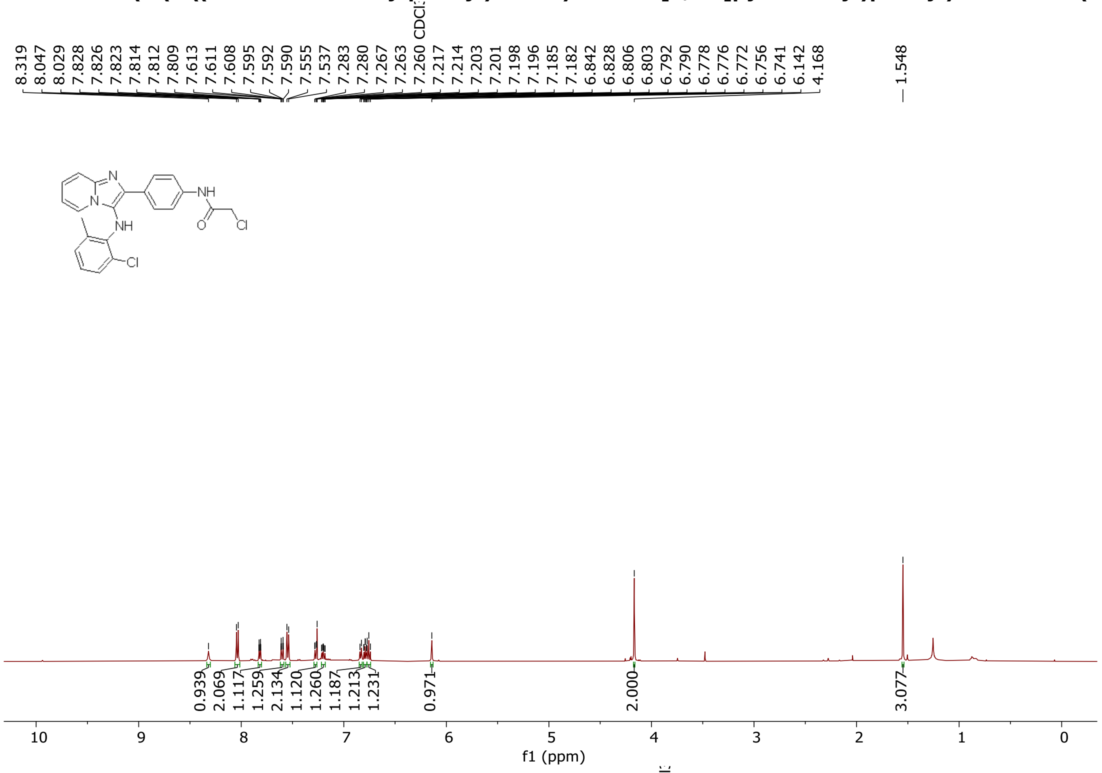

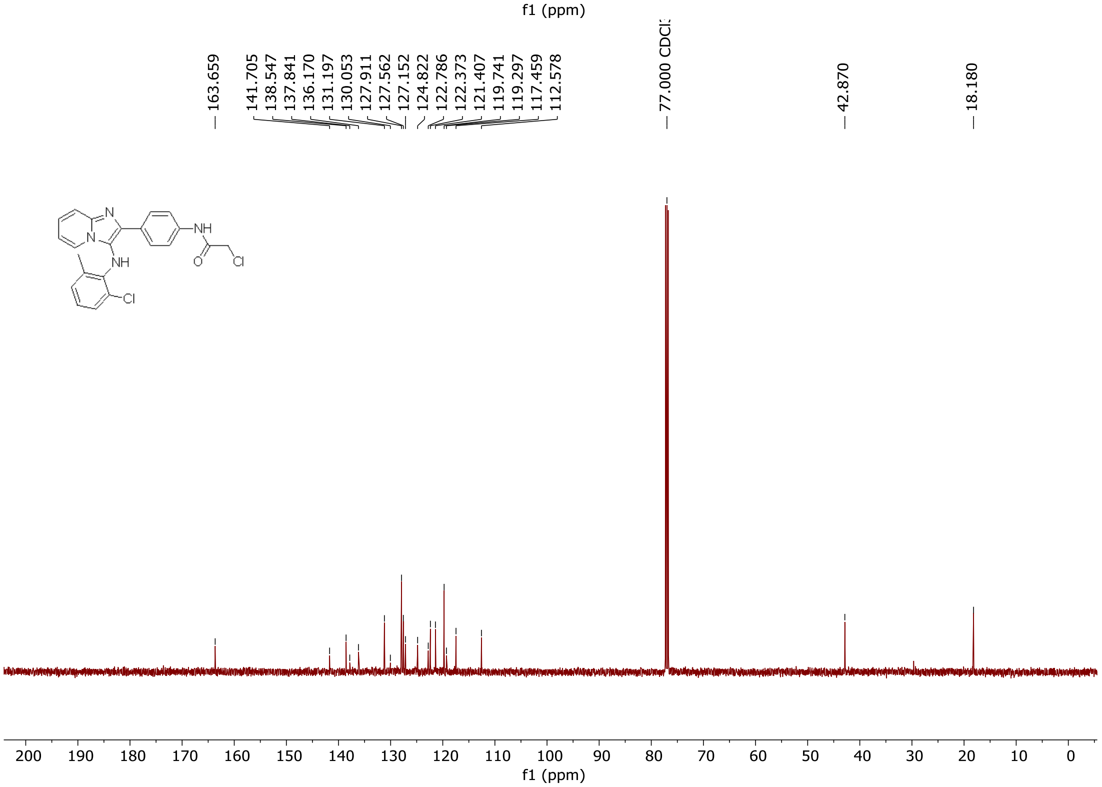

2-chloro-*N*-(4-(3-((2,4-dichlorocyclohexa-2,4-dien-1-yl)amino)imidazo[1,2-a]pyridin-2-yl)phenyl)acetamide (22)
1084654

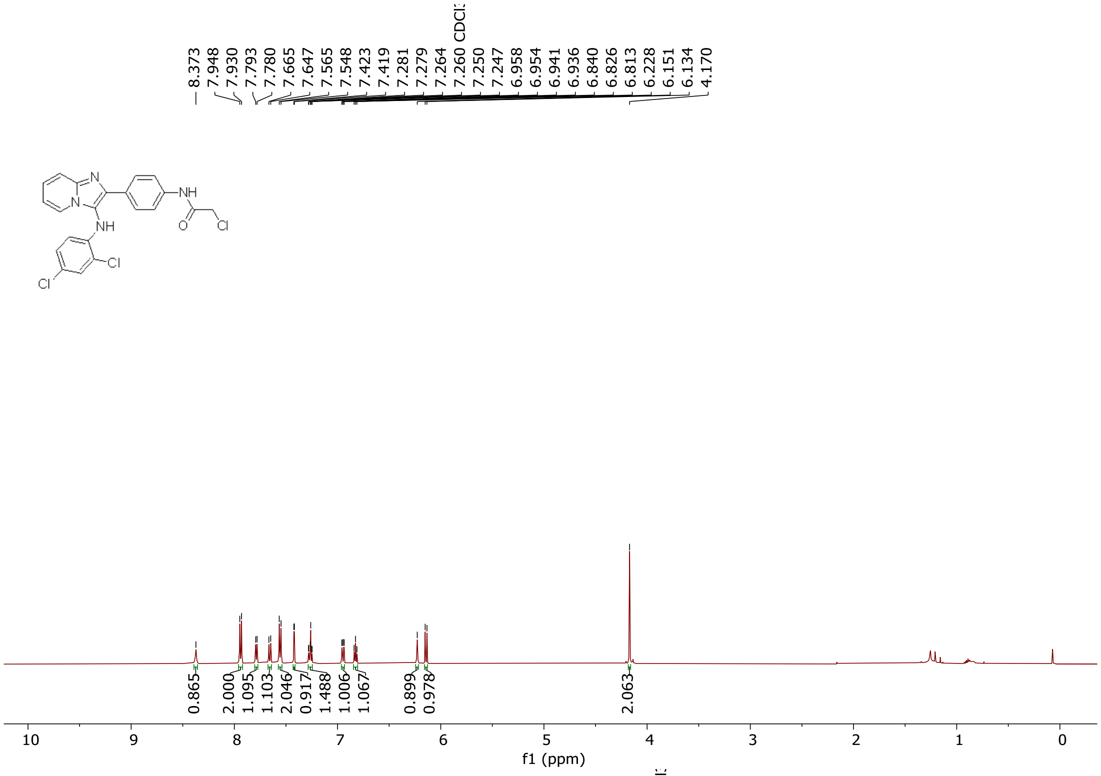

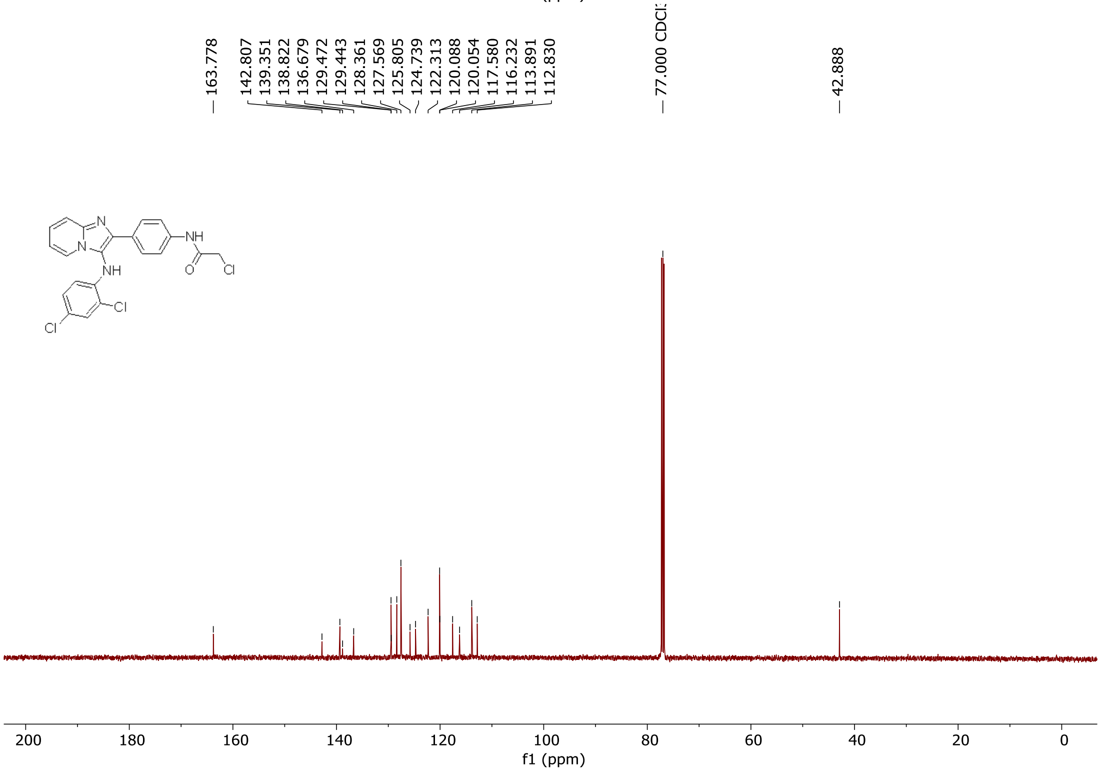

2-chloro-N-(4-(3-(mesitylamino)imidazo[1,2-a]pyridin-2-yl)phenyl)acetamide (23) 1124903

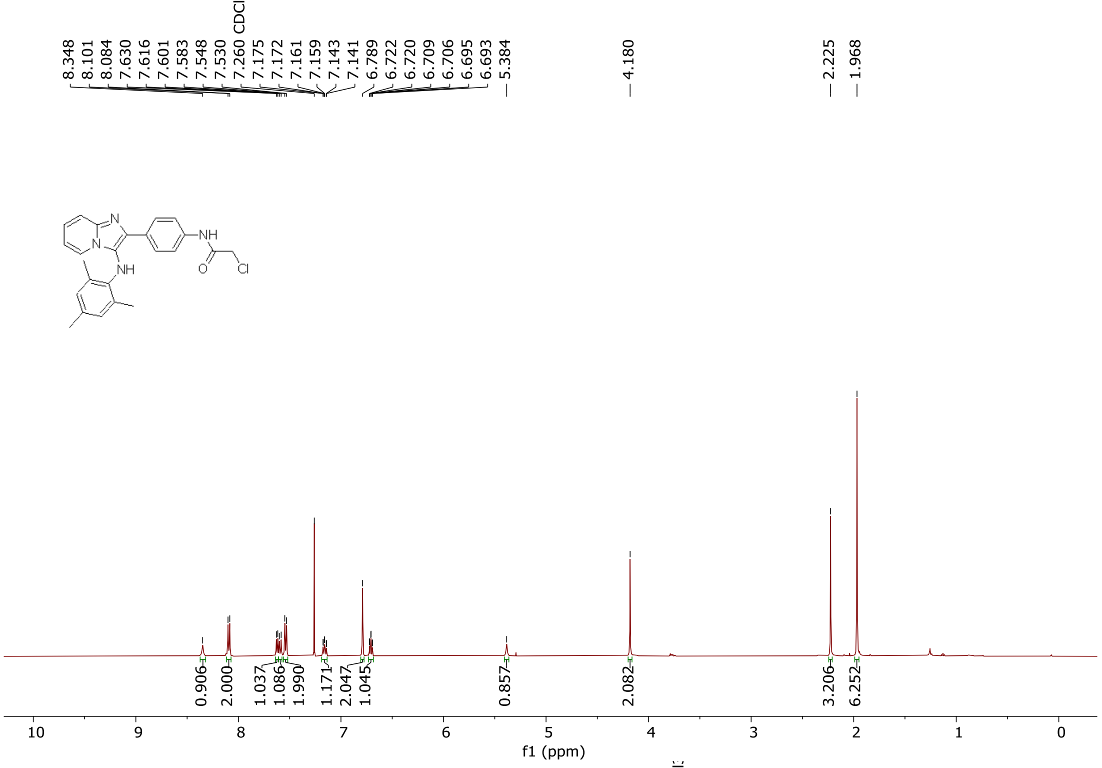

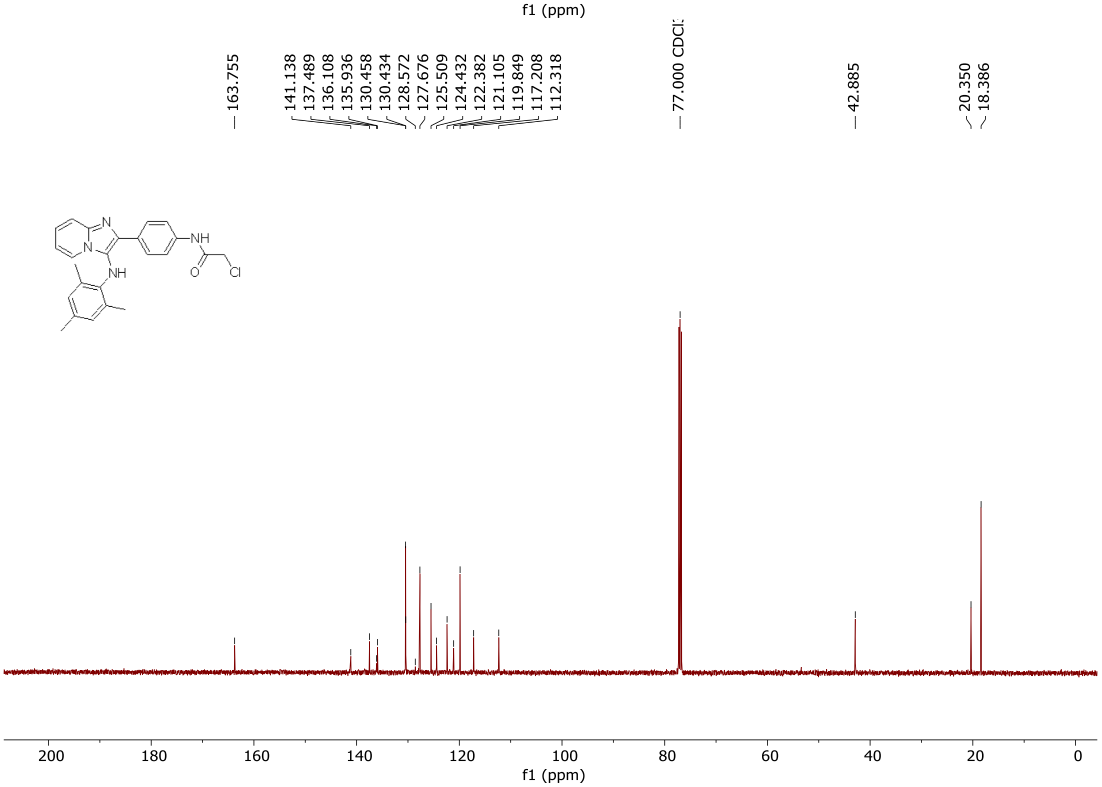

2-chloro-*N*-(4-(3-((4-chloro-2,6-dimethylphenyl)amino)imidazo[1,2-a]pyridin-2-yl)phenyl)acetamide (24)
1124908

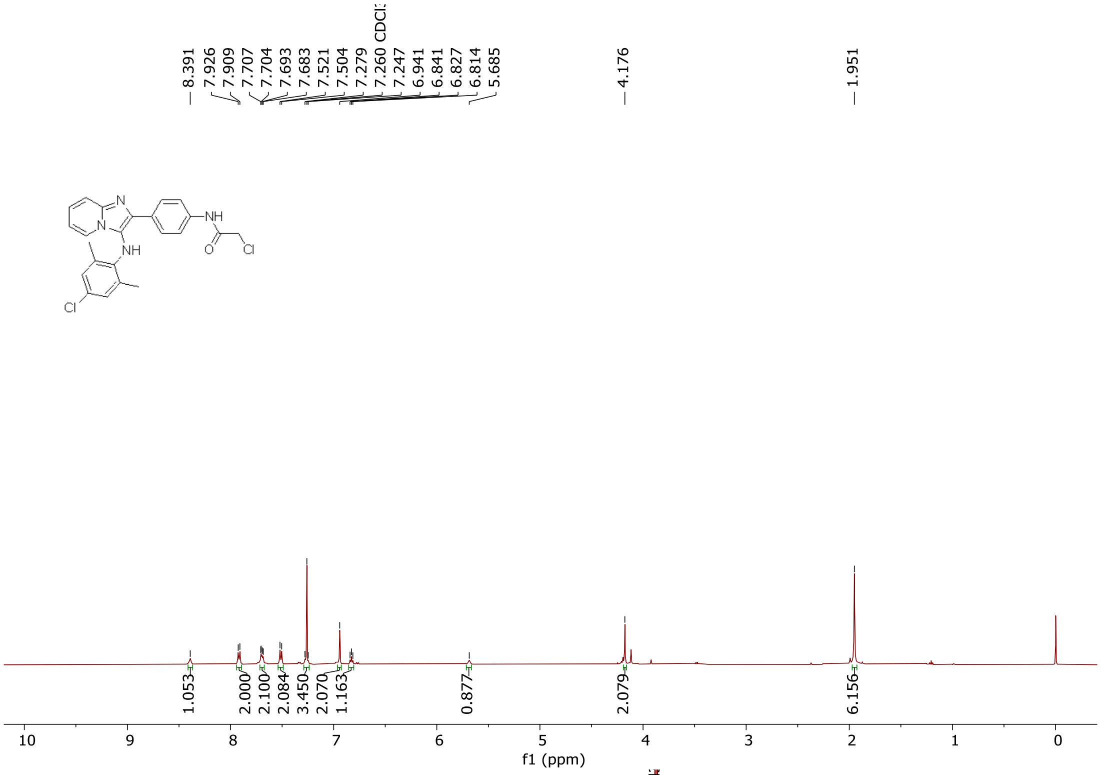

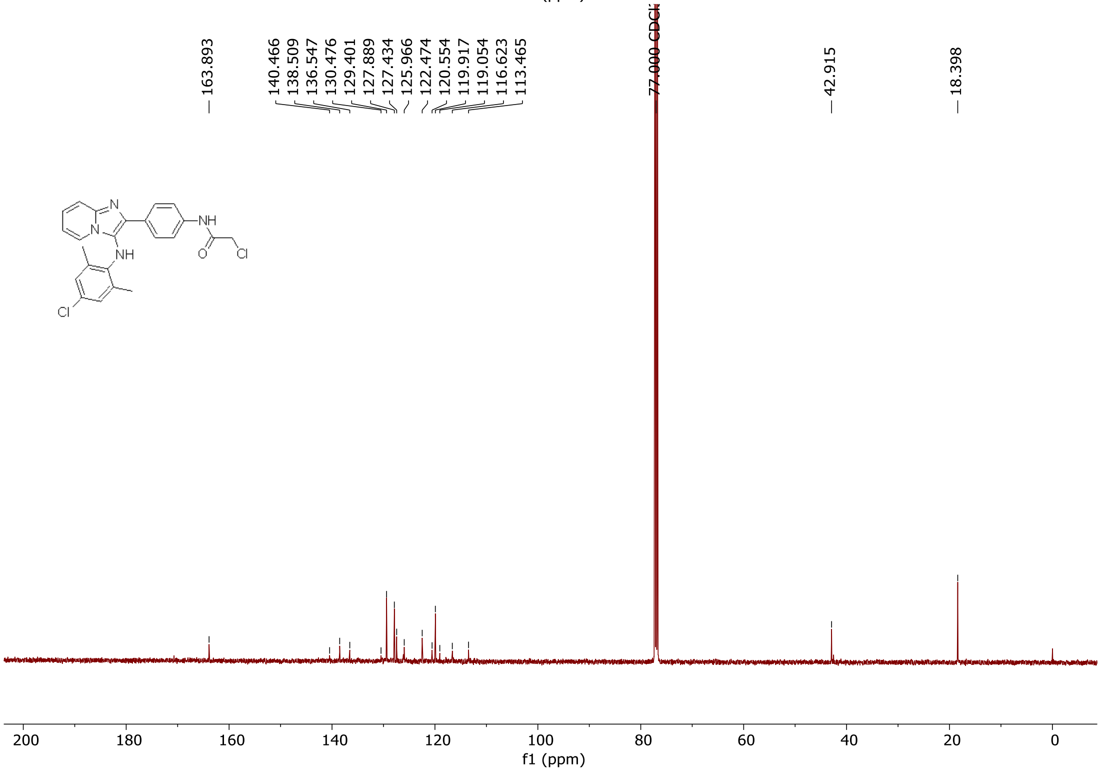

2-chloro-*N*-(4-(3-((4-hydroxy-2,6-dimethylphenyl)amino)imidazo[1,2-a]pyridin-2-yl)phenyl)acetamide (25)
1124909

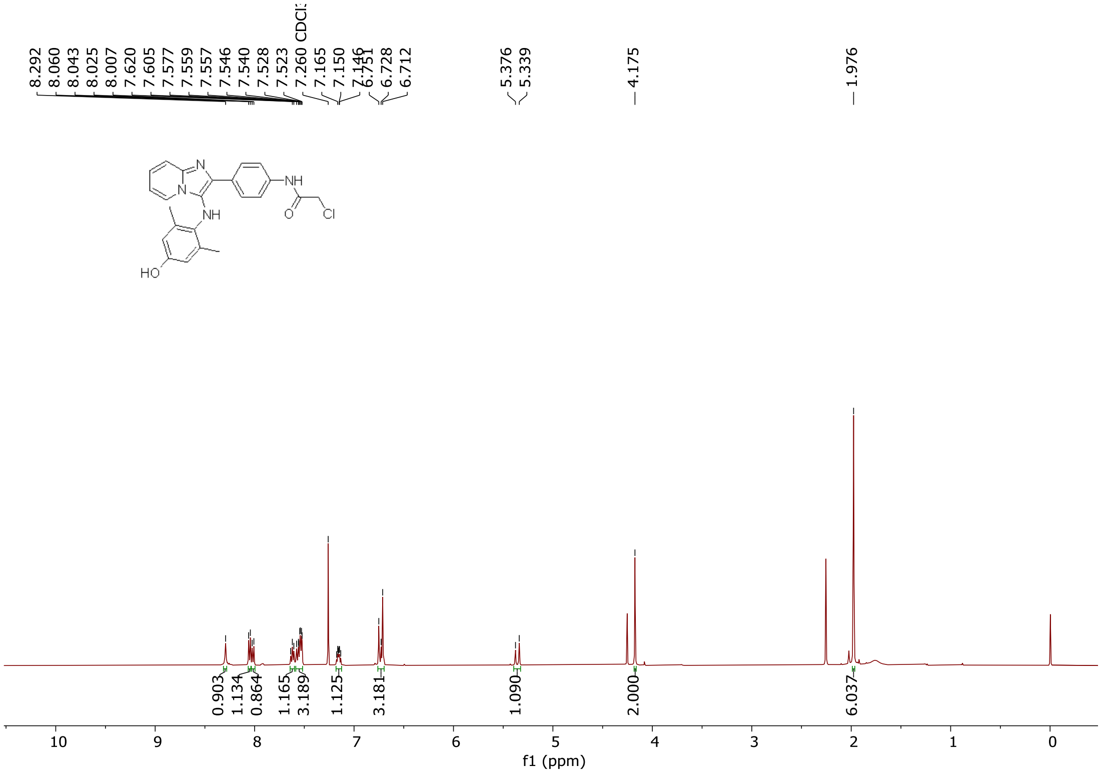

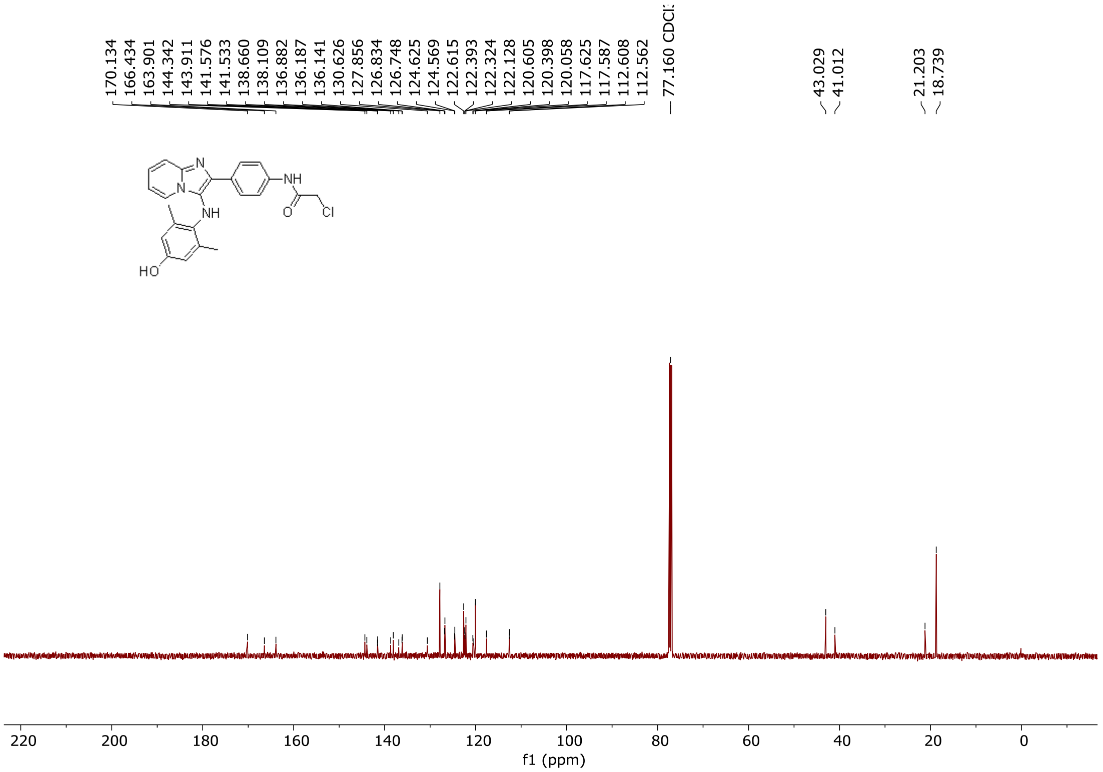

2-chloro-*N*-(4-(3-((2-chloro-6-methylphenyl)amino)-6-fluoroimidazo[1,2-a]pyridin-2-yl)phenyl)acetamide (26)
1132336

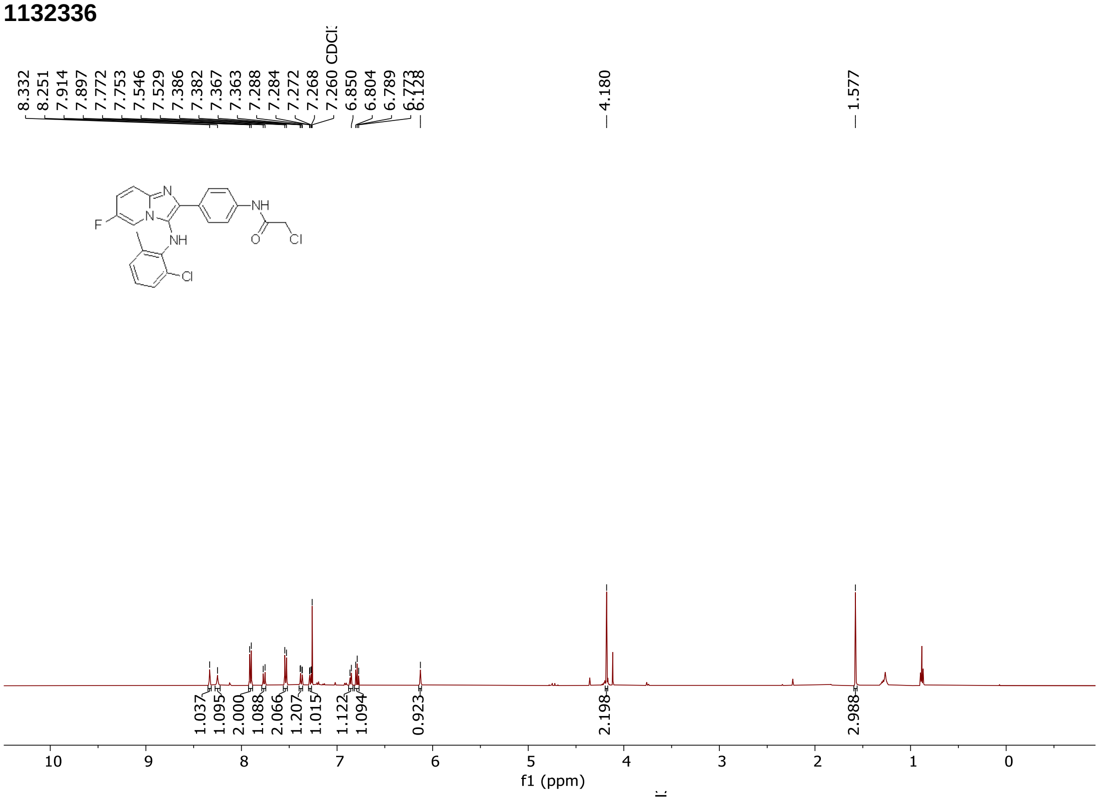

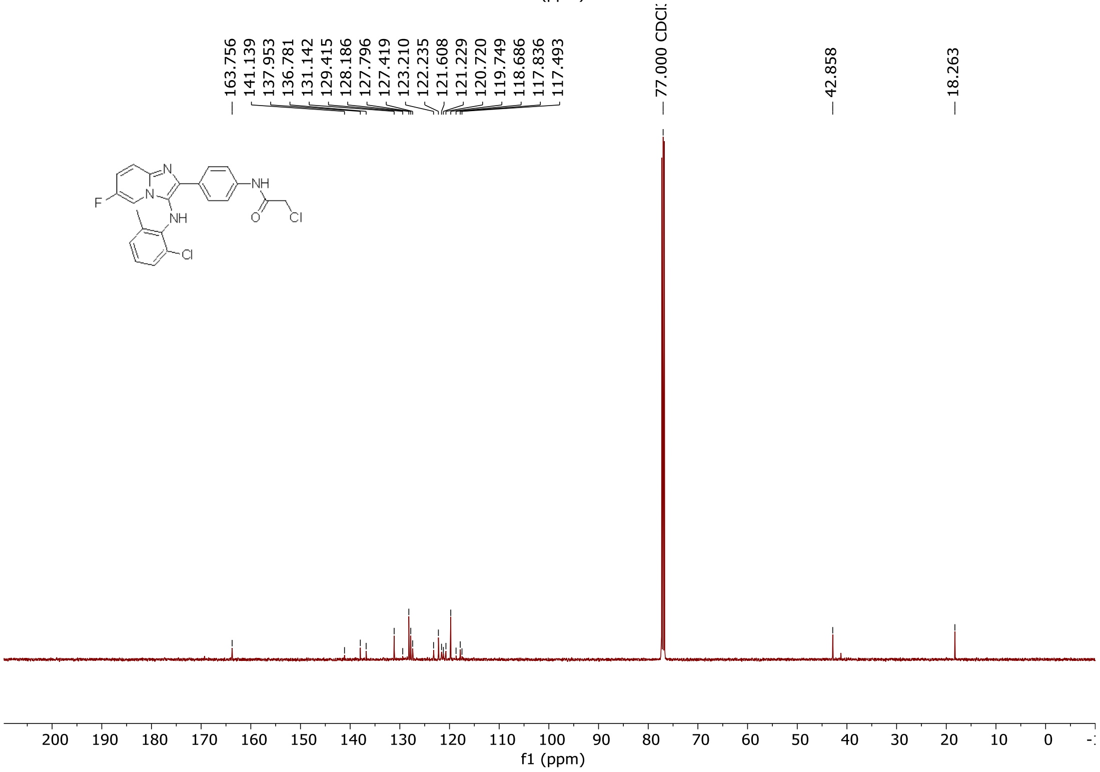

2-chloro-*N*-(4-(6-chloro-3-((2-chloro-6-methylphenyl)amino)imidazo[1,2-a]pyridin-2-yl)phenyl)acetamide (27)
1132335

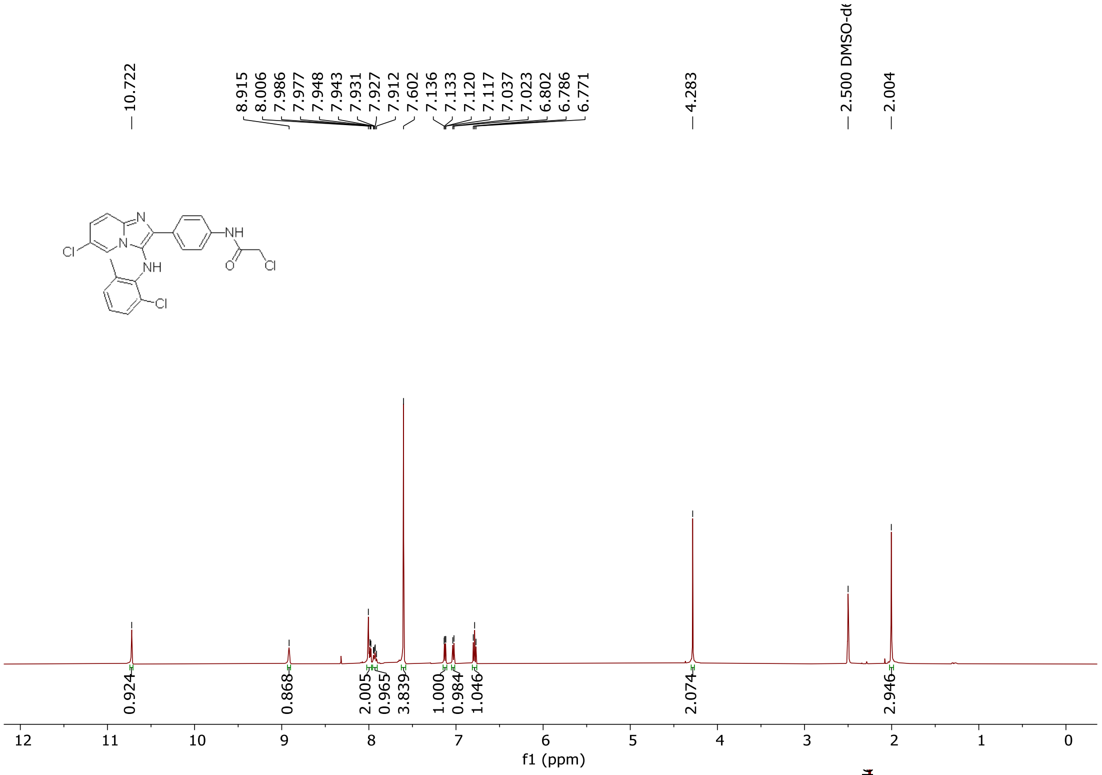

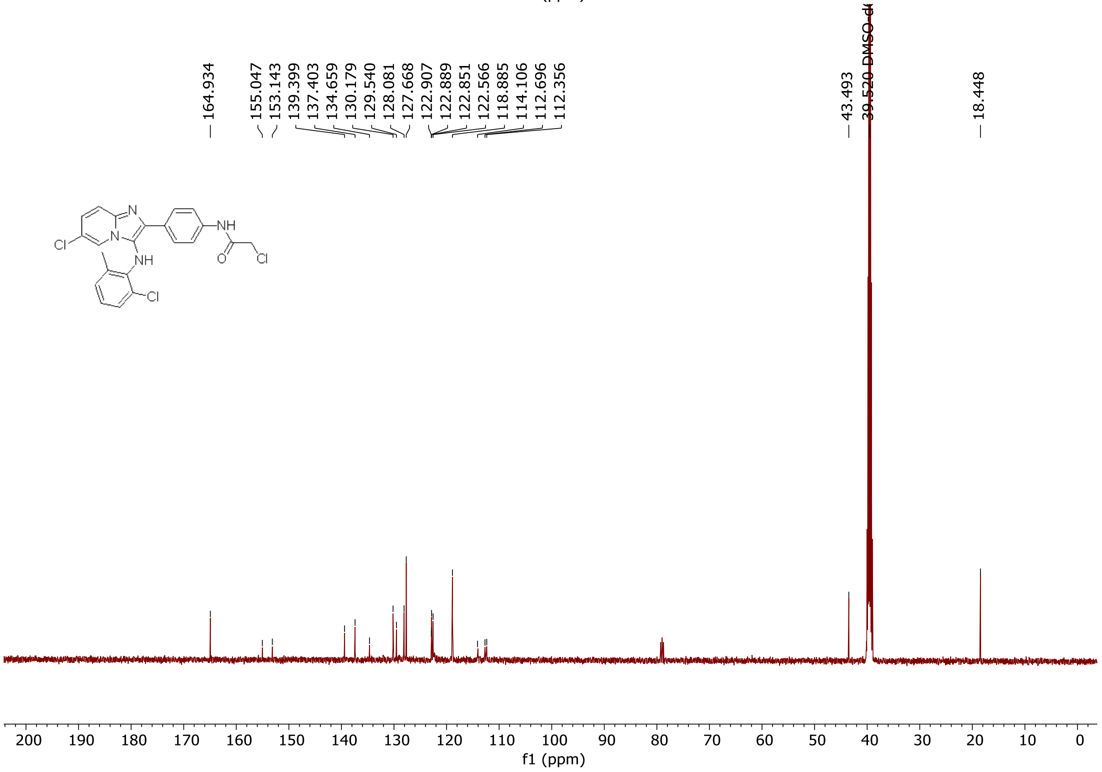

2-chloro-N-(4-(3-((2-chloro-6-methylphenyl)amino)-6-methylimidazo[1,2-a]pyridin-2-yl)phenyl)acetamide (28)
1132334

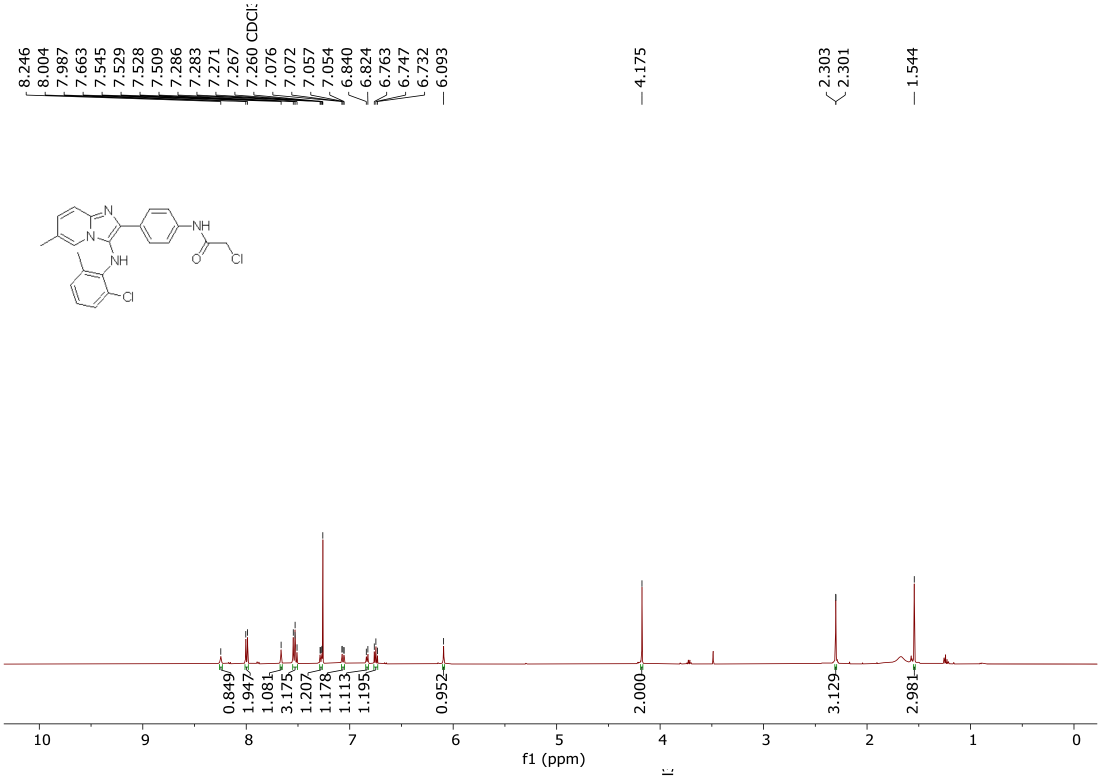

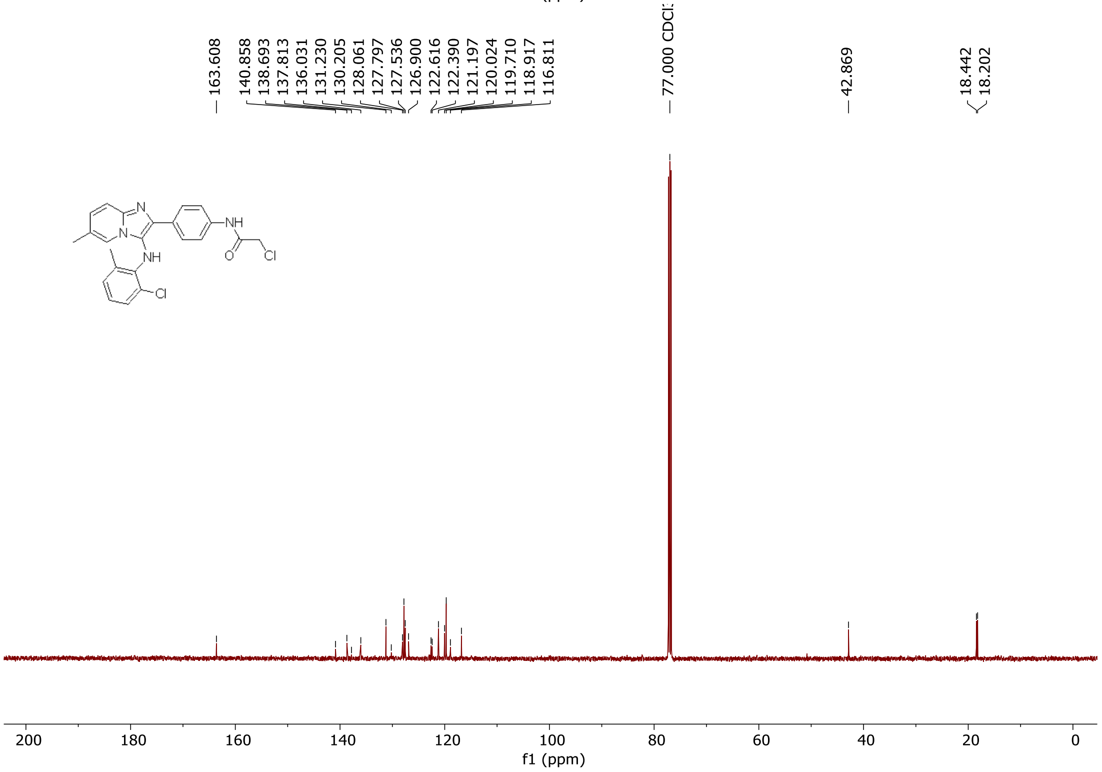

2-chloro-*N*-(4-(3-((2-chloro-6-methylphenyl)amino)-6-isopropylimidazo[1,2-a]pyridin-2-yl)phenyl)acetamide (29) 1132332

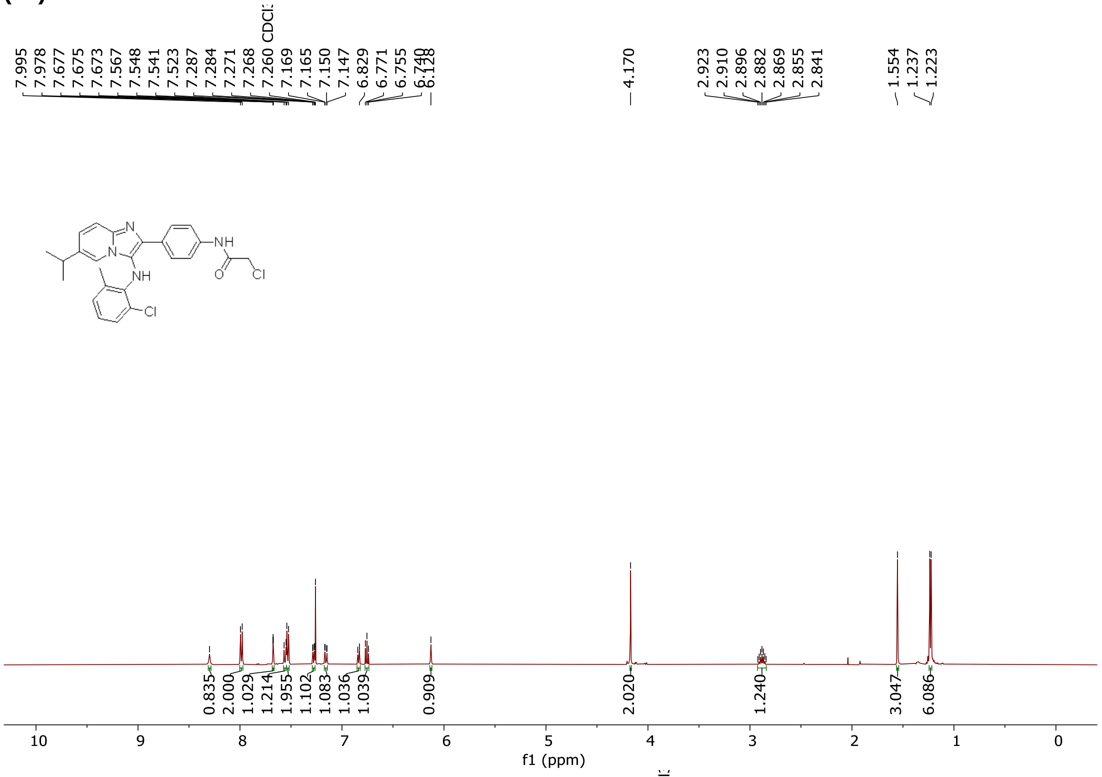

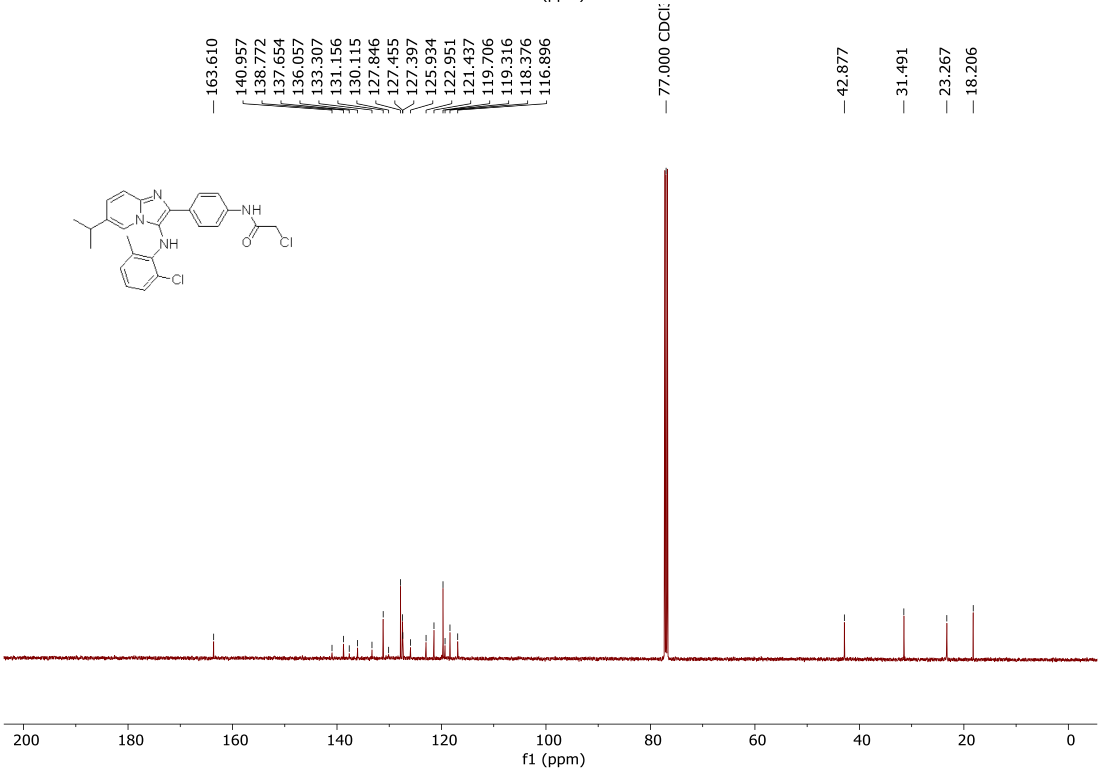

2-chloro-*N*-(4-(3-((2-chloro-6-methylphenyl)amino)imidazo[1,2-a]pyridin-2-yl)-3-fluorophenyl)acetamide (31) 1132337

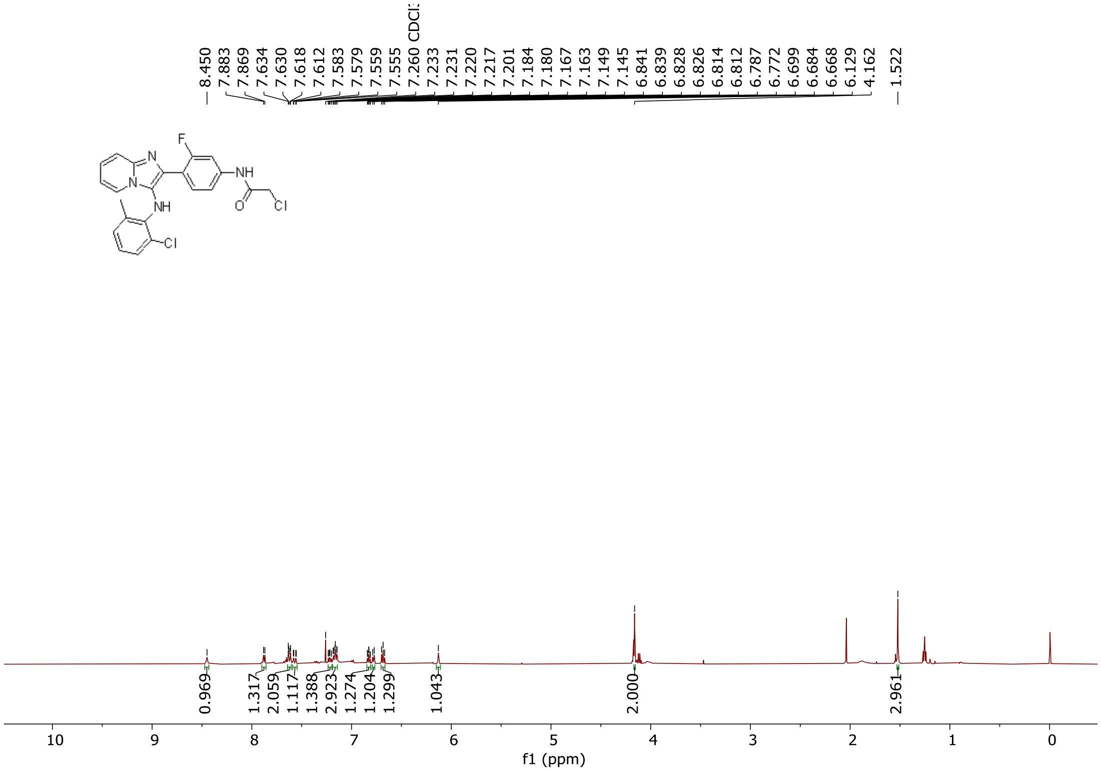

2-chloro-*N*-(4-(3-((2-chloro-6-methylphenyl)amino)imidazo[1,2-a]pyridin-2-yl)benzyl)acetamide (32) 1132338

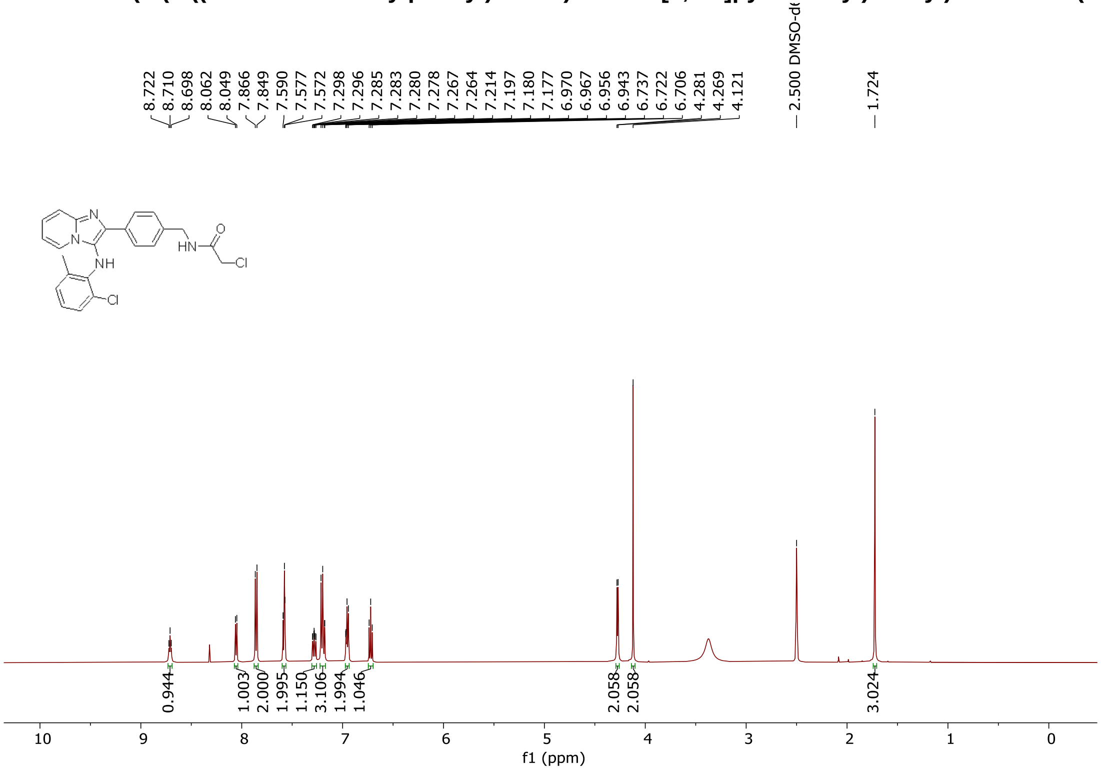

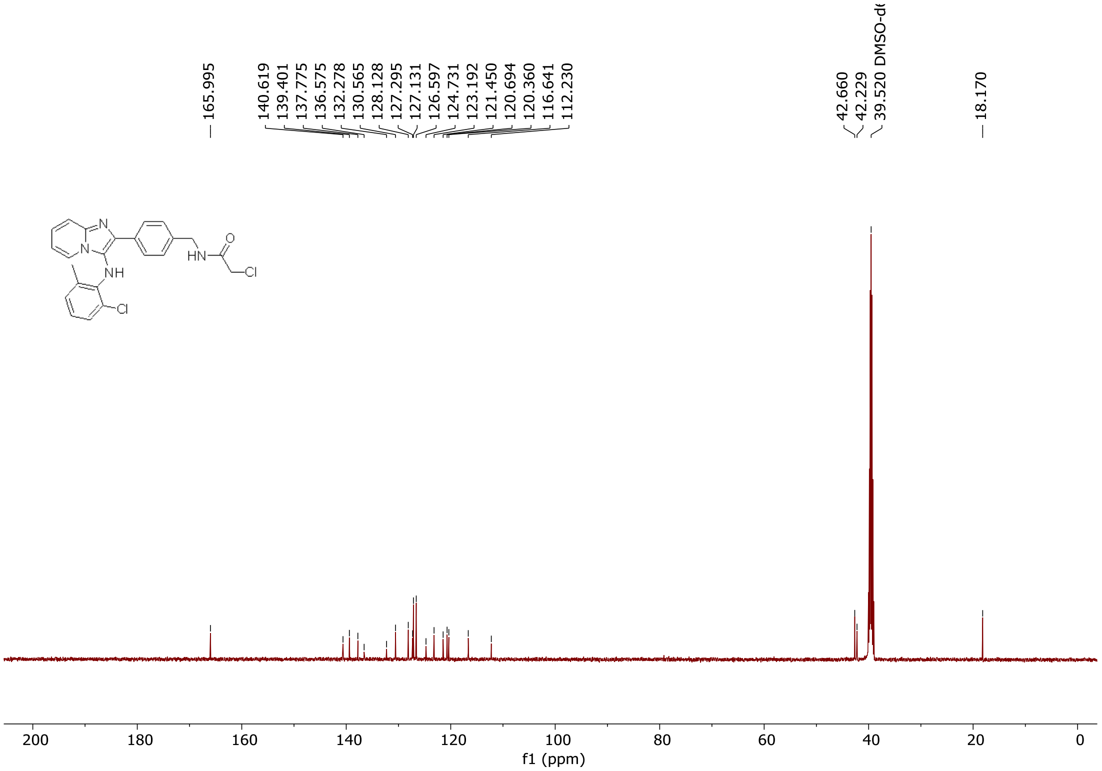

2-chloro-*N*-(4-(3-((2-chloro-6-methylphenyl)amino)imidazo[1,2-a]pyridin-2-yl)cyclohexyl)acetamide (33) 1132339

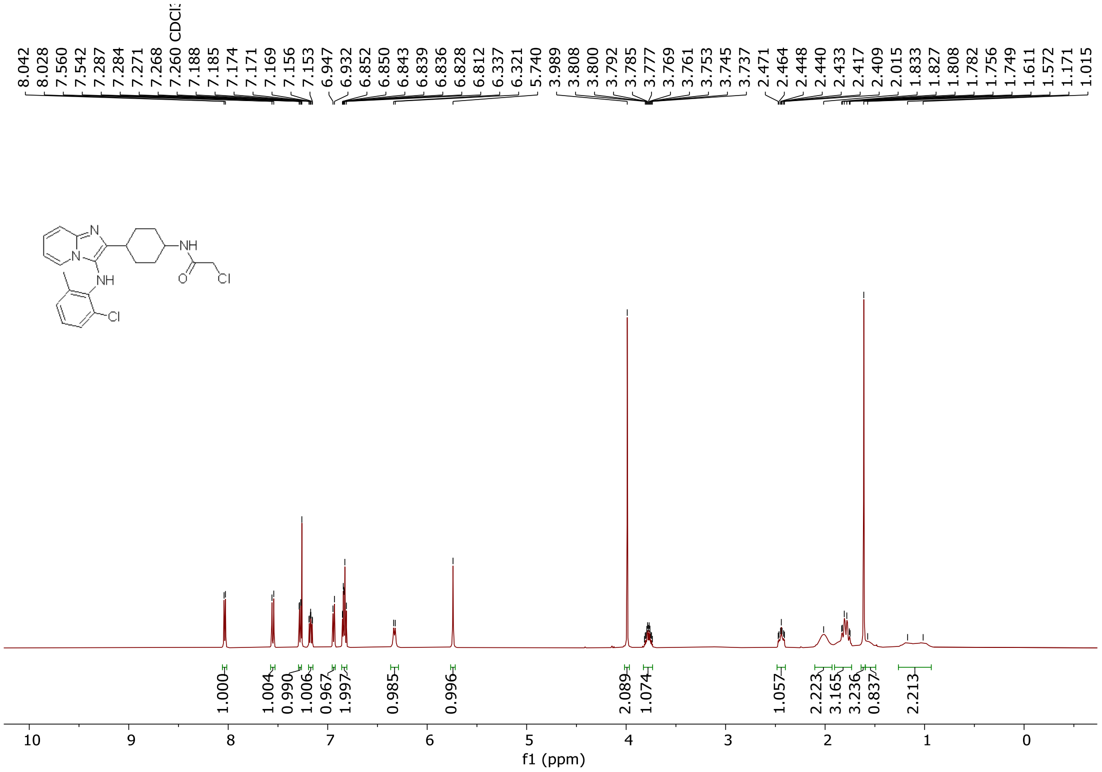

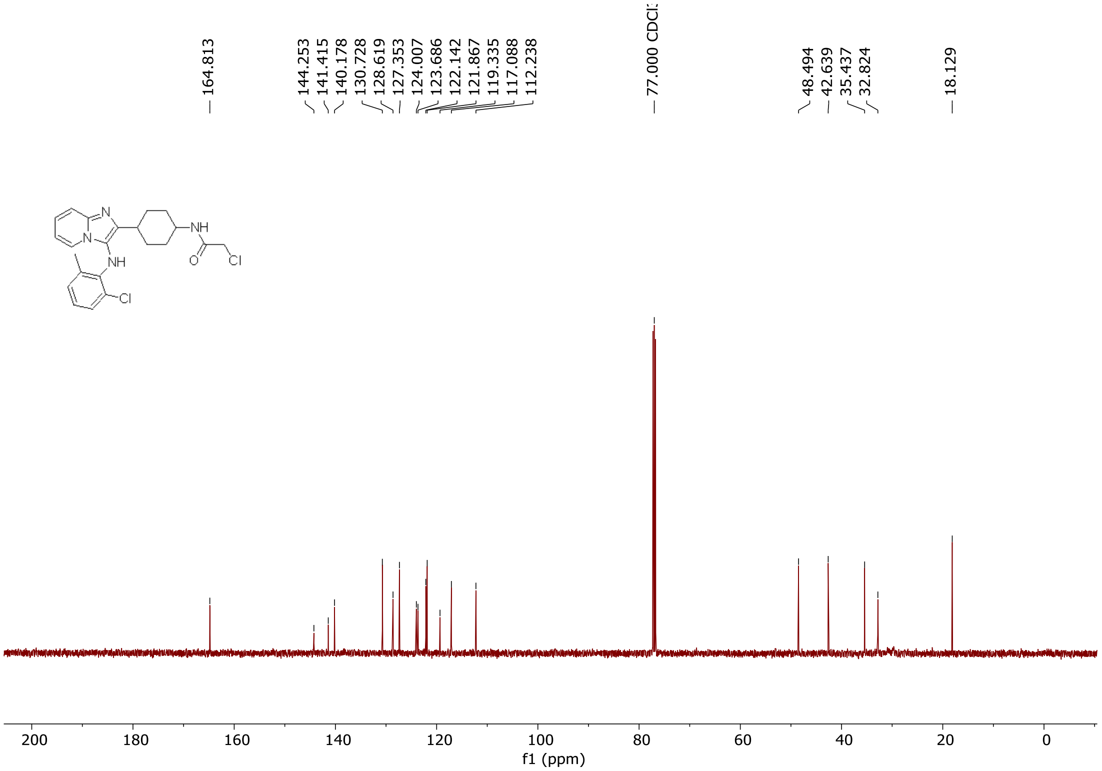

2-chloro-*N*-(4-(3-((2-chloro-6-methylphenyl)amino)imidazo[1,2-a]pyridin-2-yl)phenyl)-2,2-difluoroacetamide (35) 1132340

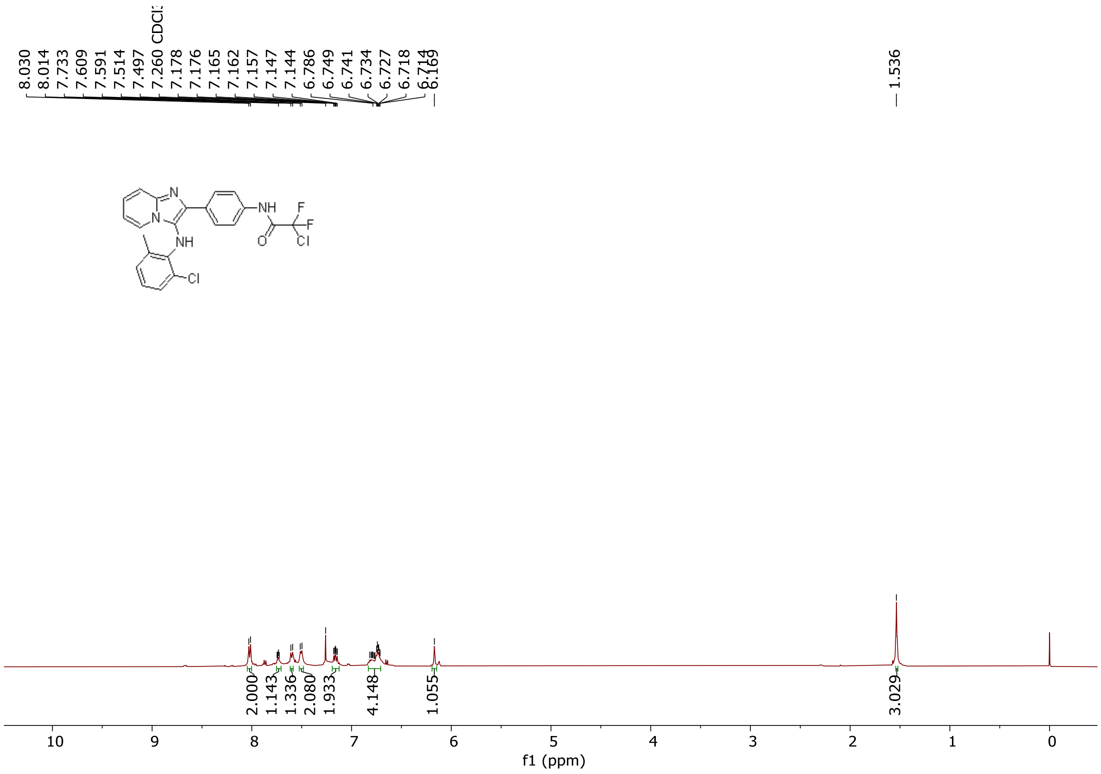

2,2,2-tribromo-*N*-(4-(3-((2-chloro-6-methylphenyl)amino)imidazo[1,2-a]pyridin-2-yl)phenyl)acetamide (36)
1132342

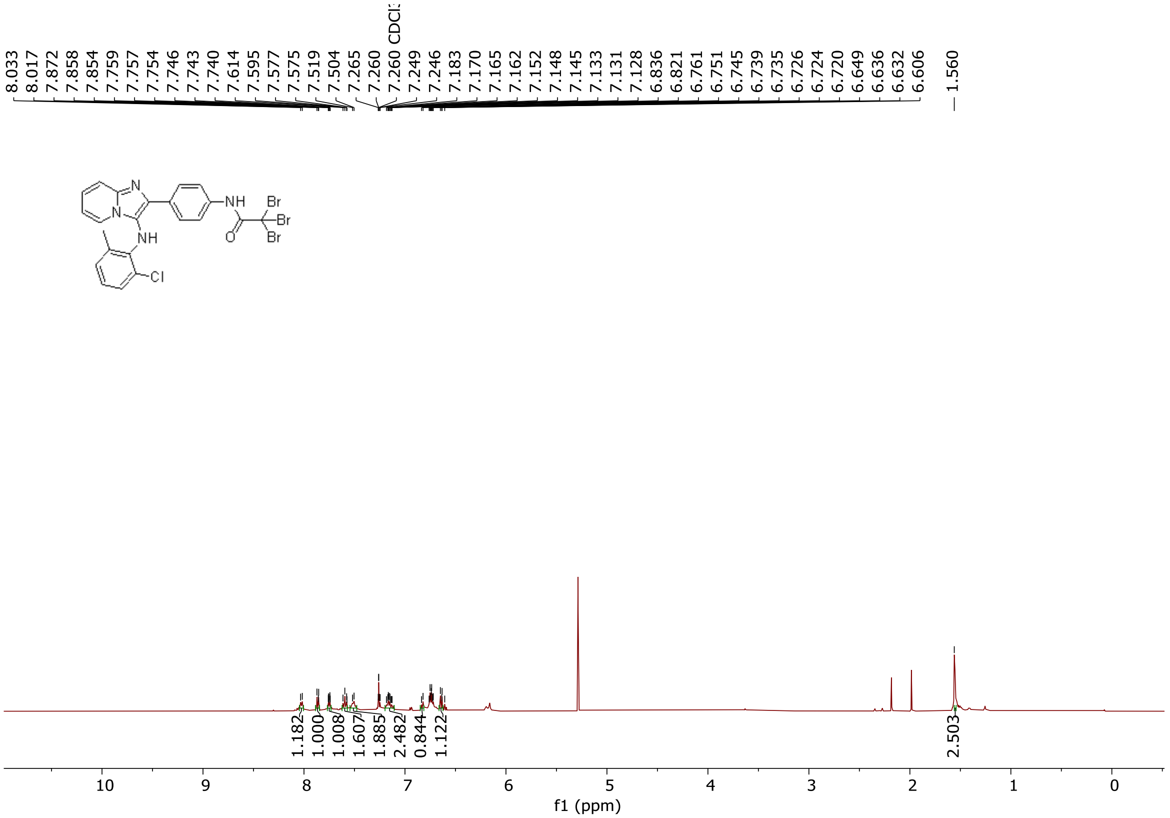

2-chloro-N-(4-(3-((2-chloro-6-methylphenyl)amino)imidazo[1,2-a]pyridin-2-yl)phenyl)-2-fluoroacetamide (37)
1132343

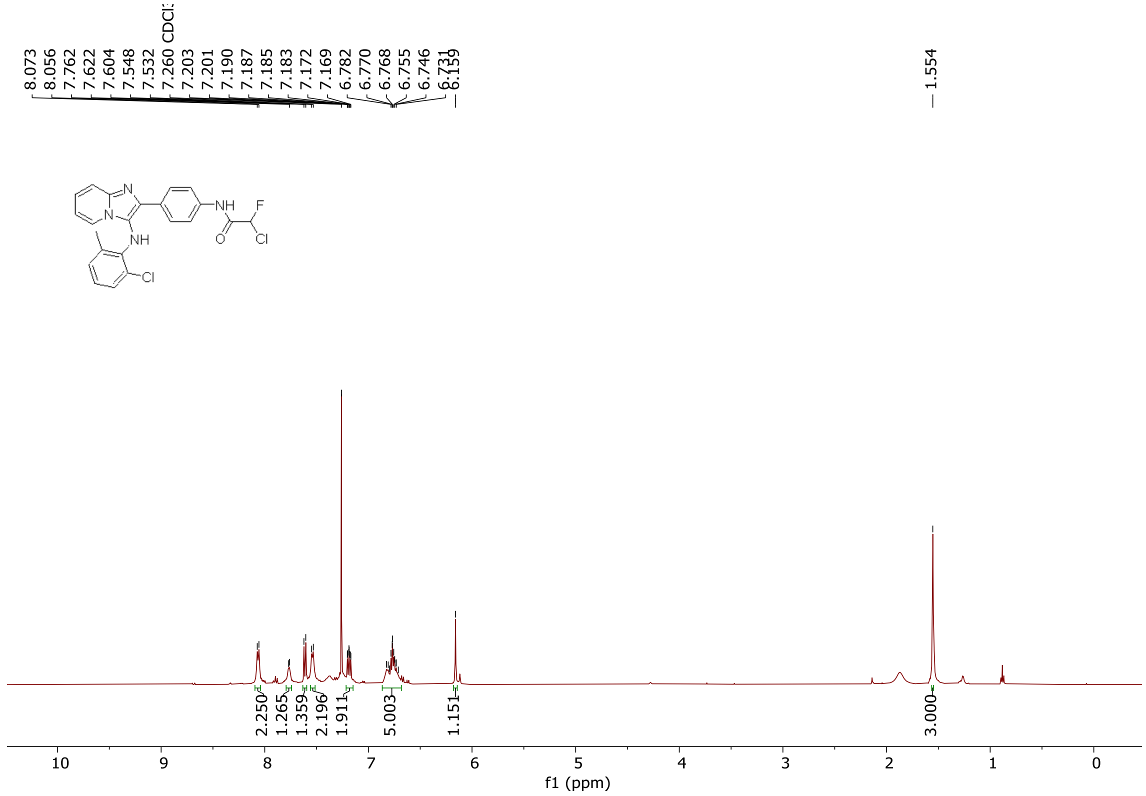

2-chloro-N-(4-(3-((2,6-dimethylphenyl)amino)-6-methylimidazo[1,2-a]pyridin-2-yl)phenyl)acetamide (39)
1132364

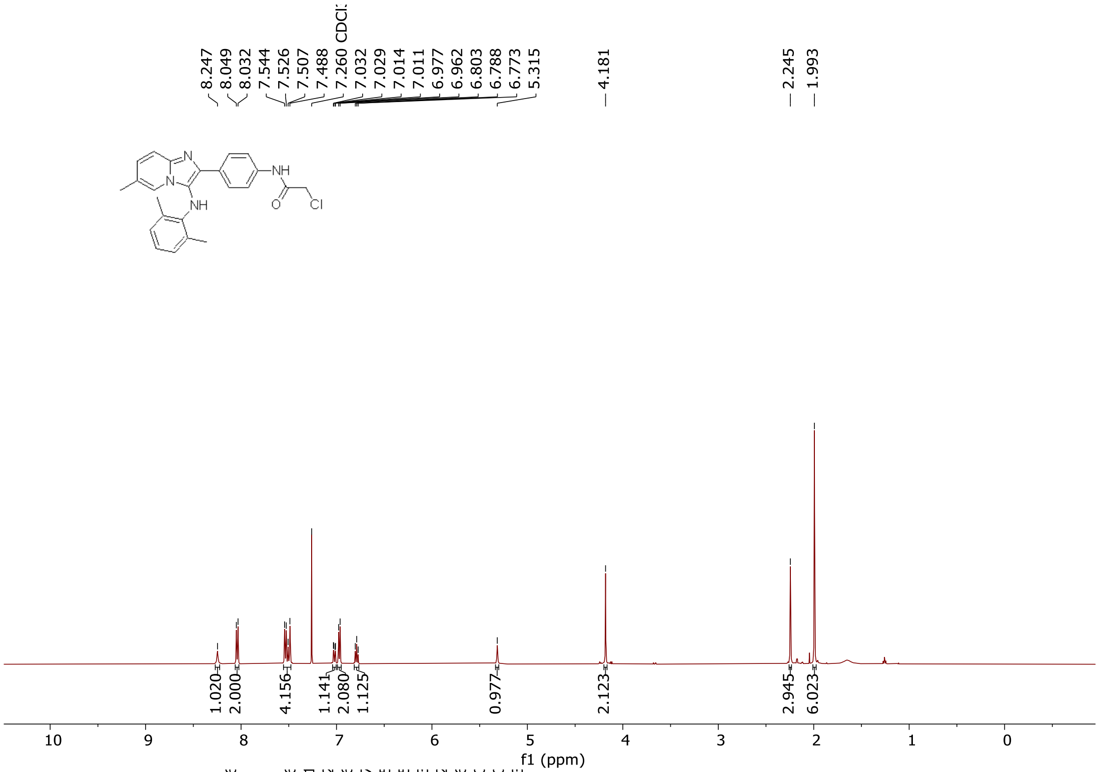

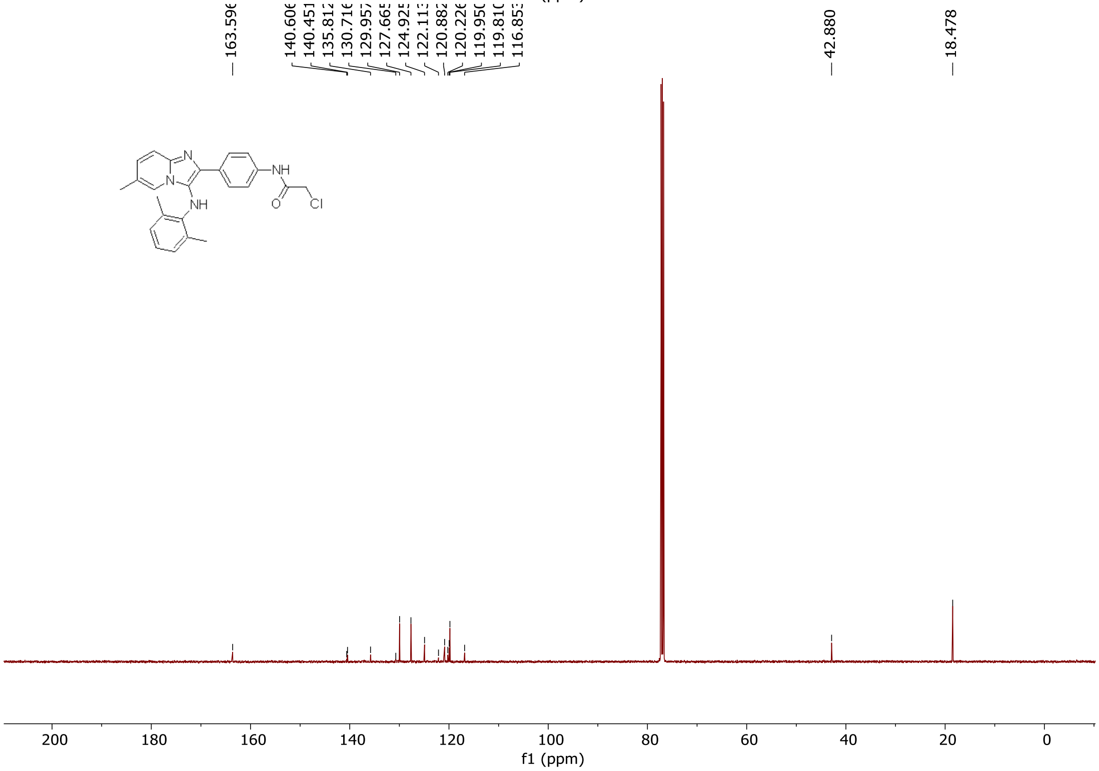

2-chloro-N-(4-(3-((2,6-dimethylphenyl)amino)imidazo[1,2-a]pyridin-2-yl)-3-fluorophenyl)acetamide (40)
1132365

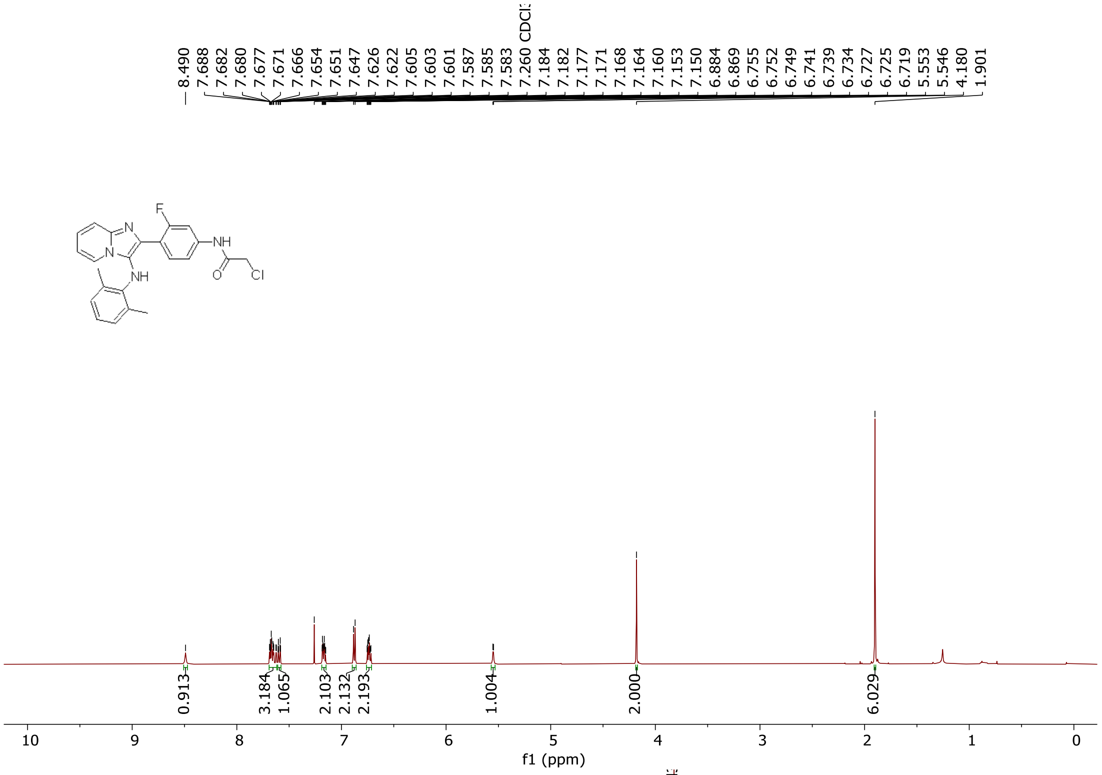

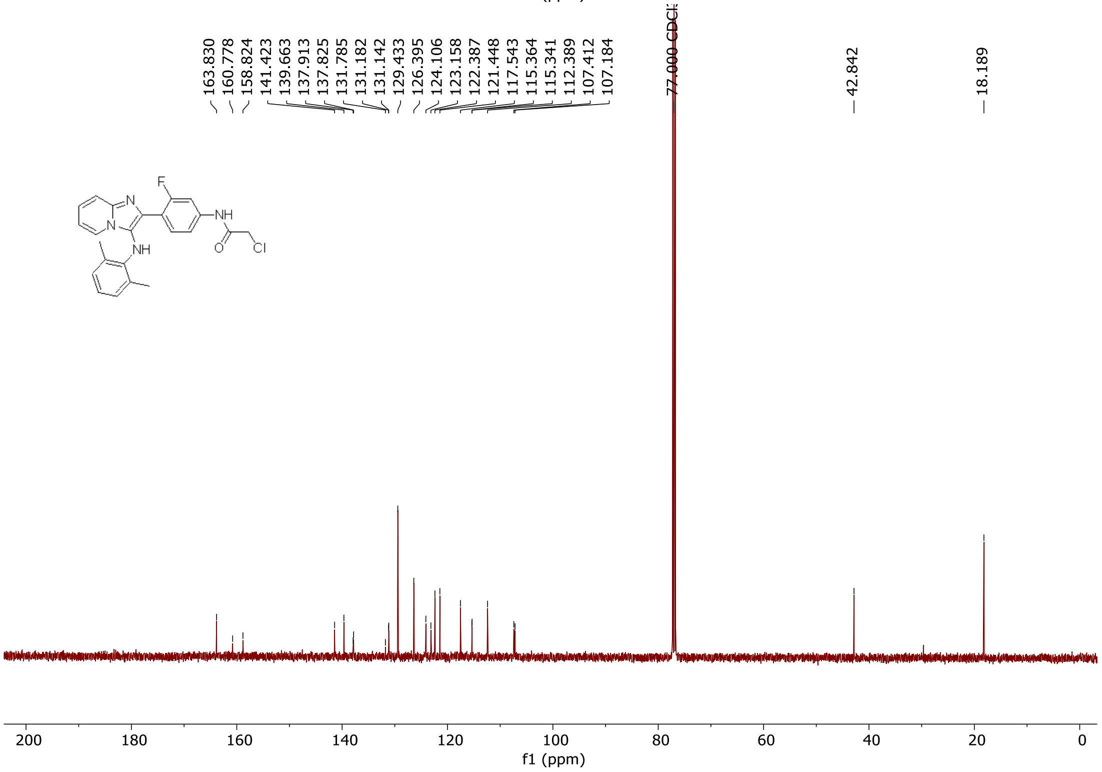

2-chloro-N-(4-(3-((2,6-dimethylphenyl)amino)-6-methylimidazo[1,2-a]pyridin-2-yl)-3-fluorophenyl)acetamide (41) 1132366

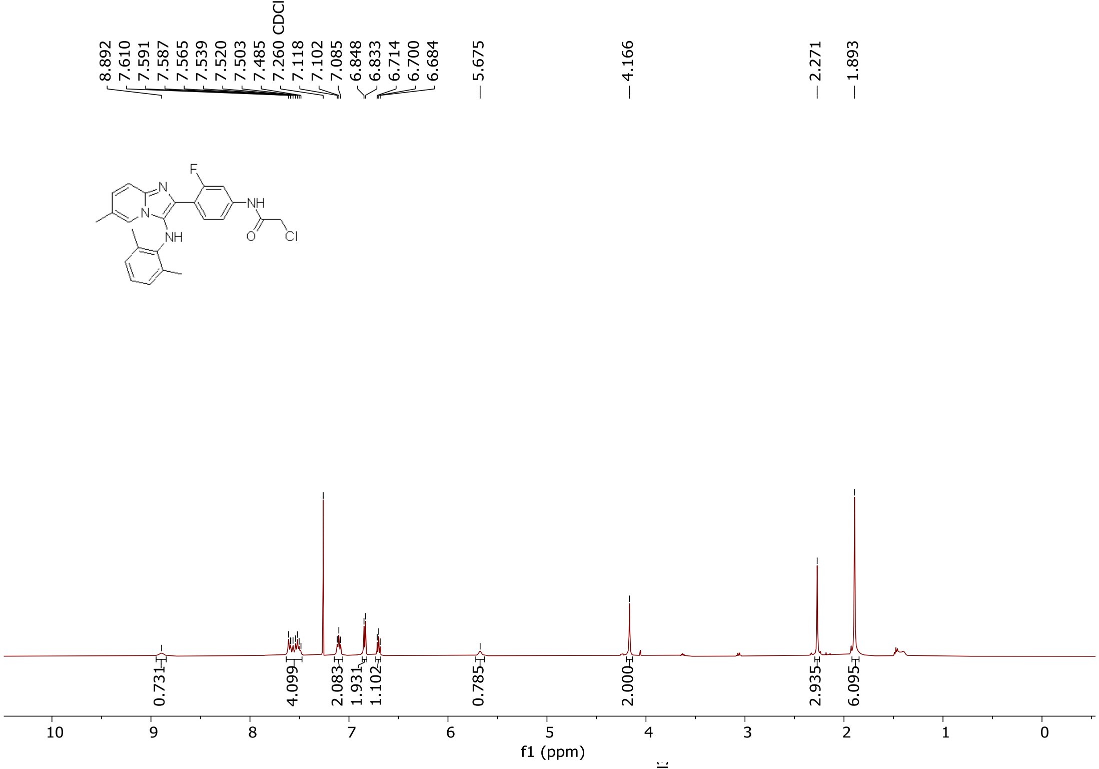

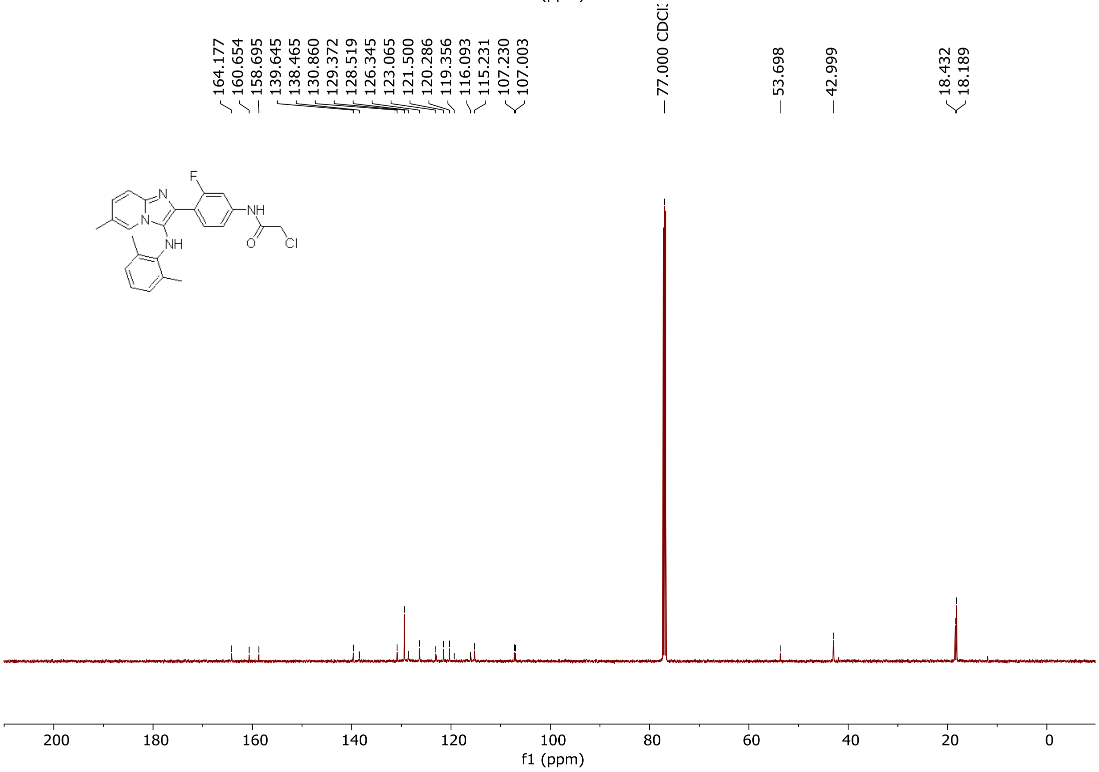

2-chloro-N-(4-(6-chloro-3-((2,6-dimethylphenyl)amino)imidazo[1,2-a]pyridin-2-yl)-3-fluorophenyl)acetamide (42) 1132367

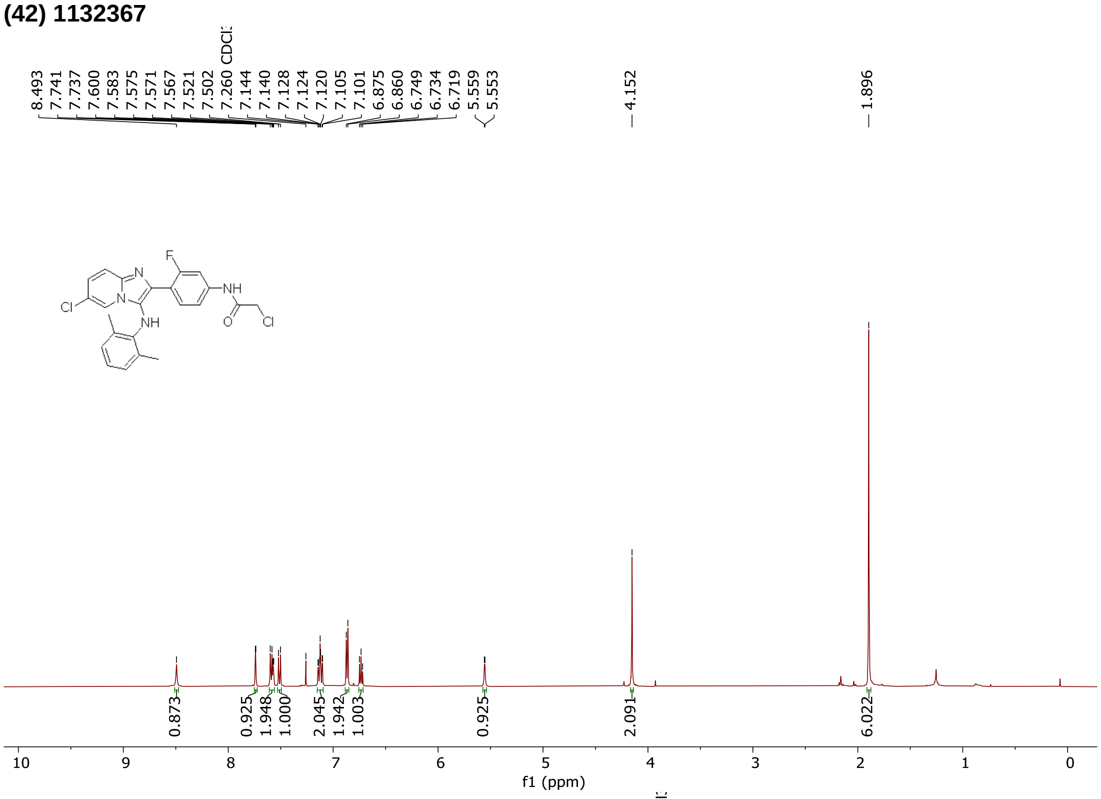

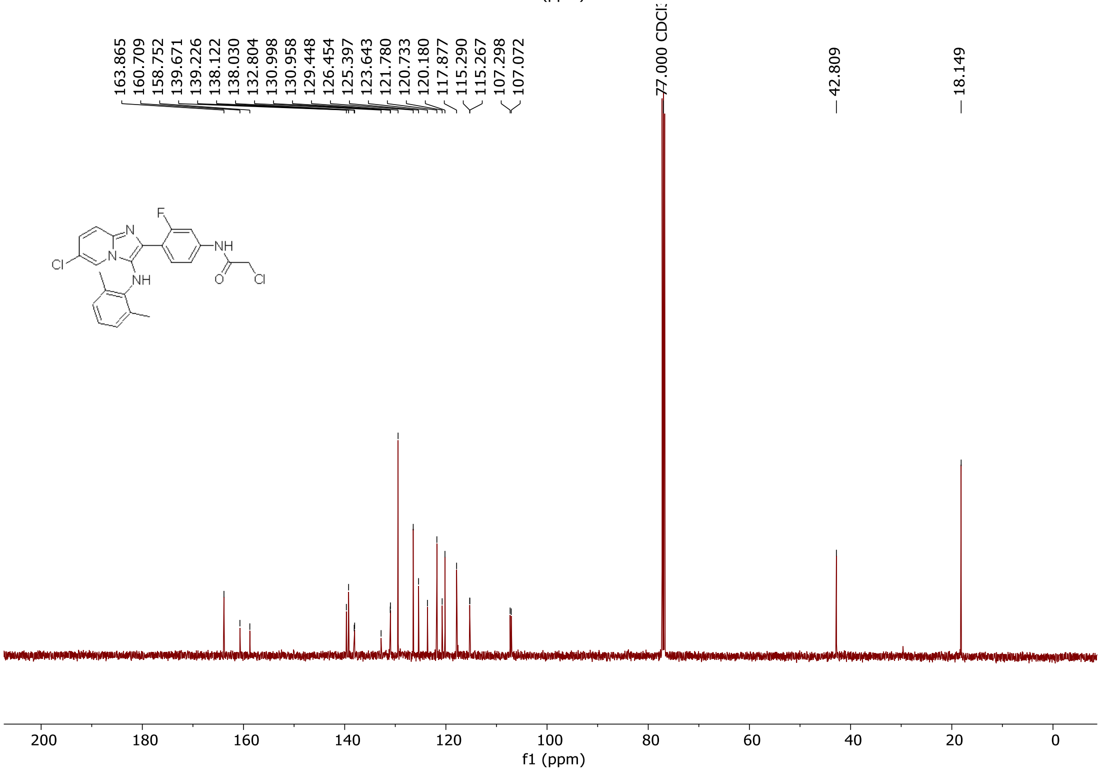

Supplement: Supplementary file 1 — Supplementary Information [file 41467_2025_61176_MOESM1_ESM.pdf]
